# Mechanical Bond Enhanced Lithium Halide Ion‐Pair Binding by Halogen Bonding Heteroditopic Rotaxanes**

**DOI:** 10.1002/chem.202201209

**Published:** 2022-07-06

**Authors:** Vihanga K. Munasinghe, Jessica Pancholi, Dilhan Manawadu, Zongyao Zhang, Paul D. Beer

**Affiliations:** ^1^ Department of Chemistry University of Oxford Chemistry Research Laboratory Mansfield Road Oxford OX13TA UK; ^2^ Department of Chemistry University of Oxford Physical and Theoretical Chemistry Laboratory Oxford OX13QZ UK

**Keywords:** halogen bond, heteroditopic rotaxane, hydrogen bond, Ion-pair, lithium halide

## Abstract

A family of novel halogen bonding (XB) and hydrogen bonding (HB) heteroditopic [2]rotaxane host systems constructed by active metal template (AMT) methodology, were studied for their ability to cooperatively recognise lithium halide (LiX) ion‐pairs. ^1^H NMR ion‐pair titration experiments in CD_3_CN:CDCl_3_ solvent mixtures revealed a notable “switch‐on“ of halide anion binding in the presence of a co‐bound lithium cation, with rotaxane hosts demonstrating selectivity for LiBr over LiI. The strength of halide binding was shown to greatly increase with increasing number of halogen bond donors integrated into the interlocked cavity, where an all‐XB rotaxane was found to be the most potent host for LiBr. DFT calculations corroborated these findings, determining the mode of LiX ion‐pair binding. Notably, ion‐pair binding was not observed with the corresponding XB/HB macrocycles alone, highlighting the cooperative, heteroditopic, rotaxane axle‐macrocycle component mechanical bond effect as an efficient strategy for ion‐pair recognition in general.

## Graphical abstract

**The first examples** of selective lithium halide recognition by a mechanical bonded heteroditopic host is achieved using a series of halogen and hydrogen bonding [2]rotaxanes. A co‐bound lithium cation switches on halide anion binding which increases greatly with increasing number of halogen bond donors. Ion‐pair binding was not observed with the corresponding heteroditopic macrocycles, highlighting the importance of the mechanical bond effect.

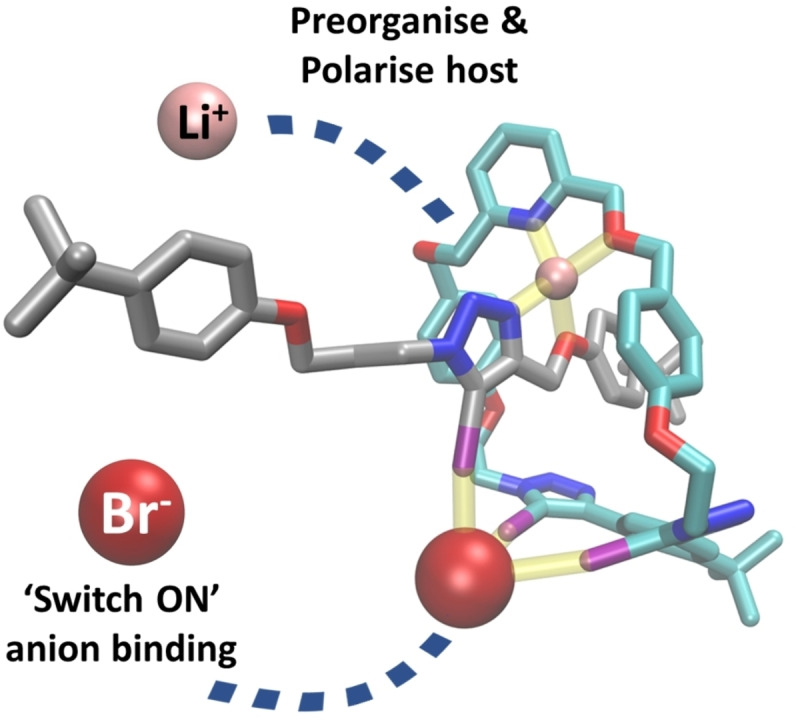

## Introduction

The fundamental roles of cation and anion species in a range of biological, industrial and environmental processes is the stimulus for the ever increasing demand to design and develop selective abiotic receptors.[^1^, ^2^, ^3^, ^4^, ^5^] Due to their unique topologies and multidentate three‐dimensional binding cavities, mechanically interlocked molecules^[6]^ (MIMs) have become increasingly popular as hosts for charged guest molecular recognition applications. In particular, the interlocked preorganised solvophobic binding sites can provide shape and size complementarity for a wide range of cationic,[^7^, ^8^, ^9^, ^10^, ^11^, ^12^, ^13^] and anionic[^14^, ^15^, ^16^, ^17^] substrates. Importantly, MIMs often display significantly enhanced selective guest binding properties compared to analogous non‐interlocked receptor counterparts.^[18]^ Heteroditopic receptors containing separate binding sites for both cations and anions, commonly exhibit augmented cooperative recognition behaviour relative to their monotopic receptor analogues.[^19^, ^20^] This is generally attributed to favourable electrostatic interactions and/or conformational effects upon proximal ion‐pair binding. Although the complexation of ion‐pairs by non‐interlocked heteroditopic receptors is now well established, mechanically bonded heteroditopic host systems for ion‐pair recognition remain scarce.[^21^, ^22^, ^23^, ^24^, ^25^] Furthermore halogen bonding (XB) heteroditopic interlocked receptors are extremely rare,^[22]^ despite the fact that XB interactions in general have shown superior anion guest binding properties compared to hydrogen bonding (HB) analogues.^[26]^

During the past two decades the worldwide demand for lithium has increased substantially due to its many applications, including lithium ion‐batteries, pharmaceuticals and modern materials.^[27]^ However, despite its ubiquity in modern life, lithium salts can cause severe toxic effects due to their impact on the central nervous system. This includes irreversible neurological impacts such as dementia, Parkinson's disease, cerebellar impairment and peripheral neuropathies.[^28^, ^29^] Recent reports show an alarming increase in lithium concentration in streams and tap water which corelates with the population density of the area.^[30]^ Therefore, the recognition of lithium salts is of great importance to minimise their environmental and biological effects. However, examples of heteroditopic receptors strategically designed to recognise LiX ion‐pair species are rare and have been restricted to acyclic and macrocyclic ditopic receptors.[^26^, ^31^, ^32^, ^33^]

**Figure 1:**
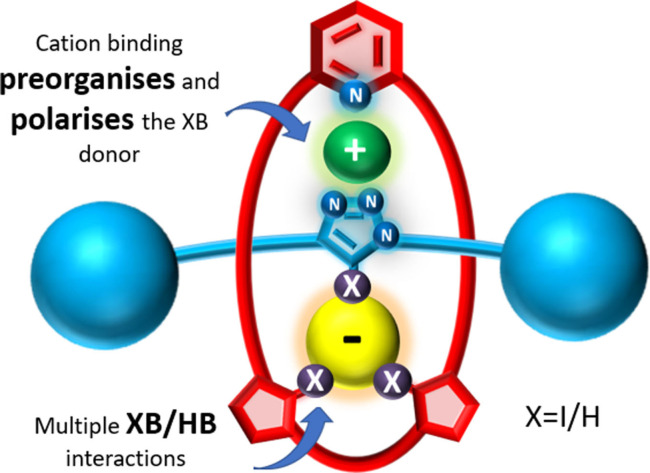
Schematic representation of the ion‐pair bound rotaxane.

Herein, we report the synthesis and ion‐pair binding properties of a series of novel heteroditopic [2]rotaxanes capable of lithium salt ion‐pair recognition, through triazole based XB and HB interactions in both the axle and the macrocycle components (Figure 1). Binding of Li^+^ switches on the halide anion binding affinity of the rotaxane hosts in a cooperative manner through preorganised axle component‐separated allosteric effects and charge assisted bonding interactions. Importantly, the MIM hosts displayed an enhanced ion pair binding mechanical bond effect when compared to their constituent macrocycles which increases with the number of XB donors in the interlocked host.

## Synthesis of XB and HB rotaxanes

**Scheme 1:**
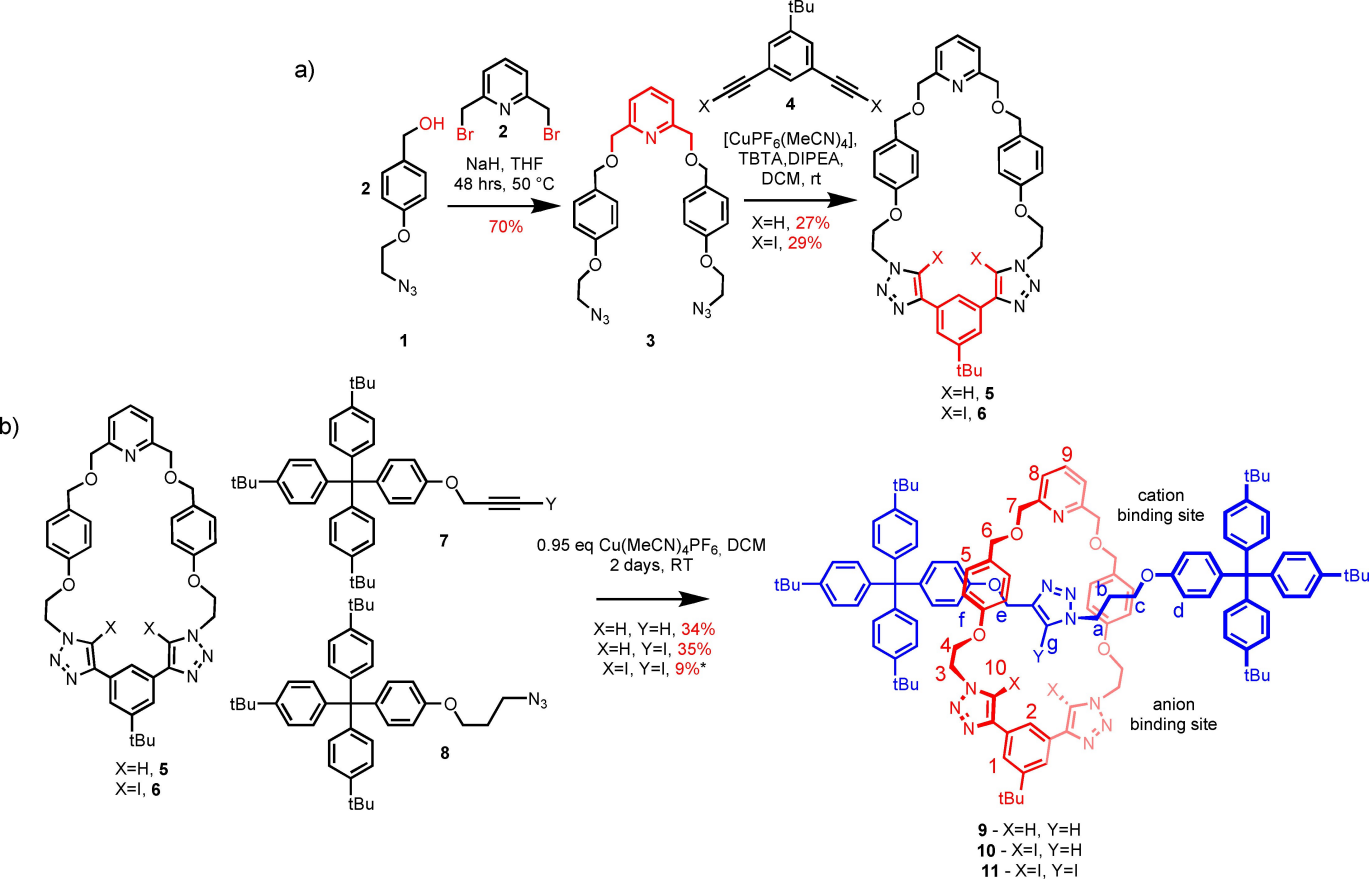
Synthesis of a) macrocycle **5** and **6**, b) heteroditopic rotaxanes **9**, **10**, and **11**.* 3.8 equiv. of Cu(MeCN)_4_PF_6,_ at 30 °C for 5 days.

The heteroditopic macrocycle components, **5** and **6**, of the target rotaxanes were designed to contain a 2,6 bis‐methoxy pyridine motif in the first place to facilitate subsequent active metal template (AMT) MIM synthesis by endotopic copper(I) coordination.^[34]^ In addition, the respective macrocycle's pyridyl motif has the potential to coordinate the lithium cation. The macrocycles were prepared according to the multistep synthetic procedure shown in Scheme 1a. Bis‐bromomethyl pyridine **2** was reacted with two equivalents of azide functionalized benzyl alcohol **1** ^[35]^ in the presence of strong base NaH to form bis‐azide precursor **3** in 70 % yield. Subsequent copper(I)‐catalyzed azide–alkyne cycloaddition (CuAAC) ‘click’ reaction between the bis‐azide **3** and the bis‐alkyne **4** under high dilution conditions in anhydrous DCM afforded the target macrocycles **5** and **6** in 27 % and 29 % yields respectively. (Supporting Information, Section S1.2)

The synthesis of the target heteroditopic [2]rotaxane host systems was achieved via CuAAC active metal template (AMT)^[34]^ synthesis shown in Scheme 1b. To this end, macrocycles **5** and **6** were precomplexed with 0.95 equivalents of [Cu(MeCN)_4_]PF_6_ in CH_2_Cl_2_ and reacted with 5 equivalents of stopper precursors **7**[^34^, ^36^] and **8** ^[34]^ in the presence of catalytic amounts of DIPEA at room temperature for 48 h to afford rotaxanes **9** and **10** in 34 % and 35 % isolated yields respectively. The synthesis of the all XB rotaxane **11** proved much more challenging, presumably due to steric effects by additional XB donor atoms hindering the formation of the interlocked structure. However, by increasing the reaction time to 5 days, temperature of the reaction to 30 °C and adding more [Cu(MeCN)_4_]PF_6_ catalyst to the reaction mixture, rotaxane **11** was successfully isolated following purification in a modest yield of 9 %. Full synthetic details and procedures can be found in the Supporting Information.

All novel structures were characterized by ^1^H NMR and ^13^C NMR spectroscopy, and high‐resolution electrospray ionisation (ESI) mass spectrometry. Evidence for the successful formation of the interlocked structures **9**,**10** and **11** was shown by comparison of ^1^H NMR spectra of the rotaxane product and non‐interlocked macrocycle and axle components. In particular, diagnostic upfield shifts of the macrocyclic hydroquinone protons H_5_ in the interlocked product were indicative of the aromatic donor‐acceptor nature of the electron rich macrocyclic hydroquinone groups and electron deficient triazole axle.

Further evidence for the interlocked nature of the synthesised compounds was provided by 2D ROESY NMR spectroscopy (see Supporting Information, Section S1.3).

## Solid state structure

**Figure 2:**
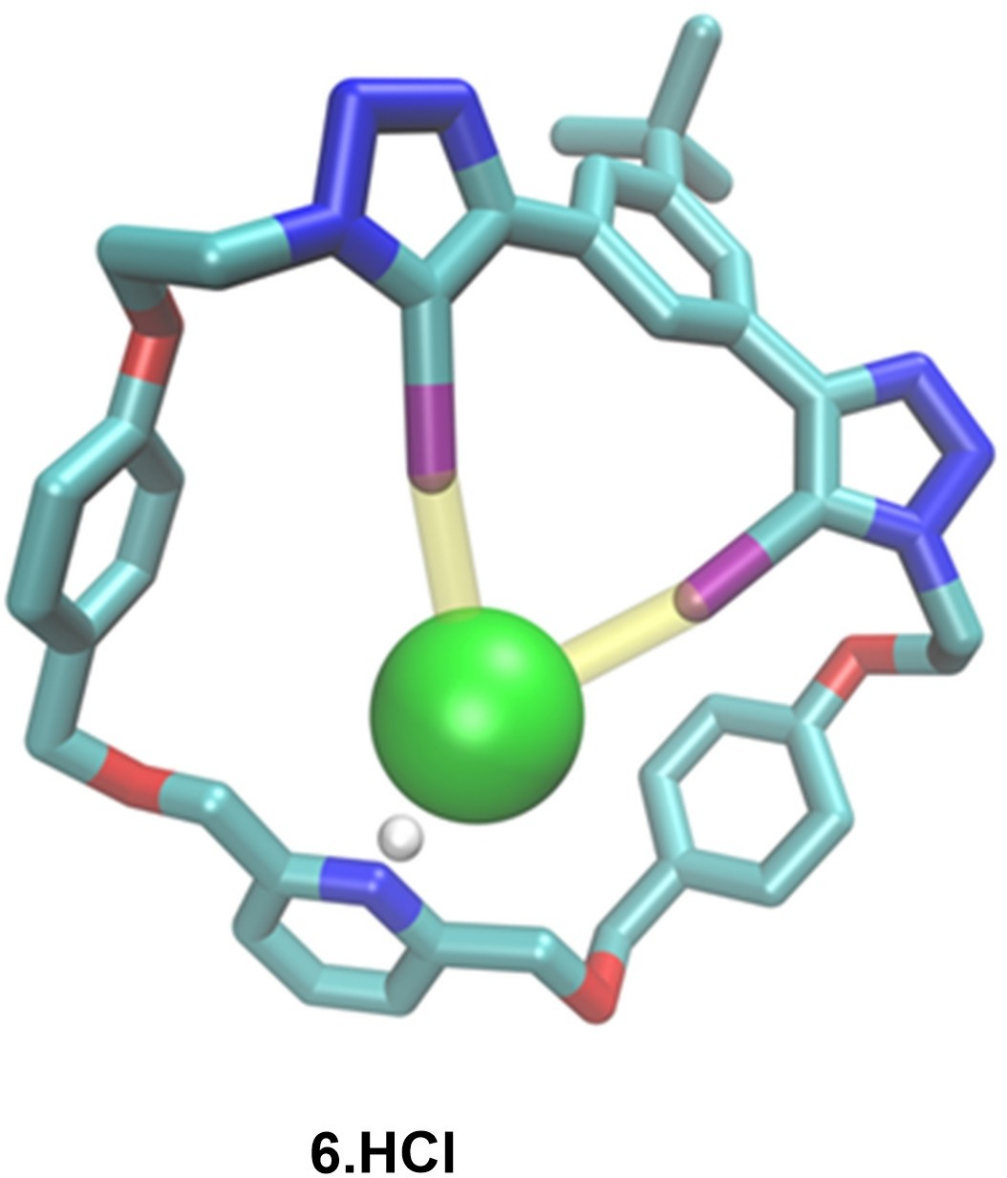
Crystal structure of XB macrocycle hydrochloride **6.HCl**. Colour code of atoms: O‐red, N‐blue, I‐purple, H^+^‐white, Cl^−^‐green, C‐teal. Non‐covalent host‐guest interactions are shown in yellow.

Crystals of the XB macrocycle hydrochloride **6.HCl** suitable for single crystal X‐ray diffraction structural analysis were obtained by slow evaporation of a CDCl_3_ solution of the macrocycle in the presence of excess of TBACl (Figure 2).^[37]^ The solid state structure reveals the two iodo‐triazole based XB donors to exhibit near linear (average 170°) XB interactions with the Cl^−^ anion in a concerted manner. A significantly shorter distance between the XB donors and Cl^−^ compared to their van der Waals radii (average 87 %) is also observed. The macrocycle's pyridyl motif was observed to be protonated, presumably due to trace acid residues in CDCl_3_. The structure thus highlights the respective cation and anion binding sites of the heteroditopic macrocycle.

## Anion and Ion‐pair binding properties of rotaxanes and macrocycles

In order to establish the lithium halide ion‐pair binding mode of the heteroditopic [2]rotaxanes **9**, **10**, **11** and macrocycles **5** and **6**, preliminary qualitative ^1^H NMR ion‐pair complexation experiments were performed. In a typical experiment, the rotaxane was complexed with one equivalent of LiClO_4_ followed by sequential addition of halide anions (as their TBA salts) in CD_3_CN:CDCl_3_ solvent mixtures. This particular solvent combination was chosen based on the solubility of host and potential guest ions.

**Figure 3:**
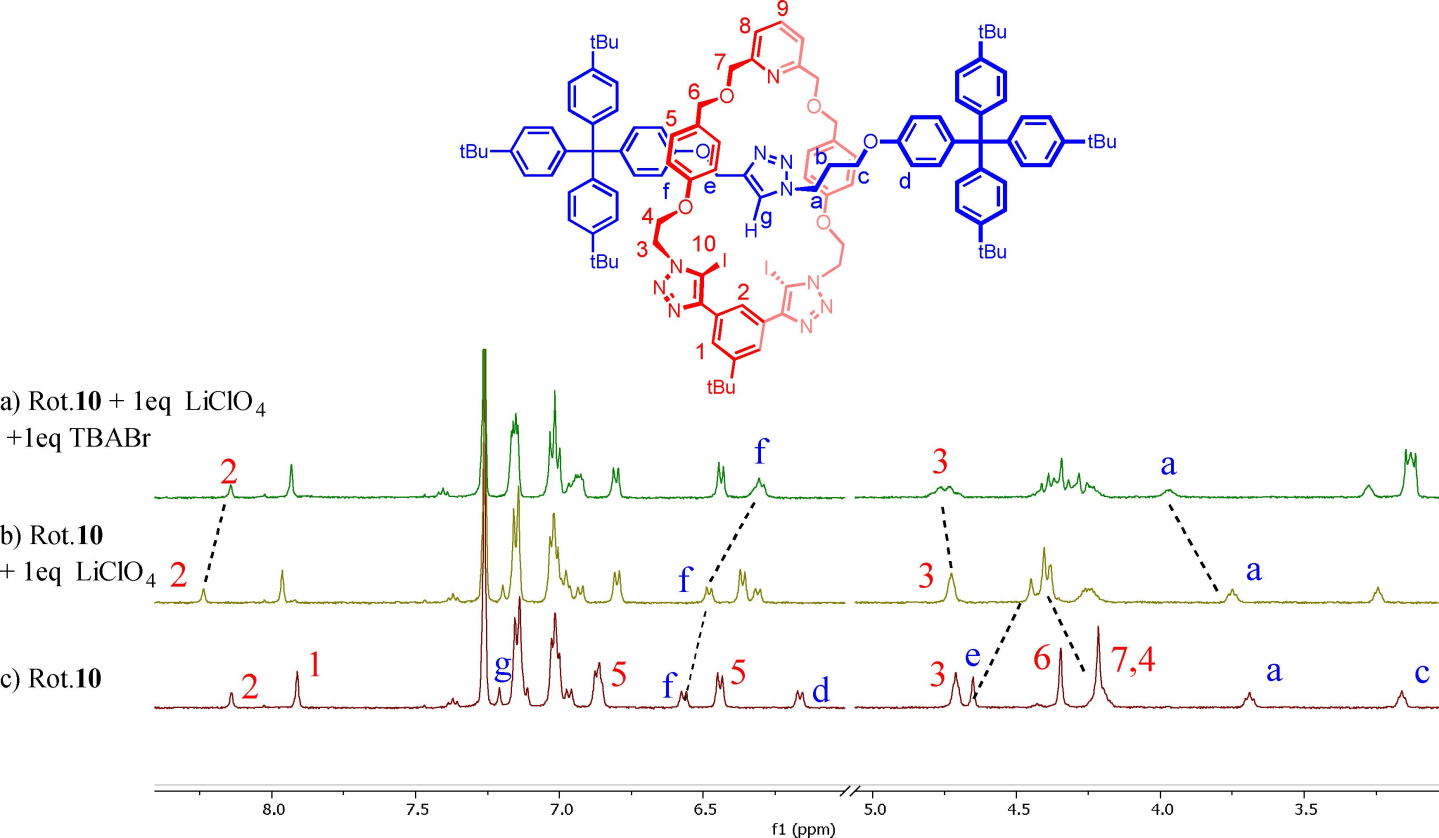
Truncated ^1^H NMR spectra in 1 : 9, CD_3_CN:CDCl_3_, of a)Rotaxane **10** b) Rotaxane **10** in the presence of 1 equivalent of LiClO_4_ c) Rotaxane **10** in the presence of 1 equivalent of LiClO_4_ and TBABr.

All rotaxanes exhibited similar ^1^H NMR perturbations upon the initial addition of the lithium cation and subsequent halide anion (See Supporting Information, Section S3.2 and S4.2). A truncated ^1^H NMR spectrum of rotaxane **10** with the addition of LiClO_4_ and TBABr is shown in Figure 3. Upon addition of LiClO_4,_ significant perturbations of the rotaxane protons were observed in the axle methylene signal H_e_ and terphenyl stopper signal H_f_, as well as the macrocyclic methylene signal H_7_ adjacent to the pyridyl moiety, indicating Li^+^ complexation in the predicted cation binding cavity. Proton H_3_ in the anion binding site did not show any perturbations indicating no coordination of the perchlorate counteranion from the Li salt. Subsequent titration of TBABr caused prominent perturbations to proton signals H_2_, H_3_, H_f_ and H_a_, which are local to the postulated anion binding site. Additionally, all‐HB rotaxane **9** displayed prominent peak perturbations of triazole protons H_10_ and H_g_ upon the addition of anions, further indicating the direct involvement of both macrocycle and axle triazole C−H donors in the anion recognition process (Figure S3.1). While not observed upon titrations with TBABr and TBAI, the addition of TBACl to lithium precomplexed rotaxanes caused decomplexation of the cation in all cases, as evident by the observation of free rotaxane species in ^1^H NMR spectra upon addition of the Cl^−^ salt (Figure S3.1a).

This may be attributed to the strong lattice enthalpy of LiCl driving salt recombination.^[38]^

**Figure 4:**
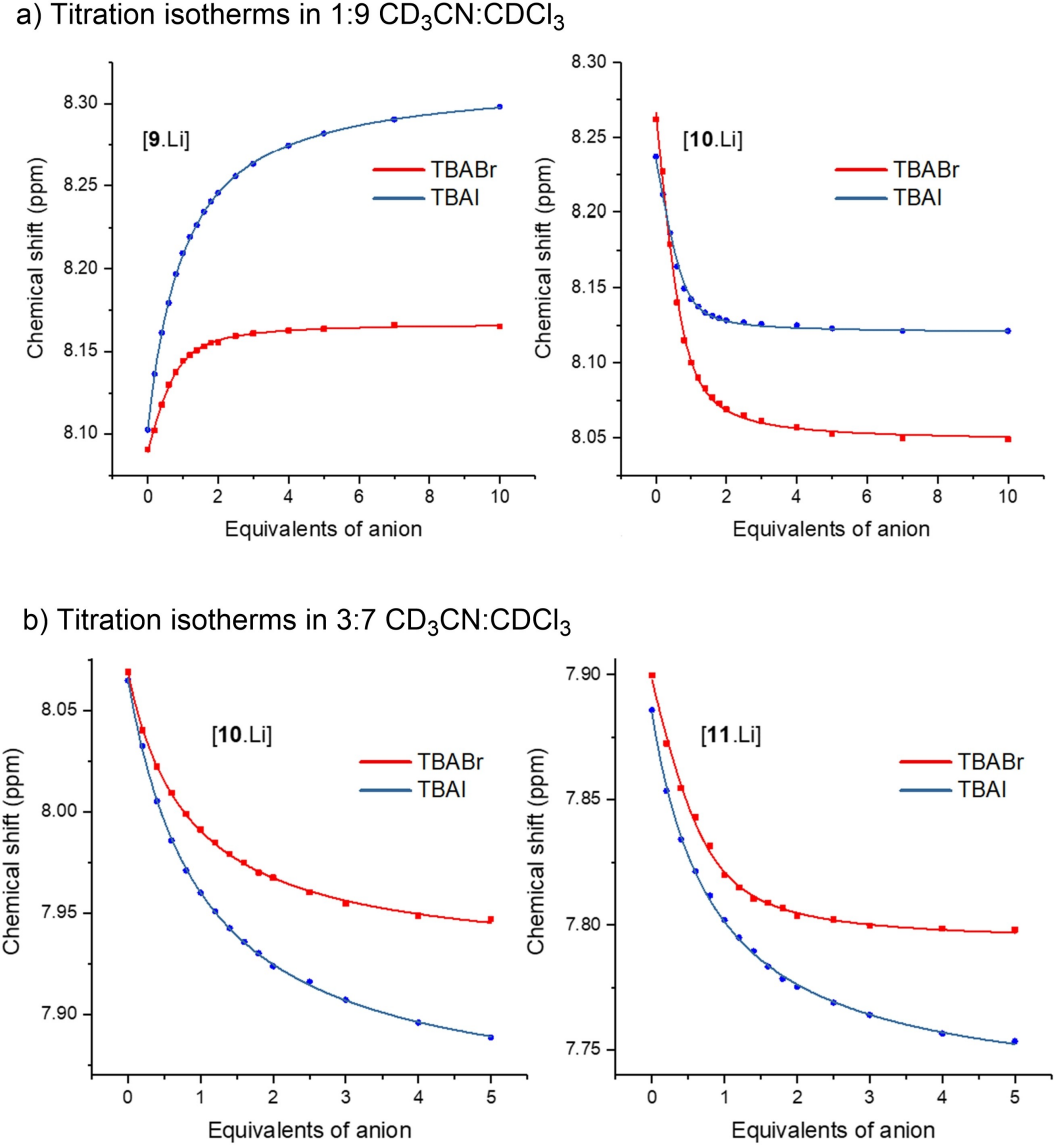
Anion binding isotherms generated by monitoring perturbations of the H_10_ triazole proton signal of lithium precomplexed rotaxane **9** and H_2_ internal tert‐butyl benzene proton signal of lithium precomplexed rotaxane **10** and **11** upon addition of TBABr and TBAI, 298 K, 500 MHz; a) in 1 : 9 CD_3_CN:CDCl_3_ solvent mixture, b) in 3 : 7 CD_3_CN:CDCl_3_ solvent mixture.

Quantitative ion‐pair binding data was determined by monitoring non overlapping ^1^H NMR chemical shifts of rotaxane protons H_2_,H_5_,H_f_ and H_a_,H_10_ in the presence and absence of one equivalent of LiClO_4_ as a function of halide anion concentration. Titration isotherms generated by monitoring protons H_2_ and H_10_ of lithium precomplexed rotaxane with the addition of halide anions are shown in Figure 4. With the lithium complexed rotaxanes, Bindfit^[39]^ analysis determined 1 : 1 stochiometric host‐guest association constants displayed in Table 1.^[40]^ All rotaxanes precomplexed with lithium were found to bind Br^−^ and I^−^ anions in CD_3_CN:CDCl_3_ solvent mixtures. However, no anion binding was observed in the absence of Li^+^. The presence of the bound metal cation in the rotaxane host effectively ‘switches on’ the anion binding capability, due to favourable proximal electrostatic interactions between the two co‐bound ions and the preorganisation of the anion binding site via the mechanical bond (see DFT calculations below).

**Table 1:** Anion association constants (K_a_/M^−1^) for rotaxanes **9**, **10**, **11** and macrocycles **5** and **6** in the presence of 1 equivalent of LiClO_4_ in CD_3_CN:CDCl_3_ solvent mixtures.^[a]^

Anion	Cation	M.**5**	M.**6**	R.**9**	R.**10**	R.**10**	R.**11**
		1 : 9 CD_3_CN:CDCl_3_				3 : 7 CD_3_CN:CDCl_3_	
Cl^−^	Li^+^	–	–	^[b]^	^[b]^	–	^[b]^
Br^−^	Li^+^	^[b]^	^[b]^	6946(9)	>10^4^	5666(7)	>10^4^
I^−^	Li^+^	^[b]^	^[b]^	2369(7)	>10^4^	2890(5)	3419(7)

[a] K_a_ values were calculated using the global fit option in Bindfit software using 1 : 1 binding model. Errors (%) are in parenthesis. Li^+^ is added as LiClO_4_ and all anions as their TBA salts [Receptor]=1 mM, *T*=298 K. ^[^b] Salt recombination

Interestingly, Table 1 highlights the increased strength of halide anion binding in these interlocked hosts as the number of XB sigma‐hole donors in the cavity increase. In 1 : 9 CD_3_CN:CDCl_3_ mixtures, a significant increase in the magnitudes of association constants of LiBr and LiI is observed for the mixed XB‐HB rotaxane **10** compared to its all HB rotaxane analogue **9**. As the K_a_ values for **10** were >10^4^ M^−1^, a more competitive solvent mixture system of 3 : 7 CD_3_CN : CDCl_3_ was used to obtain K_a_ values for rotaxanes **10** and **11**. Replacement of the axle's single triazole HB bond donor of **10** with an XB bond donor in **11** resulted in a large enhancement of halide anion binding strength, particularly with the smaller Br^−^ anion. All the rotaxane hosts showed a two‐ to three‐fold selectivity for the LiBr ion‐pair over LiI. This may be attributed to the relative higher basicity of Br^−^ and the size complementarity of LiBr to the preorganised ion‐pair binding cavities of the rotaxanes.

To further investigate the selectivity of rotaxane **10** for anions, analogous ^1^H NMR titrations were performed for the lithium precomplexed rotaxane (**10.Li**) with the sequential addition of AcO^−^, NO_3_^−^ and SCN^−^ anions. In all instances, Li^+^ decomplexation was observed upon addition of the respective anion (Figure S3.3), even though the lattice energies of LiNO_3_ and LiSCN are lower than the lattice energy of LiBr.[^38^, ^41^] Hence, the selectivity presumably stems from the preferential halide binding properties of rotaxane **10**, specifically the binding of the softer Br^−^ and I^−^ anions to XB donors^[42]^ and size complementarity of halide anions to the anion binding site.

Attention then turned to investigate the selectivity of rotaxane **10** for group I metal cations. Due to poor solubility of the receptor in the presence of NaClO_4_, NaBArF was used as the Na^+^ salt. ^1^H NMR titration experiments in 3 : 7 CD_3_CN:CDCl_3_ determined the 1 : 1 stoichiometric association constant for Li^+^ (K_a_=697(1) M^−1^) was more than three times higher than the association constant value for Na^+^ (K_a_=181(1) M^−1^) (See Supporting Information, Section S3.3). This may be attributed to size complementarity and higher charge density of Li^+^ cation.

In contrast to the rotaxanes, HB macrocycle **5** and XB macrocycle **6** did not bind any LiX ion pairs in 1 : 9 CD_3_CN:CDCl_3_. Upon addition of LiClO_4_, the proton signals adjacent to the respective macrocycle's pyridyl moiety, H_6_ and H_7_ shifted downfield, and H_8_ shifted upfield. This suggests complexation of Li^+^ in the proximity of the pyridyl cation binding site, as observed in the solid state structure (Figure 2). Upon subsequent addition of TBABr or TBAI, these signals reverted back to their original metal cation free chemical shift values, suggesting potential ion‐pair formation outside of the respective macrocycle, preventing effective ion‐pair recognition (Supporting Information, Section S3.2).

## DFT calculations

Density functional theory (DFT) studies were performed to investigate the binding modes of lithium halides to rotaxanes **9**,**10**,**11** and macrocycles **5**,**6**. Simulations were carried out in the gas phase using Gaussian 16^[43]^ at the B3LYP level of theory^[44]^ using aug‐cc‐pVTZ basis set for chlorine atoms, aug‐cc‐pVTZ‐PP basis set for larger halide atoms, and 6–31 g* basis for other atoms.[^45^, ^46^, ^47^] Counterpoise correction was invoked to correct for the basis set superposition error.[^48^, ^49^] The initial input structures were generated from modifying solid state crystal structures of macrocycle **6** and of the HB triazole axle.^[50]^ The terminal terphenyl stoppers of the axle were replaced by methyl groups for ease of simulation.

**Figure 5:**
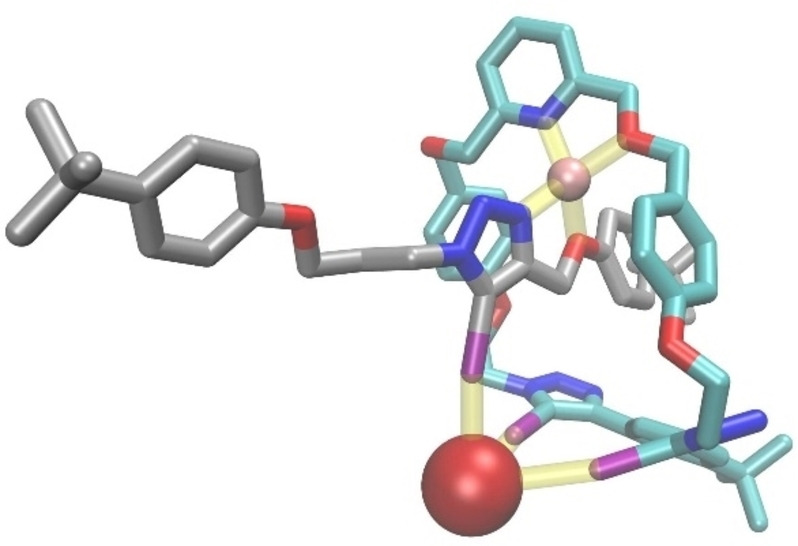
DFT optimised structure of LiBr bound rotaxane **10**. H atoms are omitted for clarity. Colour code of atoms: O‐red, N‐blue, I‐purple, Li‐pink, Br‐ brown, C_(axle)_ ‐gray C_(MC)_‐teal. Non‐covalent host‐guest interactions are shown in yellow.

A geometry optimised structure of LiBr bound rotaxane **11** is shown in Figure 5. According to the optimised structure, Li^+^ interacts with the pyridine nitrogen and the adjacent oxygen of the macrocycle, and the triazole nitrogen and the neighbouring oxygen of the axle, in a four‐coordination geometry. The bromide anion interacts with all three triazole XB bonds of both the axle and the macrocycle, forming characteristic near linear interactions (168^0^–172^0^). The average C−I‐X^−^ bond length of bromide bound rotaxanes **10** and **11** is 82.5 % of the sum of the bromide ionic radius and the iodine van der Waals radius. The corresponding value for iodide bound rotaxanes **10** and **11** is 85 %, which is in line with the experimental evidence of stronger halogen bonding for bromide bound rotaxanes. The optimised structures agree with the corresponding solution ^1^H NMR titration data where significant proton perturbations were observed in the postulated anion and cation binding sites (Supporting Information, section S4.2).

Similarly, DFT calculations of HB macrocycle **5** and XB macrocycle **6** show that Li^+^ interacts with pyridine nitrogen and the adjacent oxygen atoms (Supporting Information, Section S4.1). This is confirmed by solution ^1^H NMR titration data where peaks H_8_ and H_7_ of the macrocycle are shifted significantly, upon addition of the cation (Supporting Information, Section S4.2).

Calculated lithium halide complexation energies for all rotaxanes are presented in Table S4. Aligning with ^1^H NMR titration results, complexation energies of the rotaxanes were far superior compared to their analogous macrocycles. For all rotaxanes LiBr showed a slight increase of complexation energy compared to LiI. It is however important to note that solvophobic effects from XB donors are not accounted for in gas phase calculations. Therefore, it is difficult to compare the small changes in association constants of rotaxane **9**, **10**, **11**.

The second sphere electrostatic effect of Li^+^ for cooperative binding of anions is reflected in the distribution of electrostatic potential mapped onto the electron density surface. The isoelectric maps for rotaxanes **10** and **11** are presented in Supporting Information, Section S4.4. Binding of Li^+^ increases the potential energy of the proximal XB/HB donor atom of the axle. This is evident from the increase of potential energy surrounding the axle iodine atom of rotaxane **11** from 245 kcal mol^−1^ to 301 kcal mol^−1^, and axle H atom of rotaxane **10** from 195 kcal mol^−1^ to 257 kcal mol^−1^ when Li^+^ is bound.

## Conclusions

A family of XB and HB heteroditopic [2]rotaxane hosts with axle separated binding cavities for simultaneous cation and anion binding were synthesised for lithium halide ion pair recognition. ^1^H NMR titration studies and DFT calculations revealed the interlocked rotaxane hosts to show superior ion‐pair binding for lithium halides compared to their non‐interlocked macrocycle components. Binding of Li^+^ was found to both polarise the XB donors and preorganise the anion binding site, leading to a cation‐induced ‘switch on’ of cooperative halide recognition. Quantitative ion‐pair binding data revealed all rotaxanes to display notable selectivity for LiBr over LiI. In addition, the strength of bromide and iodide halide binding was demonstrated to increase significantly with increasing number of XB donors in the interlocked rotaxane cavity, making all XB rotaxane **11** the strongest ion‐pair binding rotaxane of the family. Importantly, these results serve to highlight how the unique cooperative rotaxane axle‐macrocycle component mechanical bond effect, in combination with powerful XB sigma‐hole interactions, creates highly effective heteroditopic ion‐pair host systems. Exploiting mechanical bond design for MIM heteroditopic construction as an efficient strategy for ion‐pair recognition is continuing in our laboratories.

## Conflict of interest

The authors declare no conflict of interest.

1

## Supporting information

As a service to our authors and readers, this journal provides supporting information supplied by the authors. Such materials are peer reviewed and may be re‐organized for online delivery, but are not copy‐edited or typeset. Technical support issues arising from supporting information (other than missing files) should be addressed to the authors.

Supporting Information

## Data Availability

The data that support the findings of this study are available in the supplementary material of this article.
