# Supplementary material for: Mechanical Bond Enhanced Lithium Halide Ion‐Pair Binding by Halogen Bonding Heteroditopic Rotaxanes
Source: Chemistry. 2022 Jul 6;28(48):e202201209. doi: 10.1002/chem.202201209 (PMC9541756; doi:10.1002/chem.202201209)
Supplement: Supplementary file 1 — Supporting Information [file CHEM-28-0-s001.pdf]

Chemistry–A European Journal

Supporting Information

Mechanical Bond Enhanced Lithium Halide Ion-Pair Binding by Halogen Bonding Heteroditopic Rotaxanes

Vihanga K. Munasinghe, Jessica Pancholi, Dilhan Manawadu, Zongyao Zhang, and Paul D. Beer*

Table of Contents

S1. Synthesis of compounds 2
S1.1. General Information 2
S1.2. Synthetic procedures and characterisation 2
Synthesis of macrocycle **5** and **6** 2
Synthesis of rotaxane **9**, **10** and **11** 5
S1.3. Spectral characterisation of novel precursors 9
S2. Solid State Structures 26
S3. ^{1}H NMR binding studies 28
S3.1. General Procedure 28
S3.2. Ion-pair binding ^{1}H NMR titration experiments 29
^{1}H NMR titrations of rotaxane **9** 29
^{1}H NMR titrations of rotaxane **10** 31
^{1}H NMR titrations of rotaxane **11** 36
^{1}H NMR titrations of macrocycle **6** 38
^{1}H NMR titrations of macrocycle **5** 39
S3.3. Cation binding ^{1}H NMR titration experiments 40
S4. DFT calculations 42
S4.1. DFT optimised structures 43
S4.2. Comparison of optimised structures with ^{1}H NMR data 45
S4.3. Calculated association constants 49
S4.4. Electrostatic potential map 50
S5. References 50

S1. Synthesis of compounds

S1.1. General Information

All solvents and reagents were purchased from commercial suppliers and used as received unless otherwise stated. Dry solvents were obtained by purging with nitrogen and then passing through an MBraun MPSP-800 column. H_2O was de-ionized and micro filtered using a Milli-Q® Millipore machine. Column chromatography was carried out on Merck® silica gel 60 under a positive pressure of nitrogen. Routine NMR spectra were recorded on either a Varian Mercury 300, a Bruker AVIII 400 or a Bruker AVIII 500 spectrometer with ^1H NMR titrations recorded on a Bruker AVIII 500 spectrometer. TBA salts were stored in a vacuum desiccator containing phosphorus pentoxide prior to use. Where mixtures of solvents were used, ratios are reported by volume. Chemical shifts are quoted in parts per million relative to the residual solvent peak. Mass spectra were recorded on a Bruker µTOF spectrometer.

S1.2. Synthetic procedures and characterisation

Synthesis of macrocycle **5** and **6**

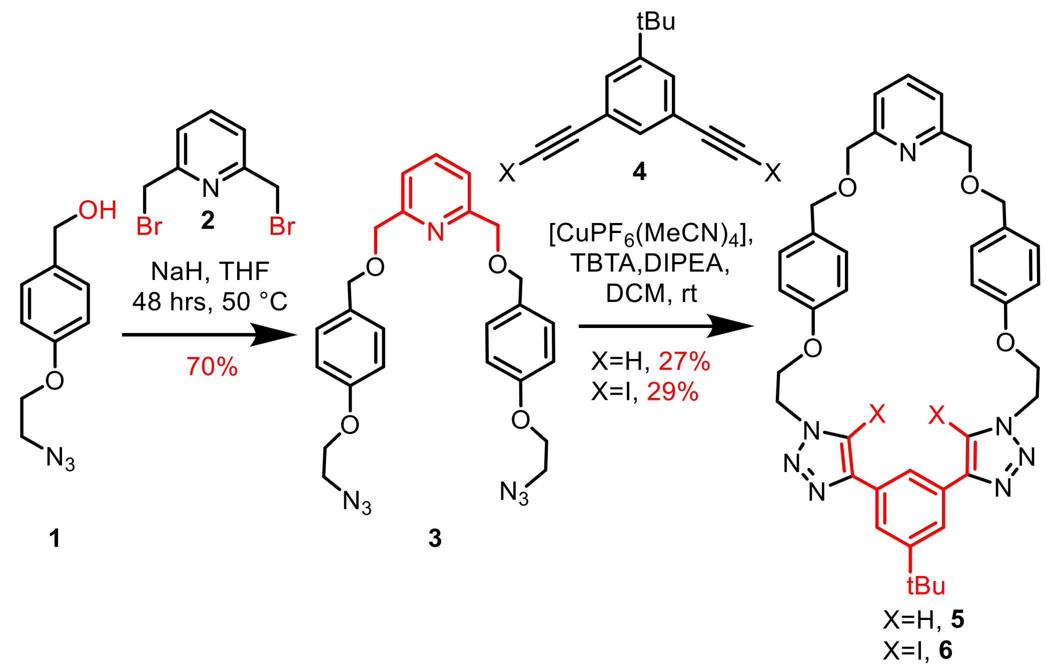

*Scheme S1.1 Synthesis of macrocycle **5** and **6***

Compound **1**,**2** and **4** were synthesized according to literature procedures.[1–3]

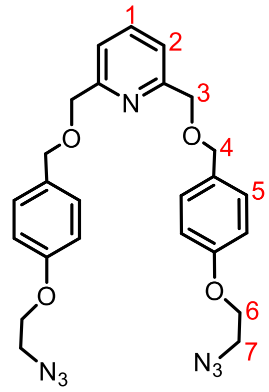

3

Compound 3: The alcohol precursor **1** (1.50g,7.76 mmol) was dissolved in 50 ml of dry THF. NaH (0.295 g of a 60% dispersion in mineral oil, 7.37 mmol) was added to the reaction mixture and was stirred at room temperature under N_2 for 20 minutes. Subsequently, the bis bromo pyridine precursor **2** was added and was stirred for a further 60 mins and then was heated at 50 °C for 48 hours. Solvent was then removed *in vacuo*. The residue was redissolved in DCM (50 ml), washed with brine (50 ml x 2), dried over $MgSO_4$ and solvent was removed *in vacuo*. The crude product was purified by silica gel column chromatography (0-2% MeOH in DCM) to afford the macrocycle precursor **3** as a white solid (1.14 g, 70%).

^{1}H NMR (500 MHz, $CDCl_3$) δ 7.58 (t, *J* = 7.7 Hz, 1H, H_1), 7.27 (d, *J* = 7.7 Hz, 2H, H_2), 7.20 (d, *J* = 8.5 Hz, 4H, H_4), 6.78 (d, *J* = 8.5 Hz, 4H, H_3), 4.53 (s, 4H, H_5), 4.46 (s, 4H, H_5), 3.99 (t, *J* = 5.0 Hz, 4H, H_6), 3.43 (t, *J* = 5.0 Hz, 4H, H_7)

^{13}C NMR (126 MHz, $CDCl_3$) δ 157.87, 137.15, 130.79, 129.43, 119.91, 114.46, 72.79, 72.39, 66.91, 50.02.

HRMS (ESI +ve) m/z: 490.2197 ($[M+H]^+$, $C_{25}H_{28}N_7O_4$ requires 490.2197)

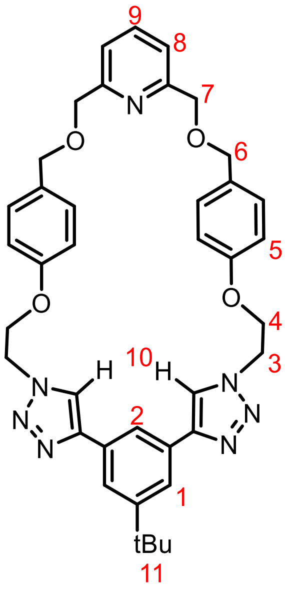

5

Macrocycle 5 : Bis azide precursor **3** (250 mg, 0.51 mmol), [$CuPF_6(MeCN)_4$] (190 mg, 0.51 mmol) and TBTA (271 mg, 0.51 mmol) were dissolved in dry DCM (200 mL) and stirred vigorously for 10 minutes. Bisprotoalkyne **4** (93 mg, 0.51 mmol) and DIPEA (0.36 mL, 2.04 mmol) were added sequentially, the flask wrapped in foil and stirred at room temperature for 48 h. The solvent volume was halved *in vacuo*, and washed with basic EDTA/$NH_4OH_{(aq)}$ (30 mL x 3) and H_2O (20 mL), dried over $MgSO_4$ and solvent evaporated *in vacuo*. The crude product was purified by silica gel column chromatography (10-50% EtOAc in DCM) to yield XB macrocycle as a gold foam (92.5 mg, 27%).

^{1}H NMR (400 MHz, $CDCl_3$) δ 8.00 (s, 2H, H_{10}), 7.95 (d, J = 1.6 Hz, 2H, H_1), 7.83 (t, J = 1.6 Hz, 1H, H_2), 7.68 (broad s, 1H, H_9), 7.33 (d, J = 8.0 Hz, 2H, H_8), 7.23 (d, J = 8.0 Hz, 4H, H_5), 6.80 (d, J = 8.0 Hz, 4H, H_5), 4.81 (m, 4H, H_4), 4.57 (s, 4H, H_6), 4.43 (s, 4H, H_7), 4.36 (m, 4H, H_3), 1.41 (s, 9H, H_{11}).

^{13}C NMR (126 MHz, $CDCl_3$) δ 157,67, 157.75, 152.73, 148.21, 137.25, 131.15, 130.10, 122.98, 121.21, 120.79, 120.38, 114.70, 71.38, 72.12, 66.93, 50.13, 35.13, 31.49.

HRMS (ESI +ve) m/z: 672.3276 ($[M+H]^+$, $C_{39}H_{42}N_7O_4$ requires 672.3293)

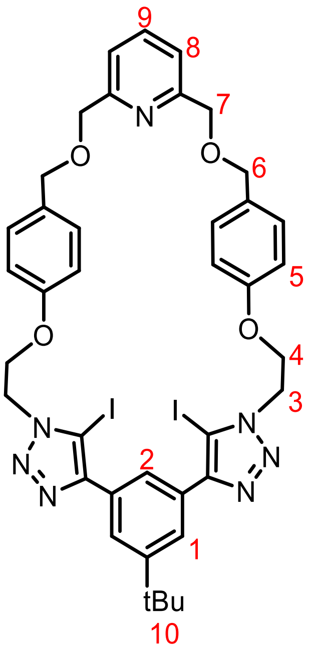

6

Macrocycle 6 : The XB macrocycle **6** was synthesized following the same procedure used to synthesize macrocycle **5**, using bis azide precursor **3** (128 mg, 0.26 mmol), [$CuPF_6(MeCN)_4$] (97.5 mg, 0.26 mmol) and TBTA (139 mg, 0.26 mmol), bis iodo alkyne precursor **4** (114 mg, 0.26 mmol), DIPEA (0.18 mL, 1.04 mmol) in dry degassed DCM (100 mL). Crude mixture was purified by preparative thin layer chromatography (SiO_2, 0-2% MeOH in DCM) to yield the desired rotaxane as a gold form (65.2 mg, 27%).

^{1}H NMR (500 MHz, $(CD_3)_2CO$) δ 8.32 (t, J = 1.6 Hz, 1H, H_2), 8.03 (d, J = 1.6 Hz, 2H, H_1), 7.73 (t, J = 7.8 Hz, 1H, H_9), 7.34 (d, J = 7.7 Hz, 2H, H_8), 7.24 (d, J = 10.0 Hz, 4H, H_5), 6.87 (d, J = 10.0 Hz, 4H, H_5), 4.97 (m, 4H, H_4), 4.58 – 4.53 (m, 8H, H_3 & H_6), 4.34 (s, 4H, H_7), 1.44 (s, 9H, H_{10}).

^{13}C NMR (126 MHz, $(CD_3)_2CO$) δ 157.98, 157.93, 151.55, 149.43, 136.88, 131.05, 130.82, 129.58, 124.50, 123.72, 119.80, 114.35, 71.48, 71.31, 66.83, 49.88, 34.82, 30.78.

HRMS (ESI +ve) m/z: 924.1212 ($[M+H]^+$, $C_{39}H_{40}N_7O_4I_2$ requires 924.1226)

Synthesis of rotaxane **9, 10** and **11**

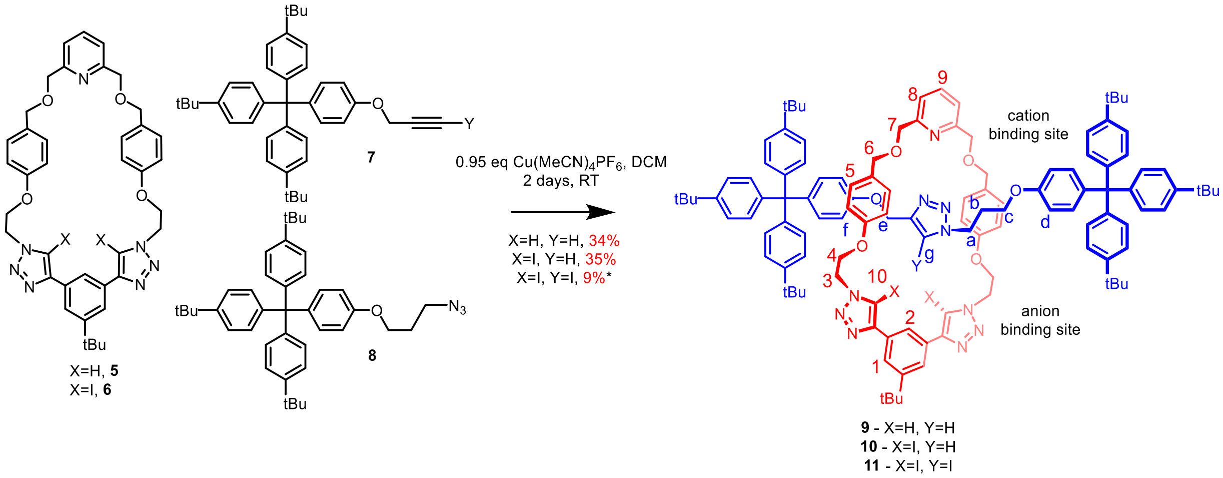

*Scheme S1.2: Synthesis of Rotaxanes **9**, **10** and **11**. *3.8 eq. of $Cu(MeCN)_4PF_6$, at 30°C for 5 days.*

Axle precursors **7** and **8** were synthesised according to literature procedures.[4,5]

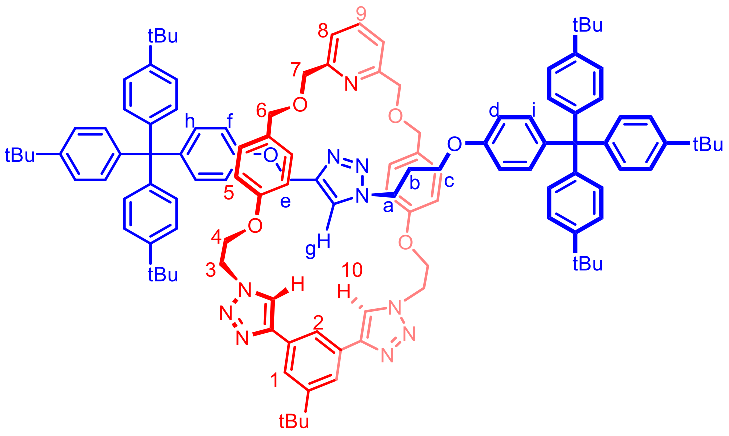

9

Rotaxane 9: Macrocycle **5** (10 mg, 0.015 mmol) and [$CuPF_6(MeCN)_4$] (5.27 mg, 0.0142 mmol) were dissolved in 0.75 ml dry degassed DCM and was stirred for 30 minutes. A solution of stopper azide **7** (44.0 mg, 0.075 mmol) and stopper alkyne **8** (40.4 mg, 0.075 mmol) in dry degassed DCM (0.75 ml) was added to the Cu-complexed macrocycle solution, followed by 4-5 drops of DIPEA. The reaction mixture was sealed and the homogenous solution was stirred for 48 hours. Subsequently, the reaction mixture was diluted with 10 ml DCM, washed with basic EDTA/$NH_4OH_{(aq)}$ (10 ml x 3) and H_2O (10 ml x 2), dried over $MgSO_4$ and was concentrated in a rotary evaporator. The crude product was purified by preparative thin layer chromatography (SiO_2, 0-2% MeOH in DCM) to yield the desired rotaxane as a white solid (9.1 mg, 34%).

^{1}H NMR (500 MHz, $(CD_3)_2CO$) δ 8.36 (s, 2H, H_{10}), 8.02 (d, *J* = 1.5 Hz, 2H, H_1), 7.98 (t, *J* = 1.5 Hz, 1H, H_2), 7.59 (t, *J* = 7.7 Hz, 1H, H_9), 7.48 (s, 1H, H_g), 7.31 – 7.27 (m, 16H, H_8 & $H_{\text{stopper Ar}\boldsymbol{H}}$), 7.11 – 7.07 (m, 14H, $H_{\text{stopper Ar}\boldsymbol{H}}$), 7.00 (m, 6H, H_5&H_h), 6.92 (d, *J* = 10 Hz, 2H, H_i), 6.73 (d, *J* = 10 Hz, 2H, H_f), 6.59 (d, *J* = 10 Hz, 4H, H_5), 6.34 (d, *J* = 10 Hz, 2H, H_d), 4.84 (s, 2H, H_e), 4.78 (m, 4H, H_3), 4.44 (s, 4H, H_6), 4.33 (m, 4H, H_4), 4.28 (s, 4H, H_7), 3.84 (t, *J* = 7.1 Hz, 2H, H_a), 3.32 (t, *J* = 5.9 Hz, 2H, H_c), 1.63 (m, 2H, H_b), 1.30 (m, 63H, $H_{t\text{-Bu}}$).

^{13}C NMR (126 MHz, $(CD_3)_2CO$) δ 158.77, 157.23, 152.93, 149.09, 149.05, 148.17, 145.35, 145.26, 144.02, 140.47, 140.18, 137.87, 132.73, 132.53, 131.49, 131.40, 130.50, 125.10, 124.81, 122.79, 122.27, 121.32, 120.89, 115.34, 114.27, 113.95, 72.43, 72.23, 67.42, 64.66, 63.88, 61.94, 54.97, 50.43, 47.26, 35.51, 34.87, 32.64, 31.73, 31.68, 23.33, 14.36, 1.40.

HRMS: (ESI +ve) m/z: 1803.0628 ($[M+H]^+$, $C_{119}H_{137}N_{10}O_6$ requires 1803.0749)

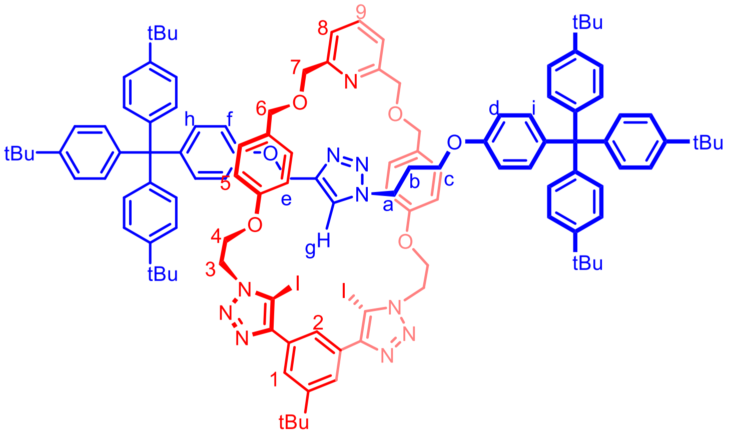

10

Rotaxane 10: The rotaxane was synthesized following the same procedure used to synthesize rotaxane **9**, using the XB macrocycle **6** (10 mg, 0.01 mmol), [$CuPF_6(MeCN)_4$] (3.97 mg, 0.0095 mmol), stopper azide **7** (31.8 mg , 0.05 mmol), stopper alkyne **8** (29.4 mg, 0.05 mmol) in dry degassed DCM (1.1 ml total volume). The crude mixture was purified by preparative thin layer chromatography (SiO_2, 0-2% MeOH in DCM) to yield the desired rotaxane as a white solid (7.78 mg, 35%).

^{1}H NMR (400 MHz, $CDCl_3$) δ 8.23 (t, *J* = 1.6 Hz, 1H, H_2), 7.99 (d, *J* = 1.6 Hz, 2H, H_1), 7.43 (t, *J* = 7.7 Hz, 1H, H_9), 7.23 – 7.17 (m, 17H, $H_{stopper\ ArH}$,H_g ,H_8), 7.09 – 7.02 (m, 16H, H_h , $H_{stopper\ ArH}$), 6.97 – 6.89 (m, 6H, H_5 & H_i), 6.65 (d, *J* = 7.7 Hz, 2H, H_f), 6.52 (d, *J* = 7.7 Hz, 4H, H_5), 6.24 (d, *J* = 7.7 Hz, 2H, H_d), 4.80-4.71 (m, 6H, H_e,H_3), 4.42 (s, 4H, H_6), 4.34 – 4.18 (m, 8H, H_4, H_7), 3.73 (t, *J* = 7.4 Hz, 2H, H_a), 3.23 (t, *J* = 5.7 Hz, 2H, H_c), 1.29 (s, 63H, $H_{t\text{-}Bu}$).

^{13}C NMR (126 MHz, $(CD_3)_2CO$) δ 158.76, 158.65, 157.30, 157.14, 152.41, 150.25, 149.17, 148.99, 145.39, 145.32, 143.90, 140.28, 139.97, 137.76, 132.69, 132.54, 131.84, 131.45, 131.35, 130.40, 125.29, 125.14, 124.62, 124.52, 120.65, 115.23, 114.29, 113.93, 80.49, 72.49, 72.20, 67.61, 64.69, 63.90, 62.06, 50.79, 47.33, 35.71, 34.87, 32.63, 31.73, 31.68, 23.32, 14.35, 1.40.

HRMS: (ESI +ve) m/z: 2054.8544 ($[M+H]^+$, $C_{119}H_{136}N_{10}O_6I$ requires 2054.8682)

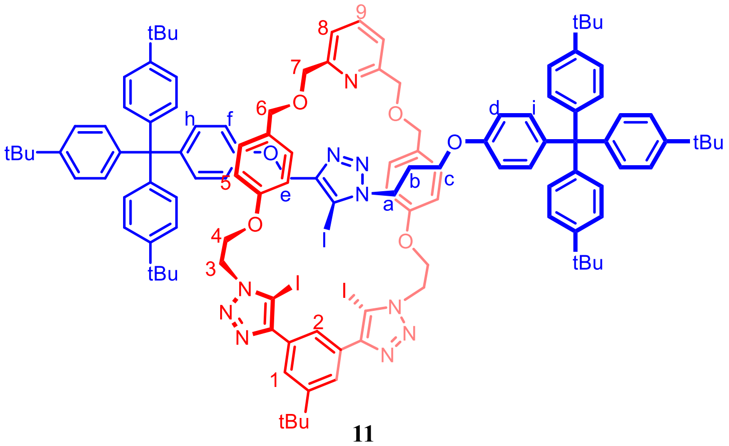

11

Rotaxane 11: Macrocycle **6** (10 mg, 0.01 mmol) and [$CuPF_6(MeCN)_4$] (3.97 mg, 0.0095 mmol) were dissolved in 0.55 ml dry degassed DCM and was stirred for 30 minutes. A solution of stopper azide **7** (31.8 mg , 0.05 mmol), stopper alkyne **8** (29.4 mg, 0.05 mmol) in dry degassed DCM (0.55 ml) was added to the Cu-complexed macrocycle solution. Reaction mixture was sealed and heated to 30 °C. Subsequently, [$CuPF_6(MeCN)_4$] (11.91 mg, 0.0285 mmol) was added portion wise after 48 hours, 72 hours and 96 hours. After 5 days, the reaction mixture was diluted with 10 ml DCM, washed with basic EDTA/$NH_4OH_{(aq)}$ (10 ml x 3) and H_2O (10 ml x 2), dried over $MgSO_4$ and was concentrated in a rotary evaporator. The crude product was purified by preparative thin layer chromatography (SiO_2, 0-2% MeOH in DCM) to yield the desired rotaxane as a white solid (2.00 mg, 8.5%)

^{1}H NMR (500 MHz, $(CD_3)_2CO$) δ 8.17 (t, *J* = 1.7 Hz, 1H, H_2), 8.02 (d, *J* = 1.7 Hz, 2H, H_1), 7.51 (t, *J* = 7.8 Hz, 1H, H_9), 7.34 – 7.26 (m, 14H, $H_{stopper\ ArH}$), 7.23 (d, *J* =8.0 Hz, 2H, H_8), 7.18 – 7.08 (m, 14H, $H_{stopper\ ArH}$), 7.06 (d, *J* =8.8 Hz, 2H, H_h), 7.00 (d, *J* = 8.6 Hz, 4H, H_5), 6.89 (d, *J* = 8.8 Hz, 2H, H_i), 6.77 (d, *J* = 8.8 Hz, 2H, H_f), 6.60 (d, *J* = 8.8 Hz, 4H, H_5), 6.21 (d, *J* = 8.8 Hz, 2H, H_d), 4.92 (t, *J* = 4.7 Hz, 4H, H_3), 4.74 (s, 2H, H_e), 4.46 (d, *J* = 2.5 Hz, 4H, H_6), 4.37 (dt, *J* = 13.1, 4.9 Hz, 4H, H_4), 4.23 (s, 4H, H_7), 3.87 (t, *J* = 7.1 Hz, 2H, H_a), 3.22 (t, *J* = 5.7 Hz, 2H, H_c), 1.59 (d, *J* = 6.7 Hz, 2H, H_b), 1.30 (m, 63H, H_{t-Bu}).

^{13}C NMR (126 MHz, $(CD_3)_2CO$) δ 157.95, 157.80, 156.53, 156.28, 151.37, 149.35, 148.22, 148.13, 146.64, 144.56, 144.46, 139.64, 138.98, 136.75, 131.78, 131.65, 130.96, 130.62, 130.57, 129.59, 124.35, 124.30, 124.21, 123.90, 119.31, 114.31, 113.58, 113.09, 81.83, 71.63, 71.15, 66.77, 63.56, 63.06, 61.37, 50.00, 46.90, 34.85, 33.99, 31.74, 30.79, 22.43, 13.45, 0.50.

HRMS (ESI +ve) m/z: 2180.7568 ($[M+H]^+$, $C_{119}H_{134}N_{10}O_6I_3$ requires 2180.7649)

S1.3. Spectral characterisation of novel precursors

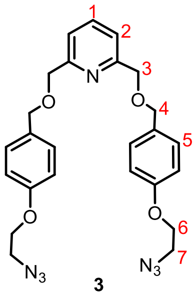

3

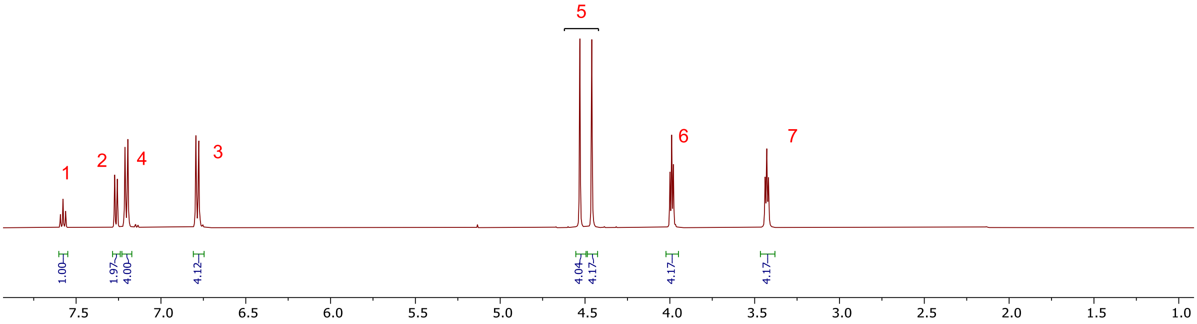

*Figure S1.1: ^{1}H NMR spectrum of macrocycle precursor **3** (500 MHz, 298 K, $CDCl_3$).*

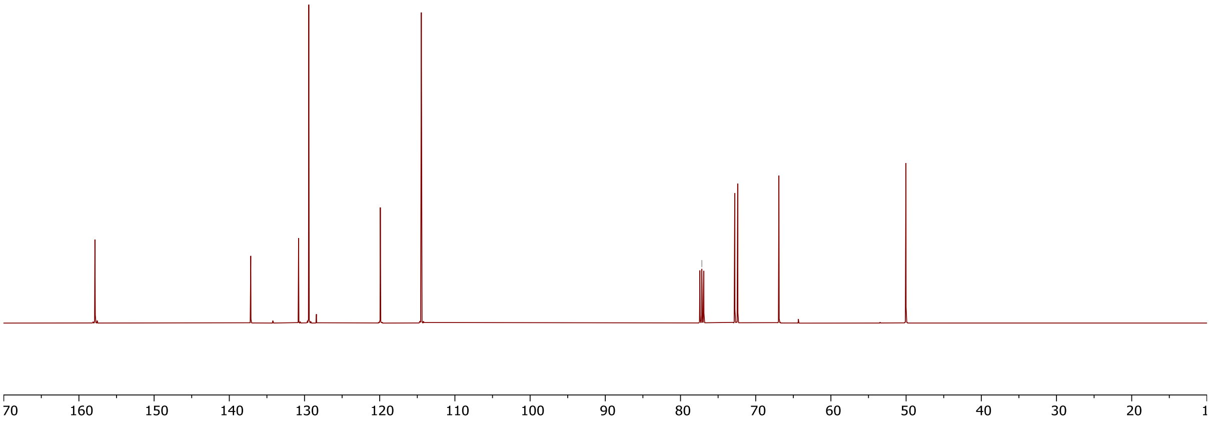

*Figure S1.2: ^{13}C NMR spectrum of macrocycle precursor **3** (126 MHz, 298 K, $CDCl_3$).*

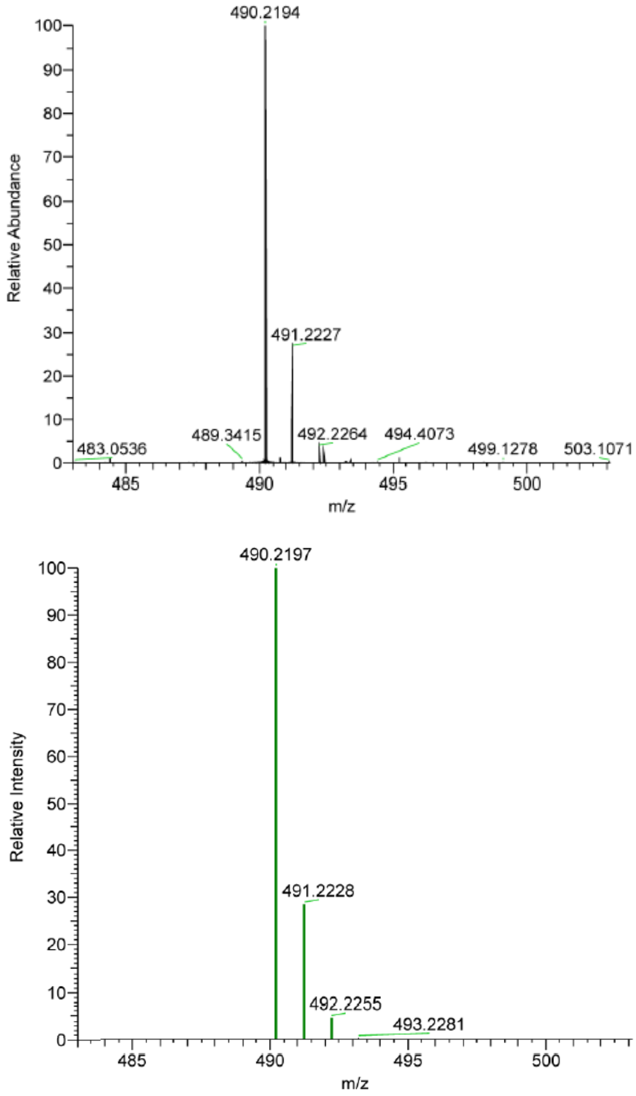

*Figure S1.3: High resolution ESI mass spectrum of macrocycle precursor **3** (top) and its theoretical spectrum (bottom)*

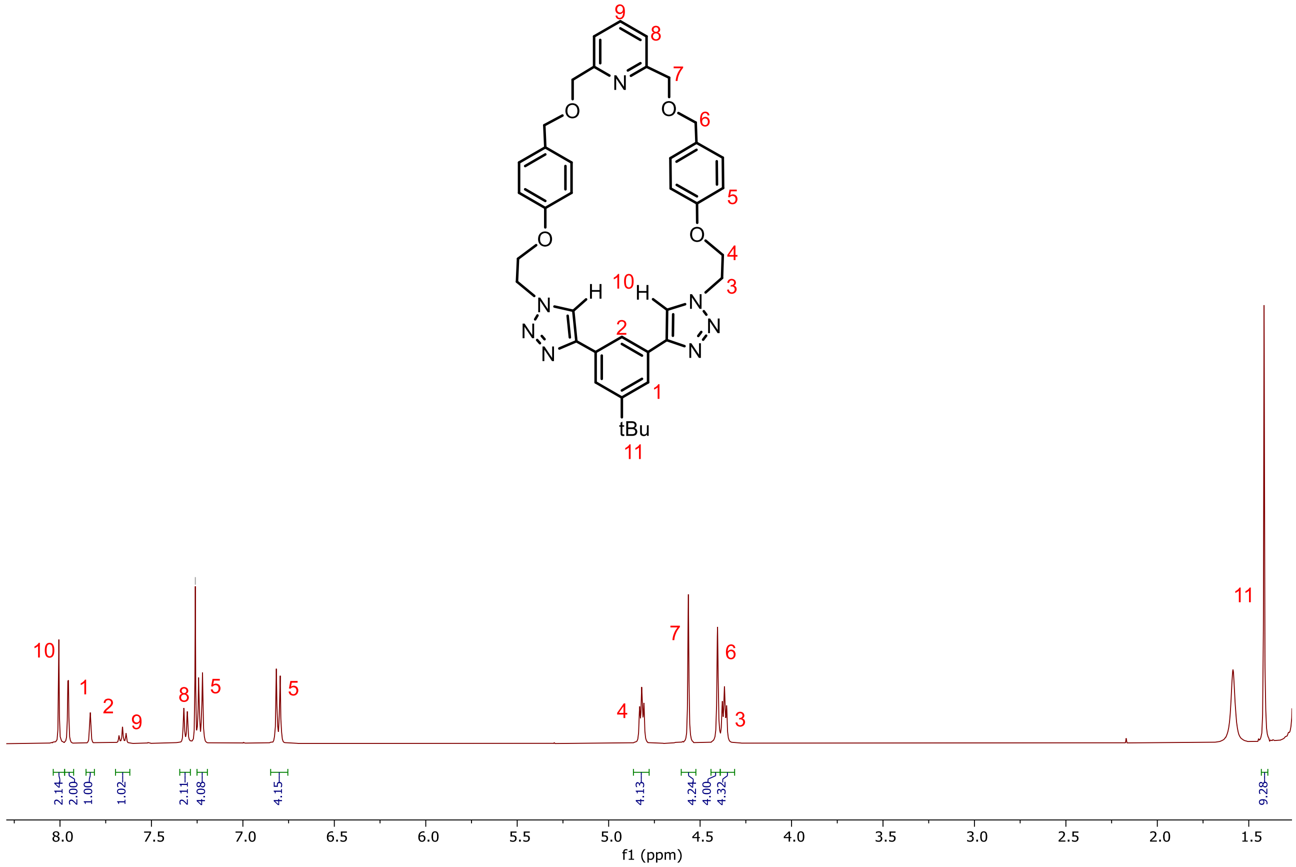

*Figure S1.4: ^{1}H NMR spectrum of macrocycle **5** (400 MHz, 298 K, $CDCl_3$).*

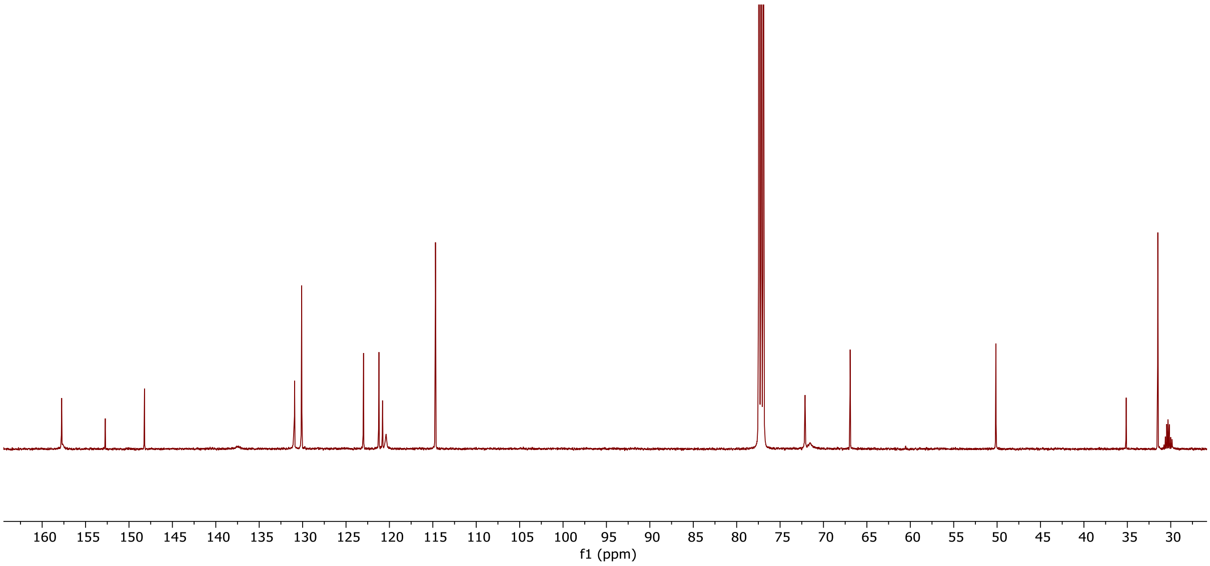

*Figure S1.5: ^{13}C NMR spectrum of macrocycle **5** (126 MHz, 298 K, $CDCl_3$).*

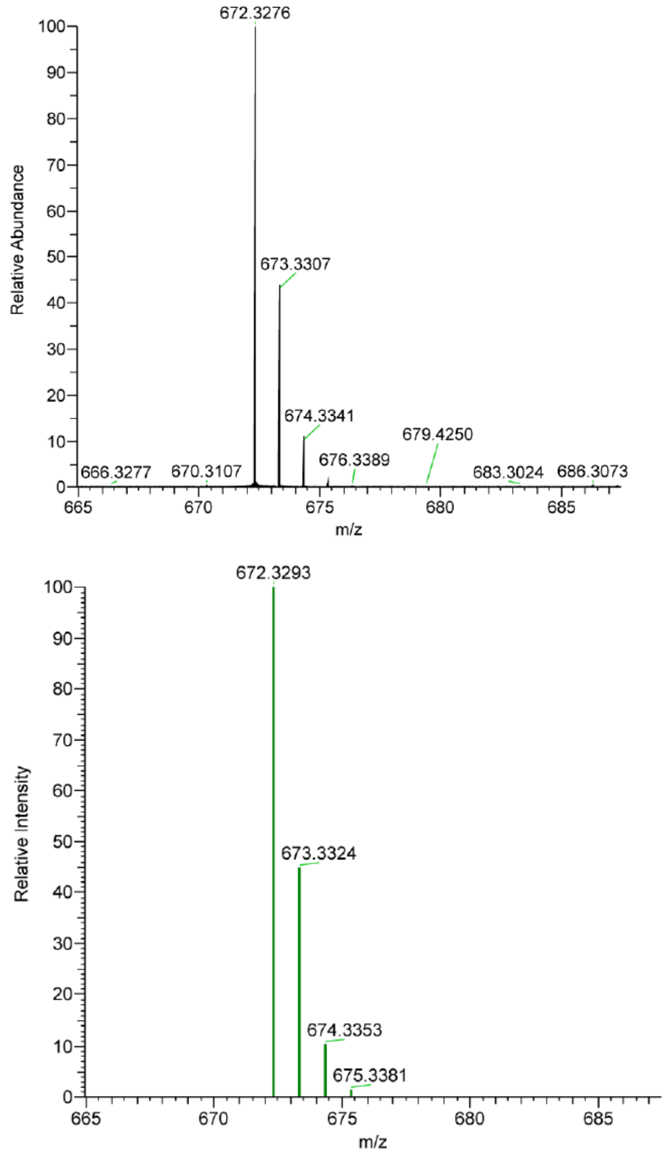

*Figure S1.6: High resolution ESI mass spectrum of macrocycle **5** (top) and its theoretical spectrum (bottom)*

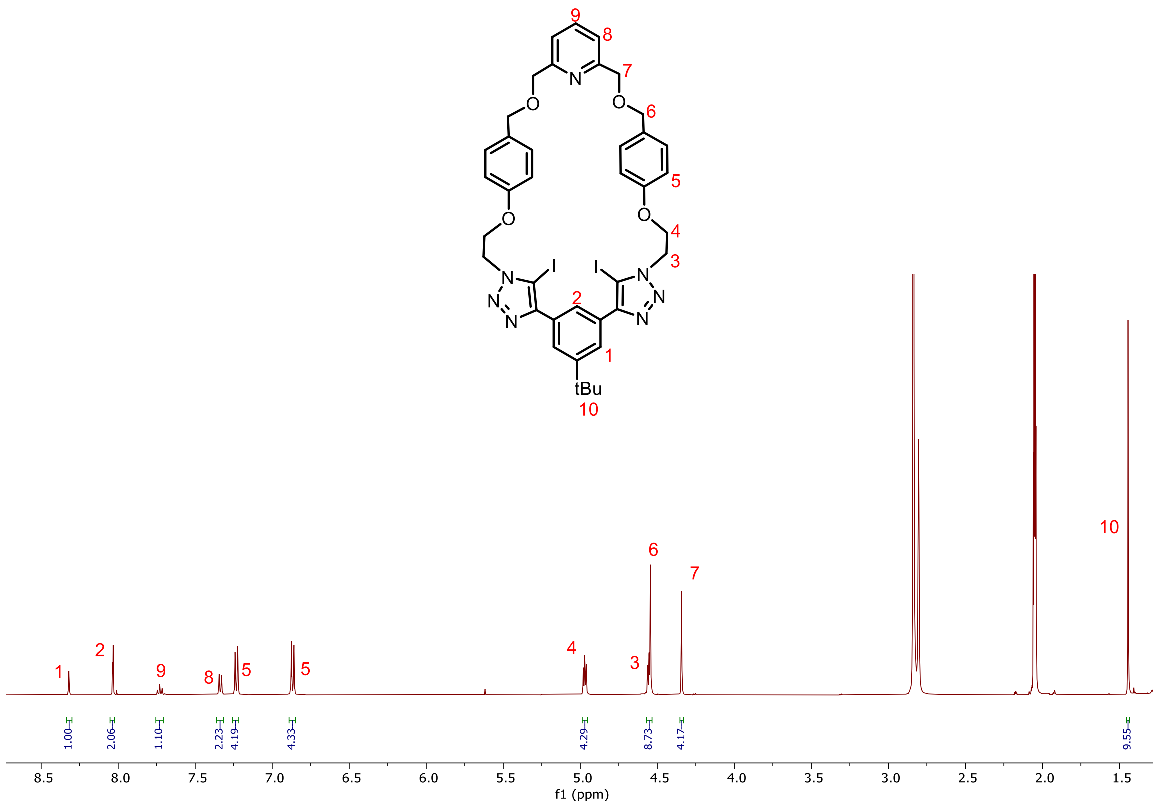

Figure S1.7: ^{1}H NMR spectrum of macrocycle **6** (500 MHz, 298 K, $(CD_3)_2CO$).

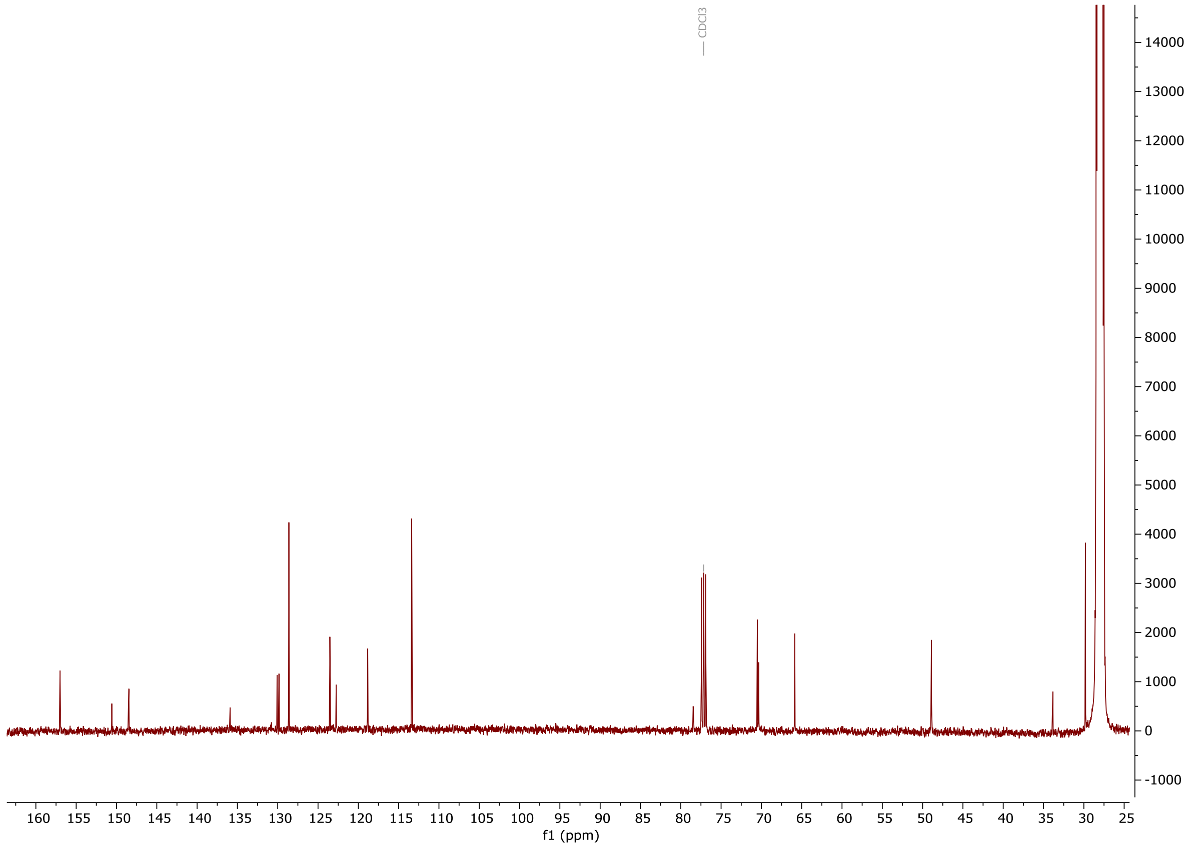

Figure S1.8: ^{13}C NMR spectrum of macrocycle **6** (126 MHz, 298 K, $(CD_3)_2CO$).

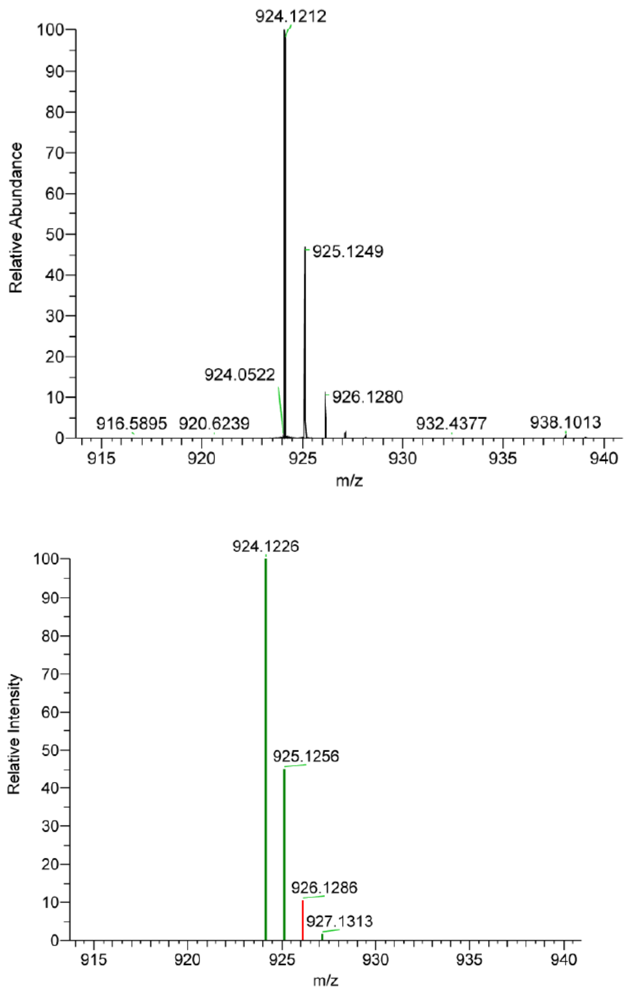

*Figure S1.9: High resolution ESI mass spectrum of macrocycle **6** (top) and its theoretical spectrum (bottom)*

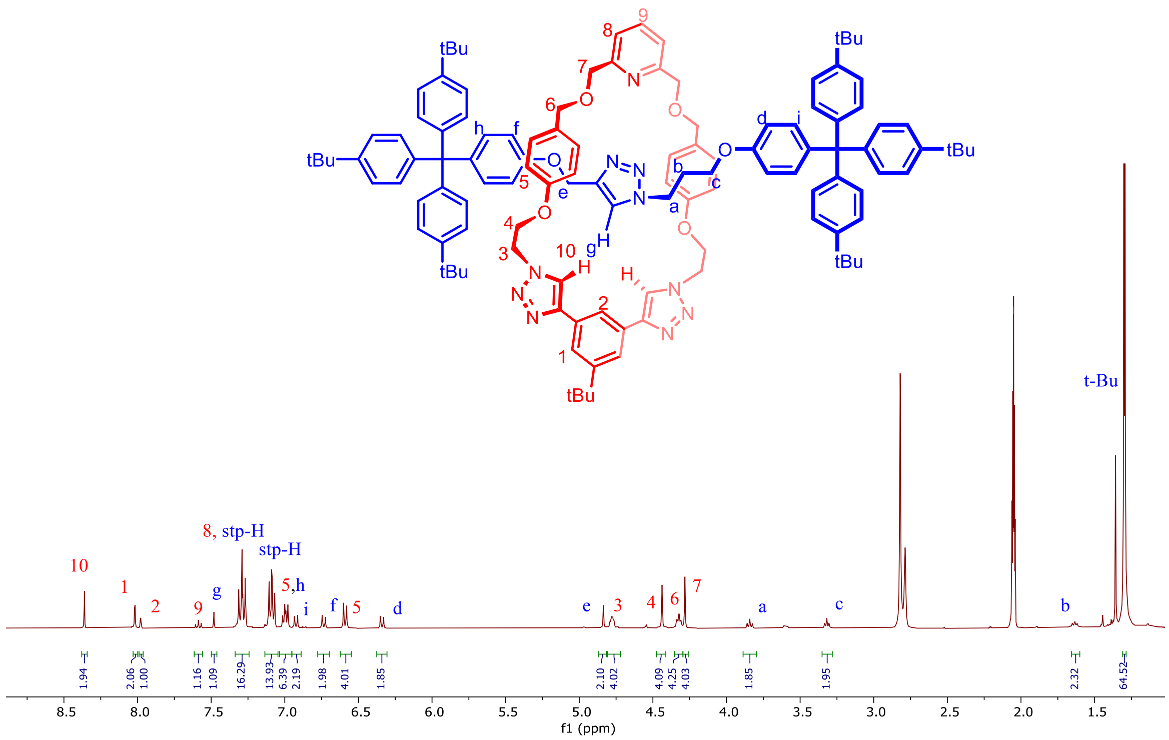

*Figure S1.10: ^{1}H NMR spectrum of Rotaxane **9** (500 MHz, 298 K, $(CD_3)_2CO$).*

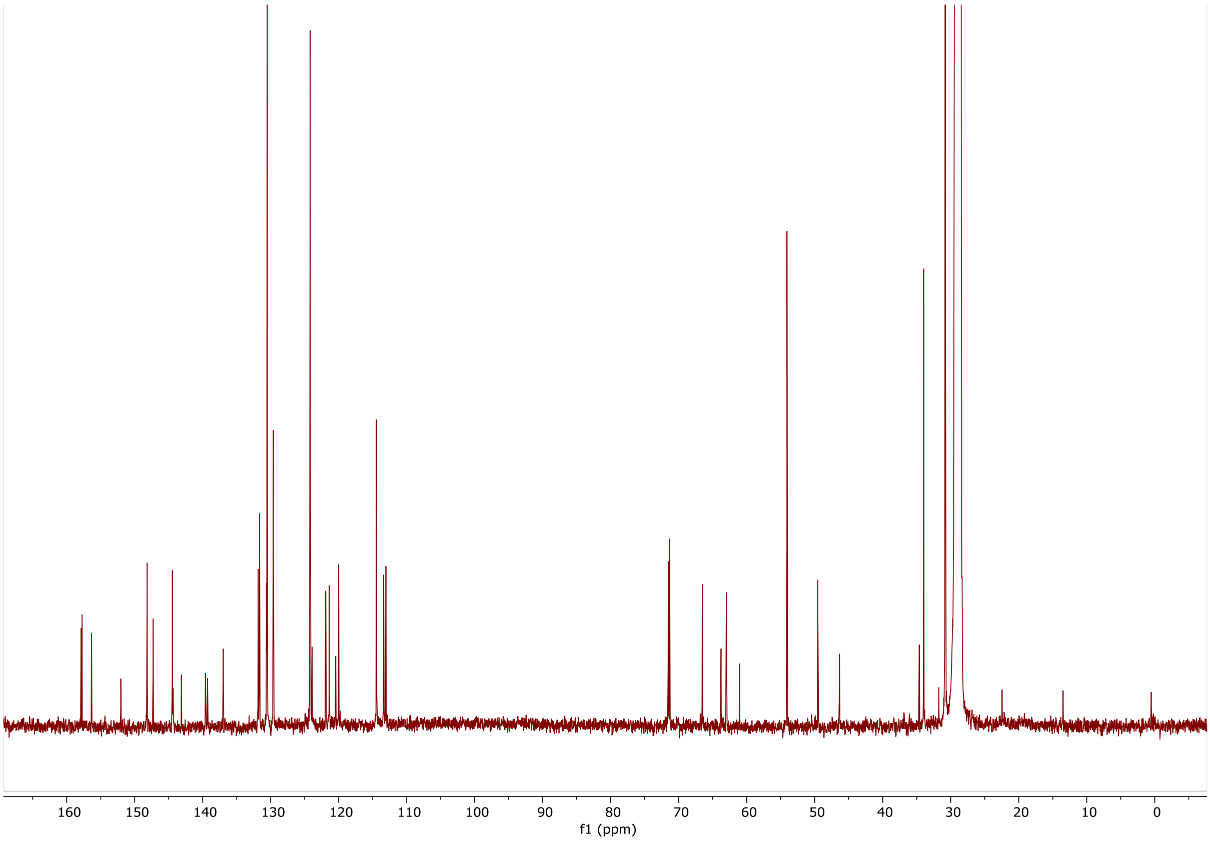

*Figure S1.11: ^{13}C NMR spectrum of Rotaxane **9** (126 MHz, 298 K, $(CD_3)_2CO$).*

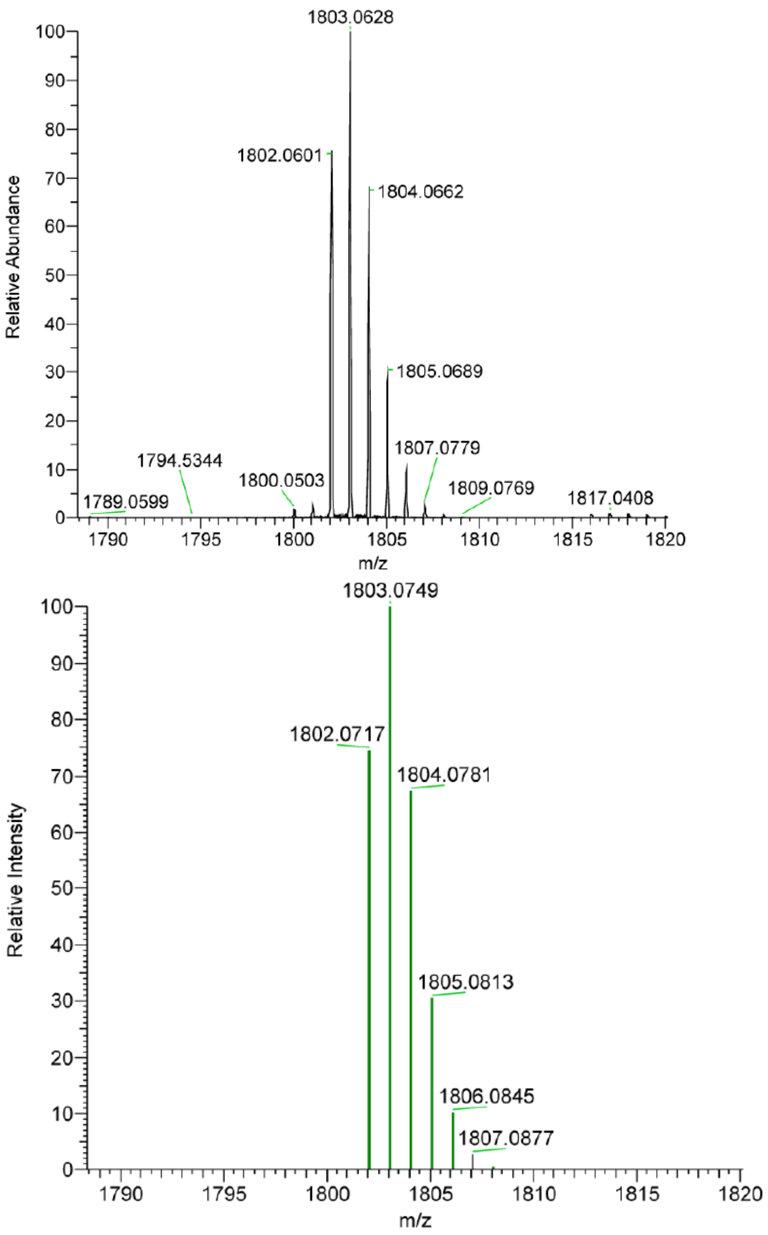

*Figure S1.12: High resolution ESI mass spectrum of Rotaxane **9** (top) and its theoretical spectrum (bottom)*

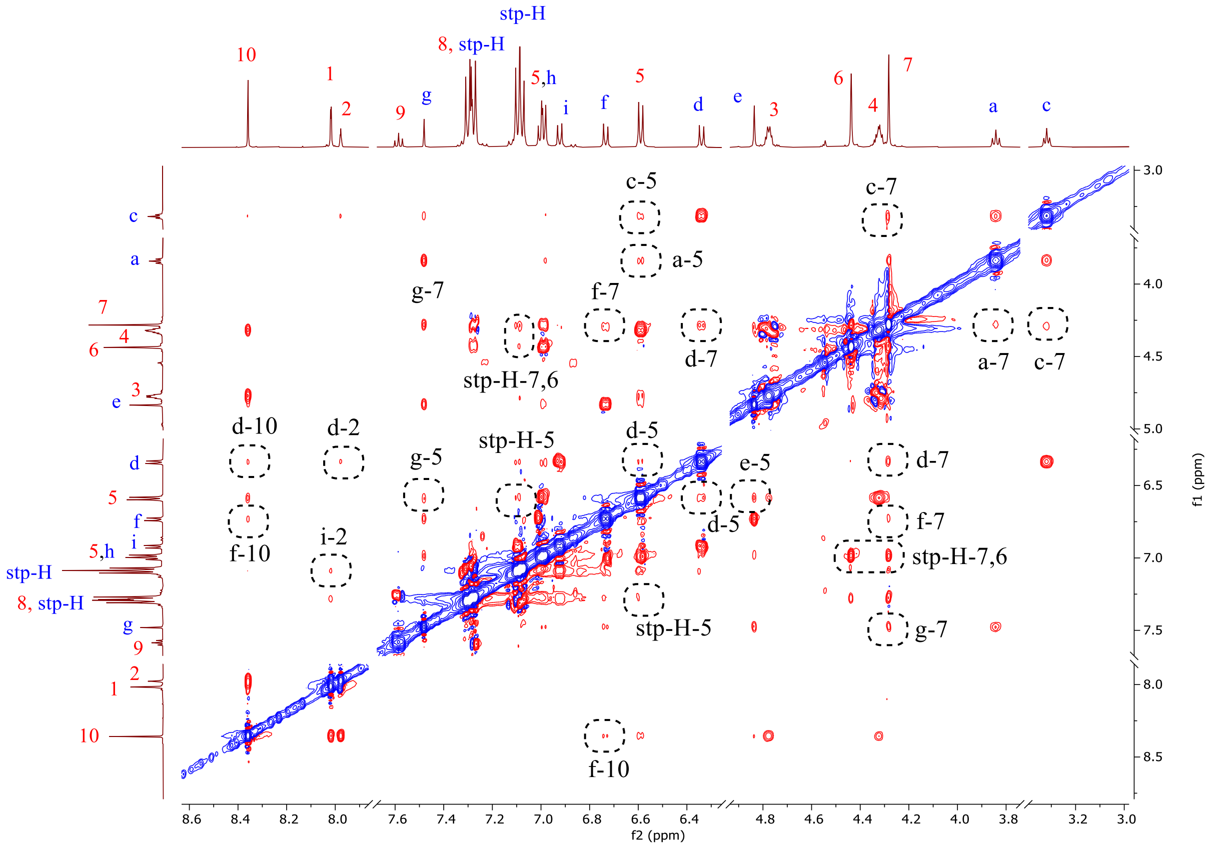

*Figure S1.13: ^1H-^1H ROESY NMR spectrum of the Rotaxane **9** ($(CD_3)_2CO$, 500 MHz, 298 K). Selected cross-peaks arising from through-space interactions of the interlocked macrocycle and axle components of the rotaxane are circled.*

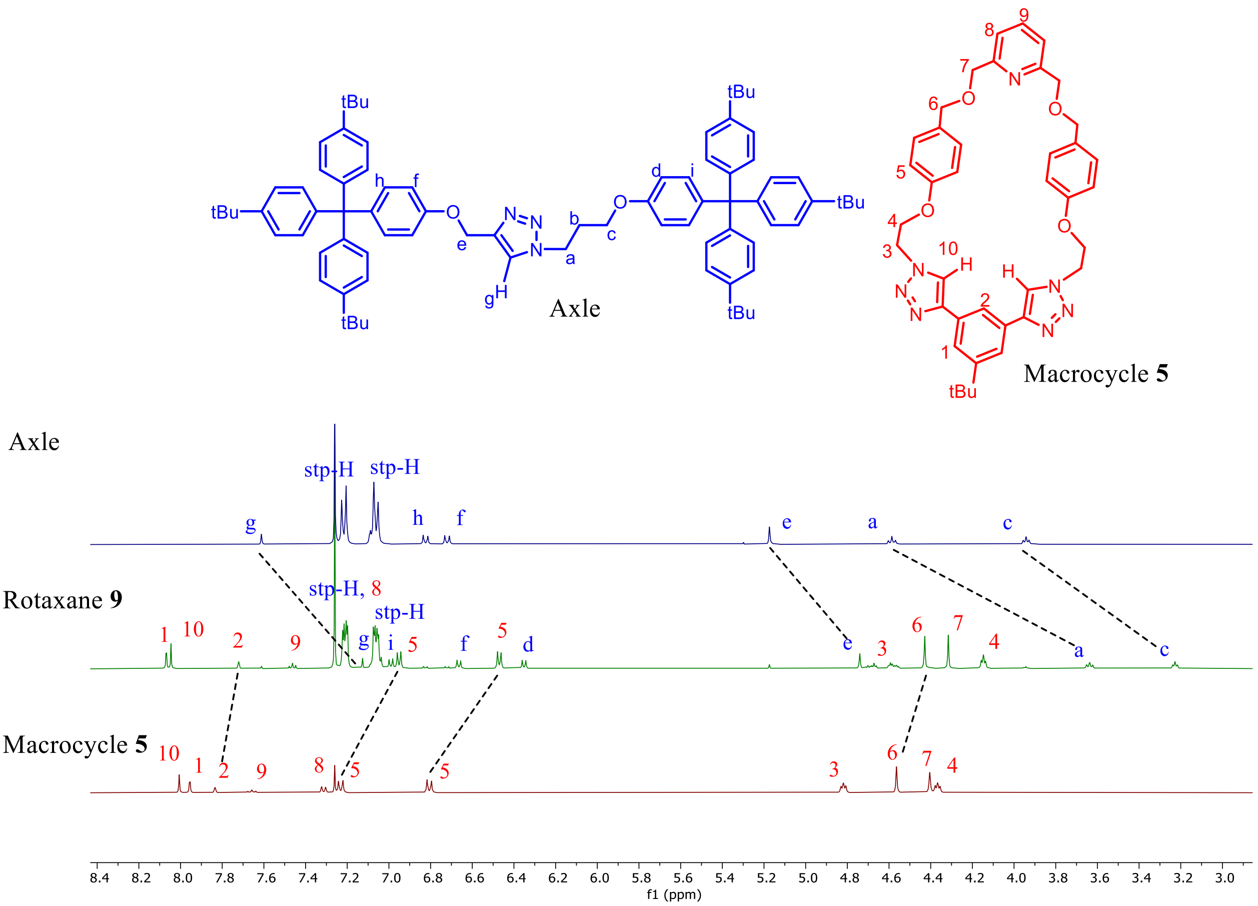

*Figure S1.14: Comparison of ^{1}H NMR spectra of axle (top), rotaxane **9** (middle) and macrocycle **5** (bottom) ($CDCl_3$, 500 MHz, 298 K)*

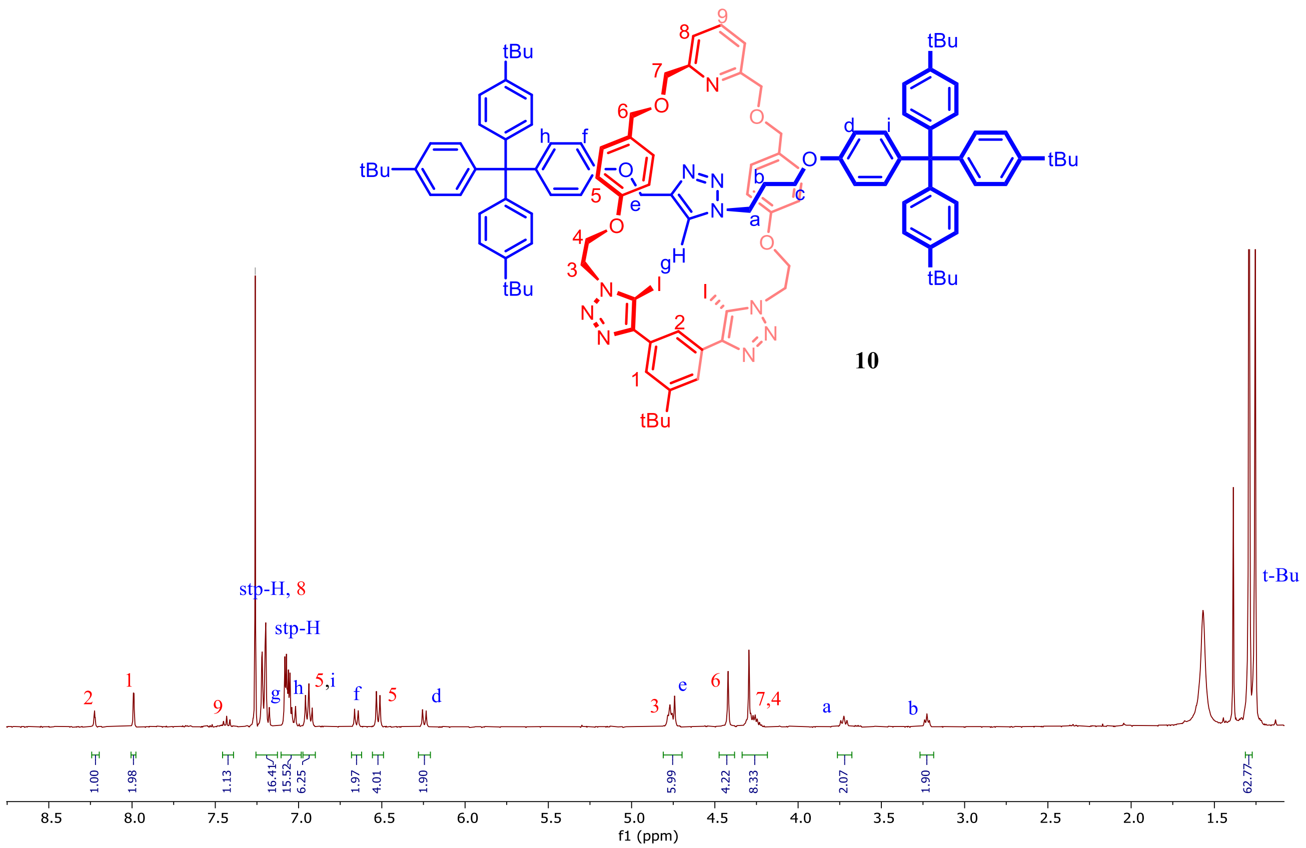

*Figure S1.15: ^{1}H NMR spectrum of Rotaxane **10** (400 MHz, 298 K, $CDCl_3$).*

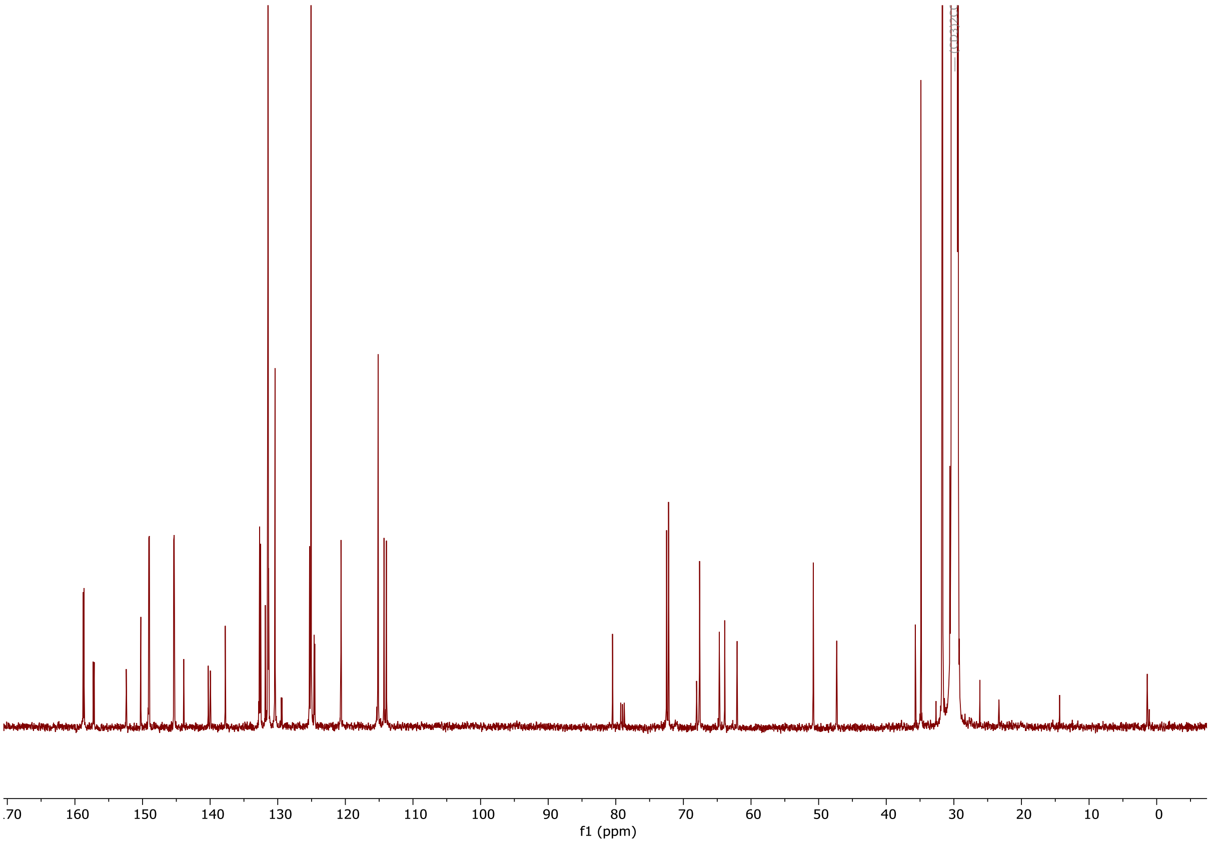

*Figure S1.16: ^{13}C NMR spectrum of Rotaxane **10** (126 MHz, 298 K, $(CD_3)_2CO$).*

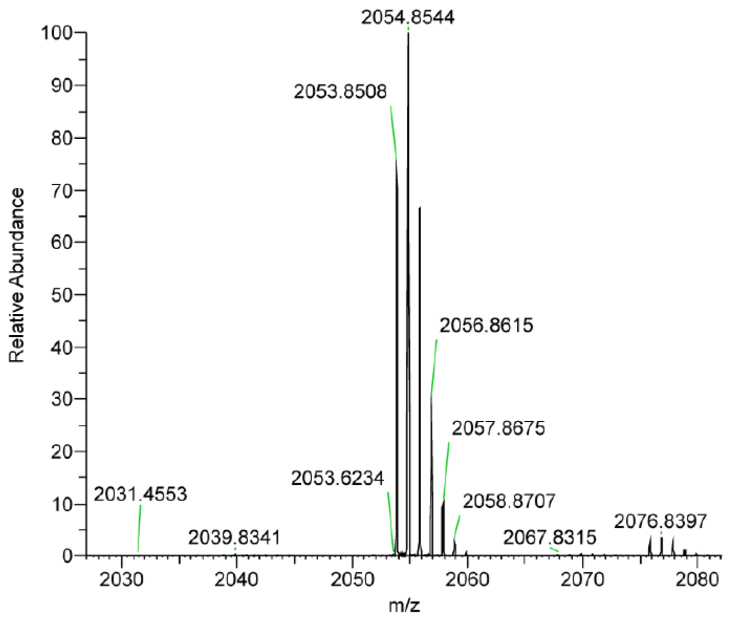

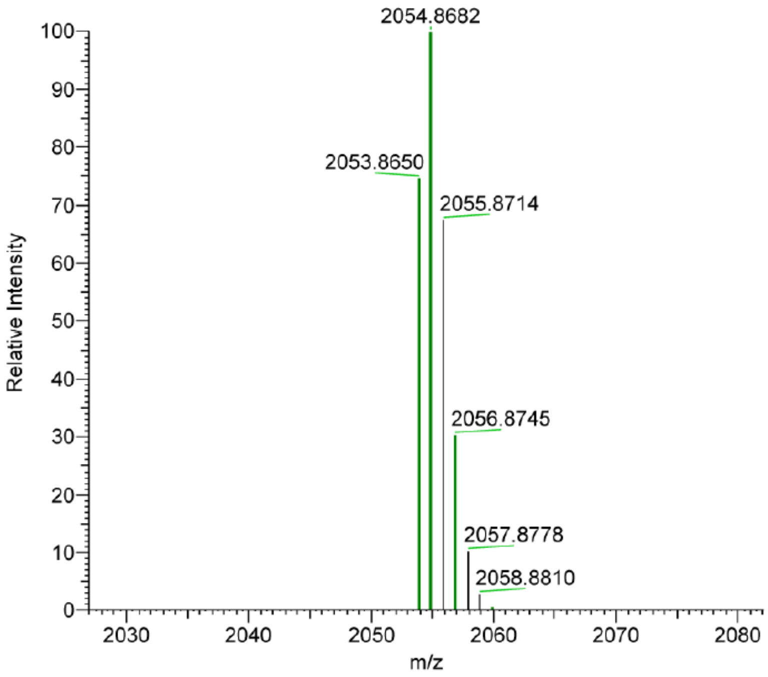

*Figure S1.17: High resolution ESI mass spectrum of Rotaxane **10** (top) and its theoretical spectrum (bottom)*

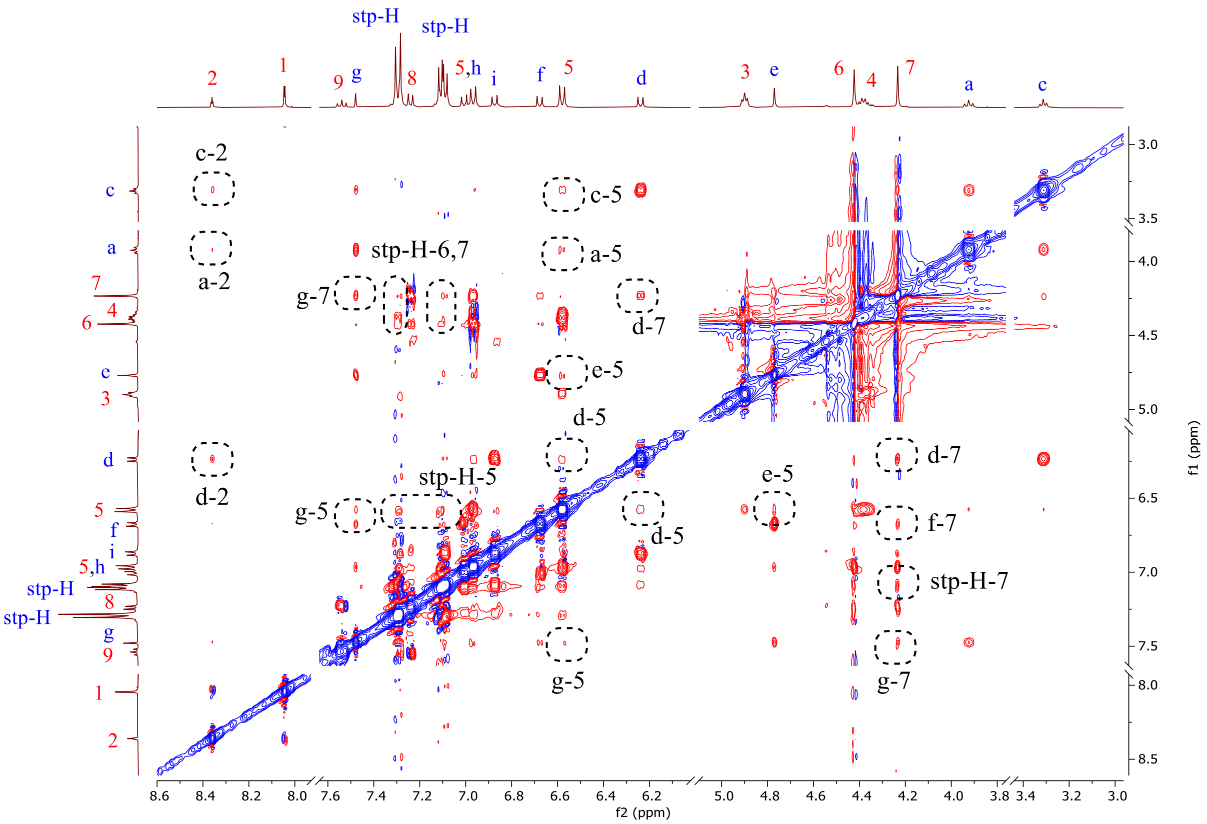

*Figure S1.18:^{1}H-^{1}H ROESY NMR spectrum of the Rotaxane **10** ($(CD_3)_2CO$, 500 MHz, 298 K). Selected cross-peaks arising from through-space interactions of the interlocked macrocycle and axle components of the rotaxane are circled.*

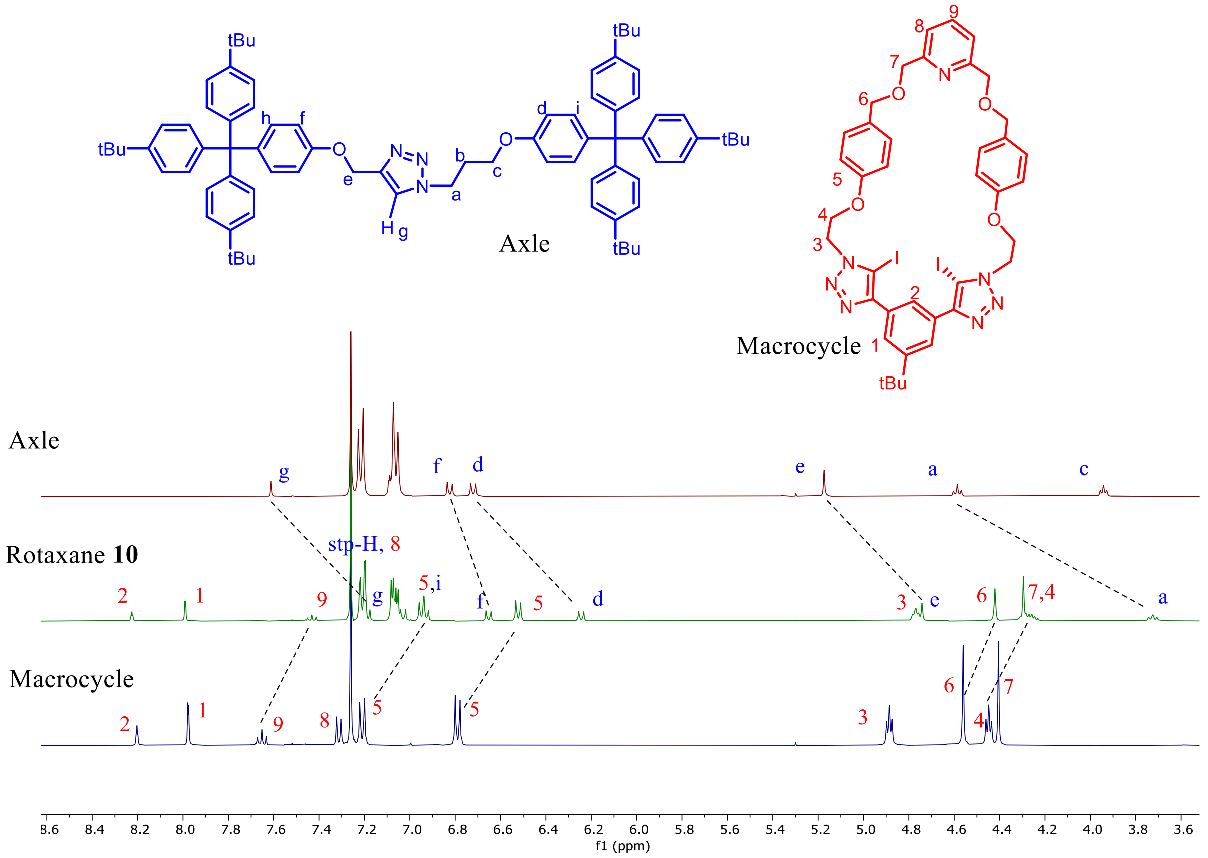

*Figure S1.19: Stacked ^{1}H NMR spectra of axle (top), rotaxane **10** (middle) and macrocycle **6** (bottom) ($CDCl_3$, 500 MHz, 298 K)*

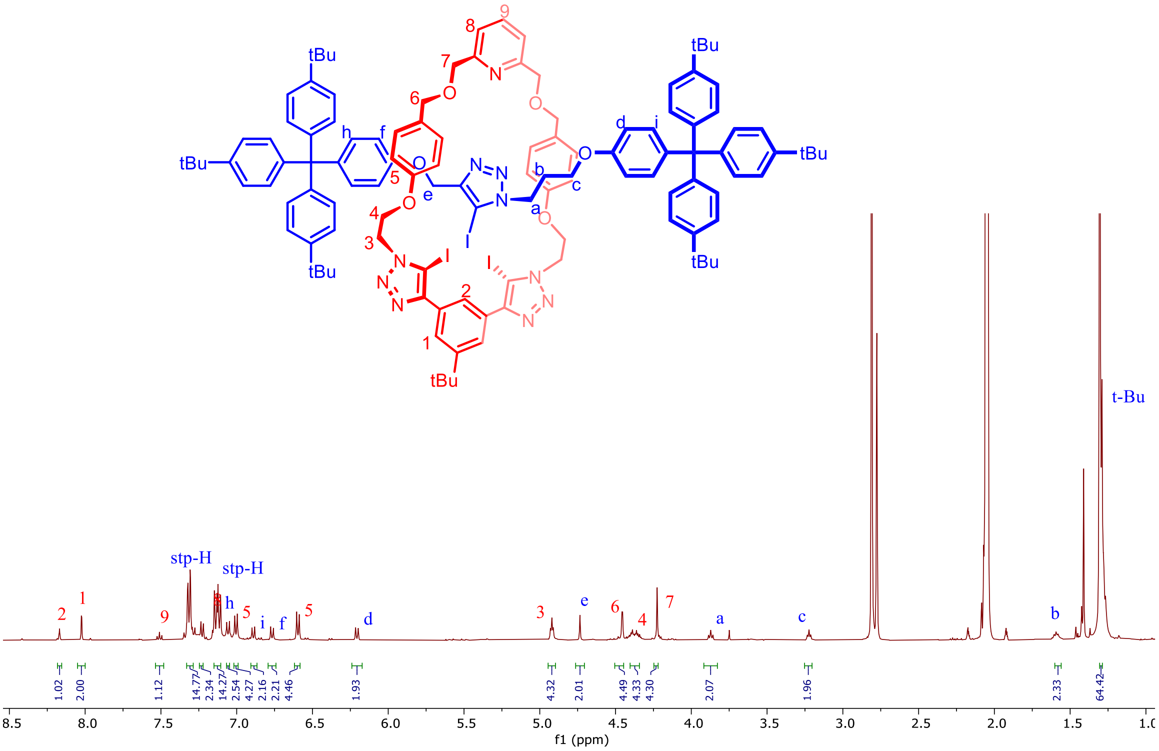

*Figure S1.20: ^{1}H NMR spectrum of Rotaxane **11** (500 MHz, 298 K, $(CD_3)_2CO$).*

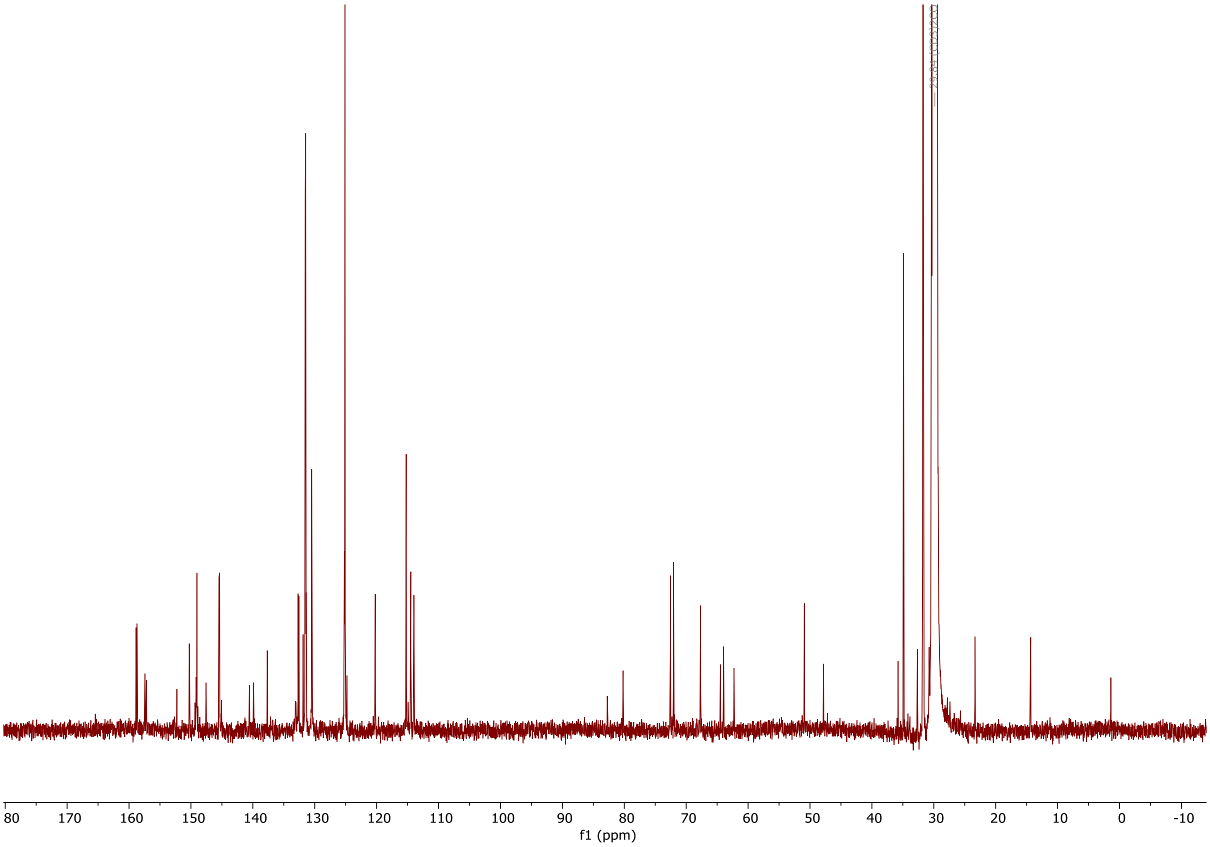

*Figure S1.21: ^{13}C NMR spectrum of Rotaxane **11** (126 MHz, 298 K, $(CD_3)_2CO$).*

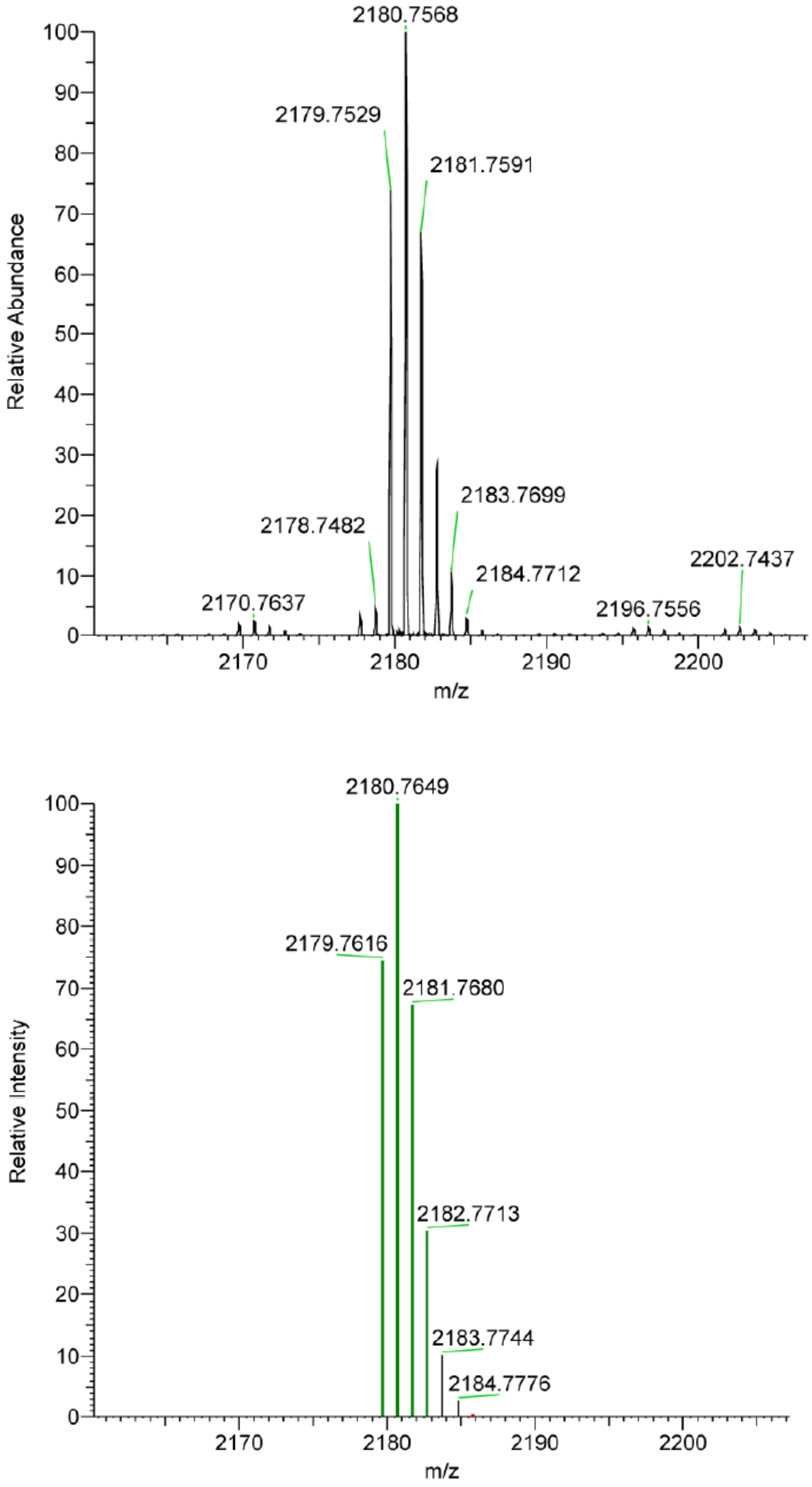

*Figure S1.22: High resolution ESI mass spectrum of Rotaxane **11** (top) and its theoretical spectrum (bottom)*

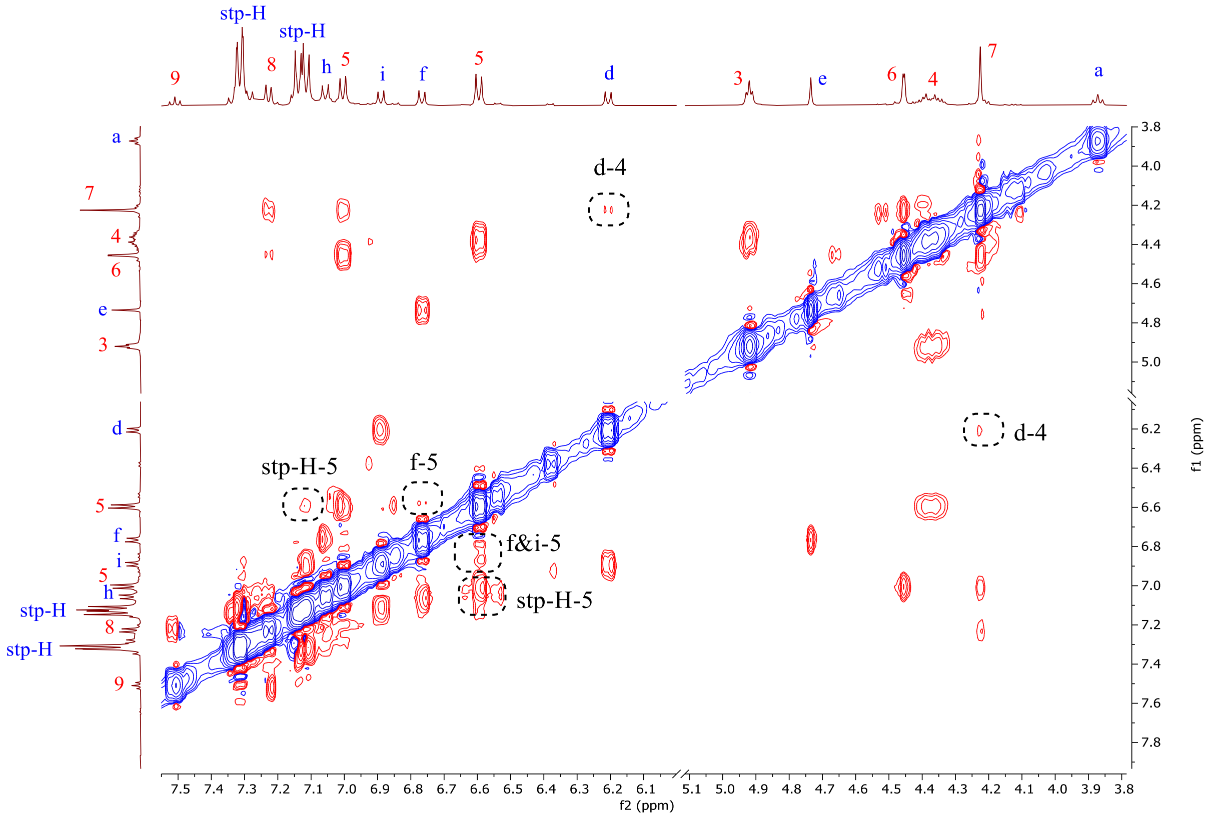

*Figure S1.23: :^{1}H-^{1}H ROESY NMR spectrum of Rotaxane **11** $(CD_3)_2CO$, 500 MHz, 298 K). Selected cross-peaks arising from through-space interactions of the interlocked macrocycle and axle components of the rotaxane are circled.*

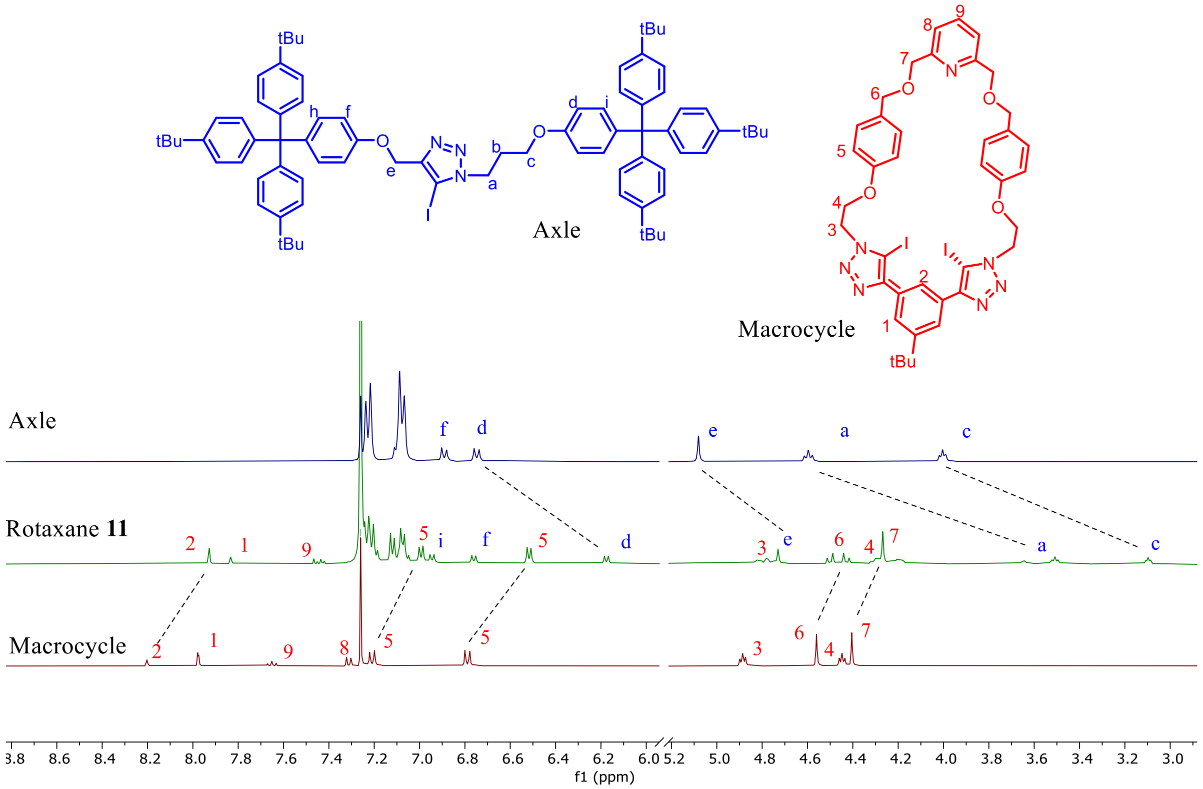

*Figure S1.24: Comparison of ^{1}H NMR spectra of axle (top), rotaxane **11** (middle) and macrocycle **6** (bottom) in $CDCl_3$, 500 MHz, 298 K*

S2. Solid State Structures

A complete data set for macrocycle **6.HCl** (CCDC 2164636) was collected. Single crystal samples for x-ray diffraction were obtained by slow evaporation of the $CHCl_3$ solution of compound **6** and 10 equivalents of TBACl. Single crystals of compound **6** suitable for X-ray analysis was coated with Paratone-N oil, suspended in a small fibre loop, and placed in a cold gaseous nitrogen stream on an Oxford Diffraction Supernova X-ray diffractometer performing ϕ- and ω-scans at 150(2) K. Diffraction intensities were measured using graphite monochromated Cu $K\alpha$ radiation (λ = 1.54184 Å). Data collection, indexing, initial cell refinements, frame integration, final cell refinements and absorption corrections were accomplished using the program Crysalispro[6]. Scattering factors and anomalous dispersion corrections were taken from the *International Tables for X-ray Crystallography*. All structures were solved by direct methods using SHELXS-97[7] and refined against F^2 on all data by full-matrix least squares with SHELXL[8] following established refinement strategies.

All non-hydrogen atoms were refined anisotropically. All hydrogen atoms binding to carbon were included into the model at geometrically calculated positions and refined using a riding model. The isotropic displacement parameters of all hydrogen atoms were fixed to 1.2 times the U value of the atoms they are linked to (1.5 times for methyl groups). Details of the data quality and a summary of the residual values for the refinements are listed in Table S2.1.

Structural refinements revealed compound **6** crystallised in triclinic space group P-1 with Z=2. In each asymmetric unit there was one molecule of the N-protonated compound **6**, one $CHCl_3$ and one water molecule. Cooperative halogen bonding interaction was found between two iodine and Cl^- with a C-I…Cl distance of 3.168 Å and 3.237 Å. Additionally hydrogen bonding interactions were found between protonated pyridinyl NH and Cl^- as well, proving evidence for cooperative anion binding between multiple of sites.

*Table S2.1: Crystal data and structure refinement for compound **6.**HCl.*

Identification code	Macrocycle **6.HCl**	
Empirical formula	C40 H43 Cl4 I2 N7 O5	
Formula weight	1097.41	
Temperature	150(2) K	
Wavelength	1.54184 Å	
Crystal system	Triclinic	
Space group	P-1	
Unit cell dimensions	a = 9.3137(4) Å	α= 78.421(3)°.
	b = 14.9357(6) Å	β= 81.256(3)°.
	c = 17.3935(5) Å	γ = 75.301(4)°.
Volume	2279.65(16) Å^3	
Z	2	
Density (calculated)	1.599 Mg/m^3	
Absorption coefficient	13.398 mm^{-1}	
F(000)	1092	
Crystal size	0.35 x 0.20 x 0.15 mm^3	
Theta range for data collection	3.701 to 77.138°.	
Index ranges	-11<=h<=11, -14<=k<=18, -21<=l<=21	
Reflections collected	21749	
Independent reflections	9359 [R(int) = 0.0540]	
Completeness to theta = 67.684°	99.8 %	
Absorption correction	Semi-empirical from equivalents	
Max. and min. transmission	0.7541 and 0.5069	
Refinement method	Full-matrix least-squares on F^2	
Data / restraints / parameters	9359 / 2 / 526	
Goodness-of-fit on F^2	1.106	
Final R indices [I>2sigma(I)]	R1 = 0.0853, wR2 = 0.2098	
R indices (all data)	R1 = 0.0990, wR2 = 0.2322	
Largest diff. peak and hole	2.515 and -0.546 e.Å^{-3}	

S3. ^{1}H NMR binding studies

S3.1. General Procedure

All ^{1}H NMR titrations were performed on a Bruker Avance III 500 MHz NMR spectrometer, at 298 K, in $CDCl_3$:CD_3CN solvent mixtures. Initial sample volume of the NMR tube was 0.5 mL whilst concentrations were 1.0 mM of the receptor. Anions were added as their TBA salts and lithium was added as the ClO_4^- salt.

In a typical experiment with Li^+ bound receptor, uncomplexed receptor was dissolved in the respective CD_3CN:$CDCl_3$ solvent mixtures containing an equimolar of $LiClO_4$. Resultant mixture was sonicated. TBA salt of the anion was dissolved separately in a solvent mixture of the same CD_3CN:$CDCl_3$ ratio to make a 0.05 M of anion solution. Prepared anion solutions were added in aliquots to the lithium bound receptor solution and spectra were recorded upon addition of 0.0, 0.2, 0.4, 0.6, 0.8, 1.0, 1.2, 1.4, 1.6, 1.8, 2.0, 2.5, 3.0, 4.0, 5.0, 7.0, 10.0 equivalents of TBA-anion. In all instances, binding of the anion and cation was in fast exchange, allowing the calculation association constants using Bindfit program available from http://supramolecular.org, accessed December, 2021.[9]

S3.2. Ion-pair binding ^{1}H NMR titration experiments

^{1}H NMR titrations of rotaxane **9**

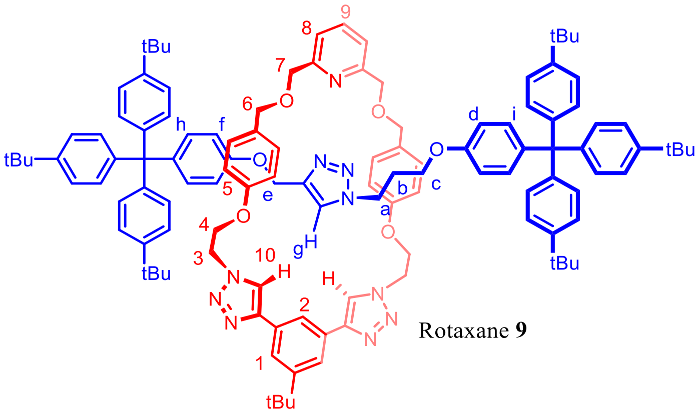

Rotaxane **9**

a)

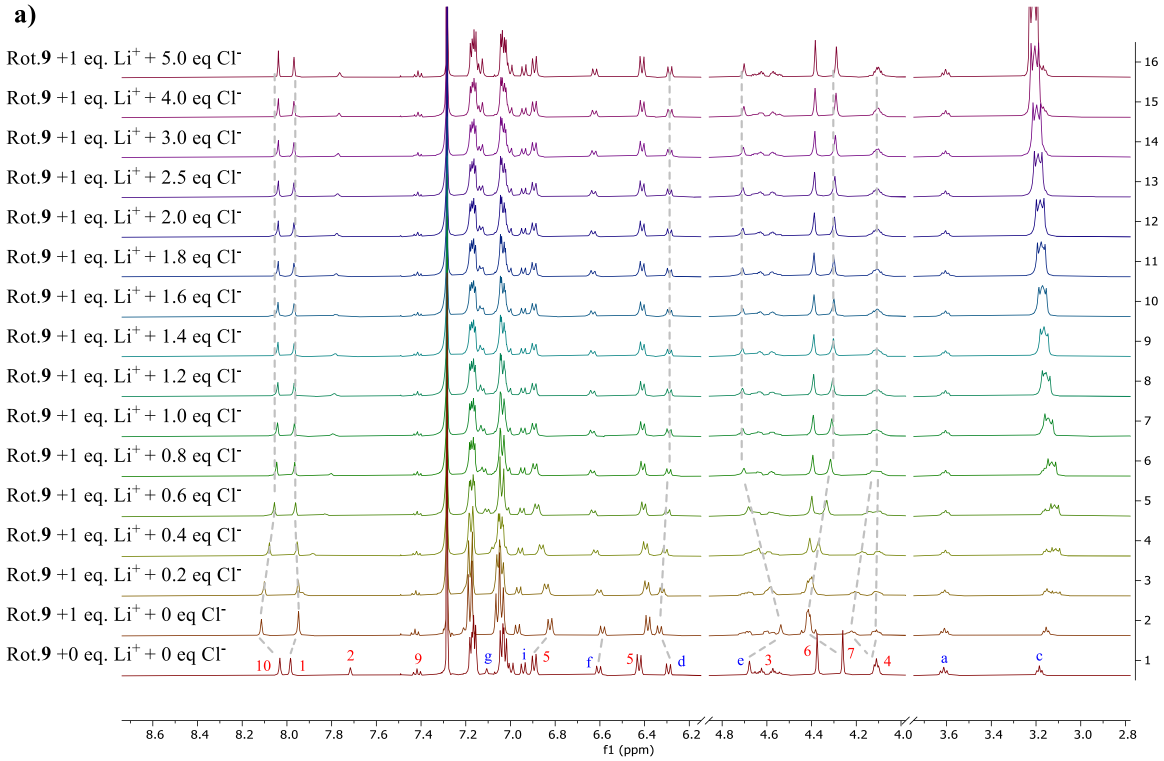

b)

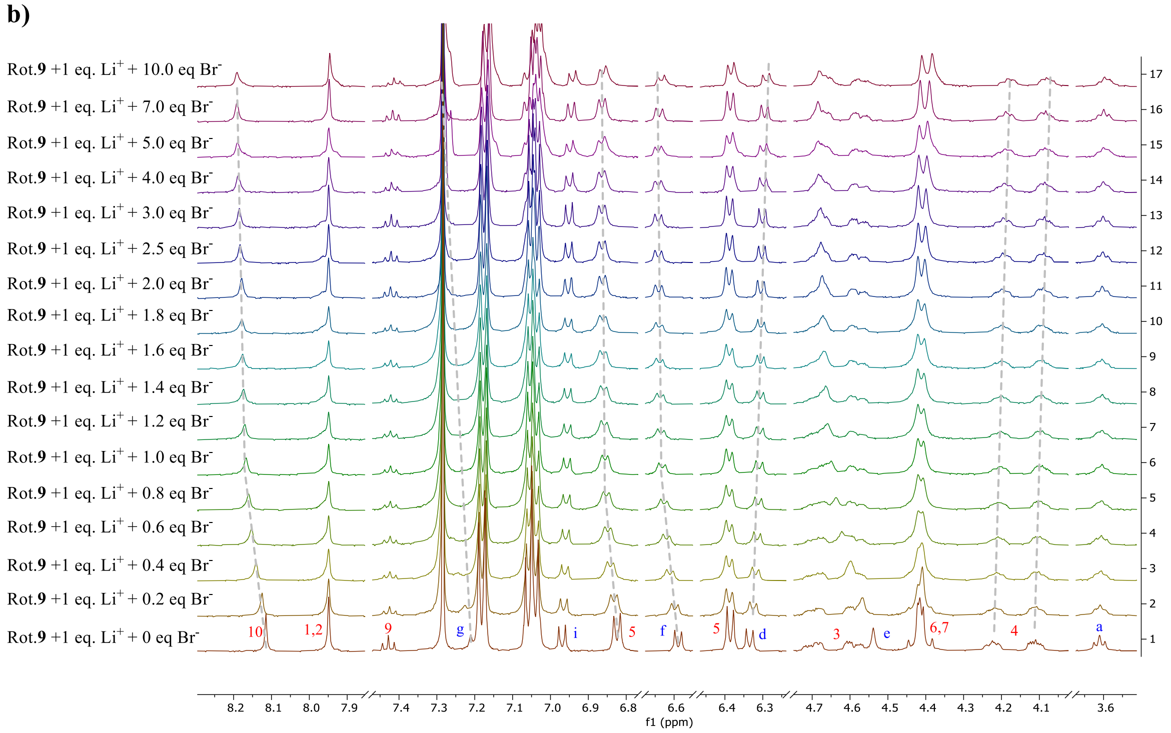

c)

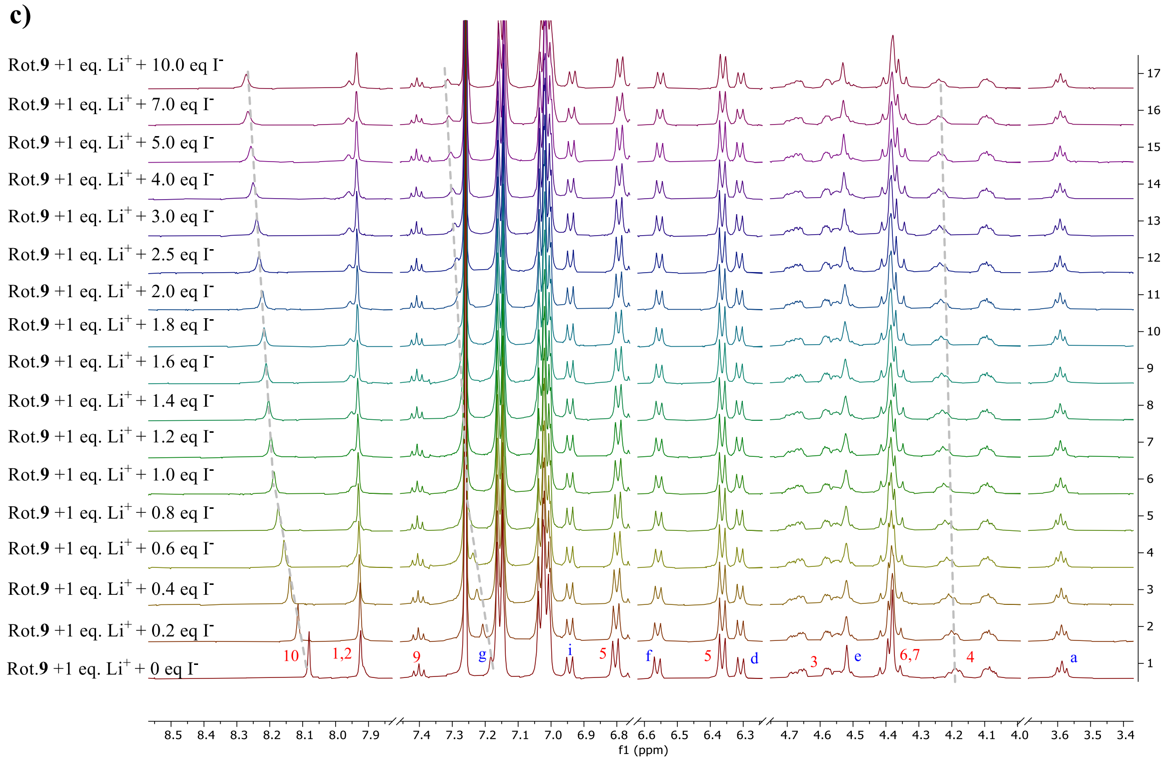

*Figure S3.1: Truncated ^{1}H NMR titration spectra of lithium precomplexed rotaxane **9** upon addition of (a)TBACl, (b) TBABr and (c) TBAI (500 MHz, 298 K, 9:1 $CDCl_3$:CD_3CN, [Rot.**9**] = 1.0 mM). (Connecting lines are to guide the eye only)*

^{1}H NMR titrations of rotaxane **10**

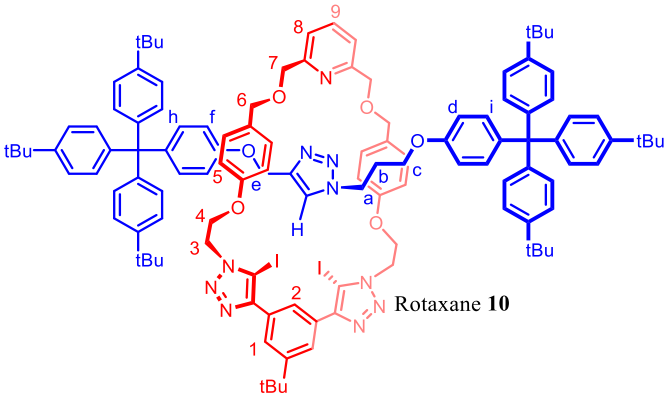

Rotaxane **10**

a)

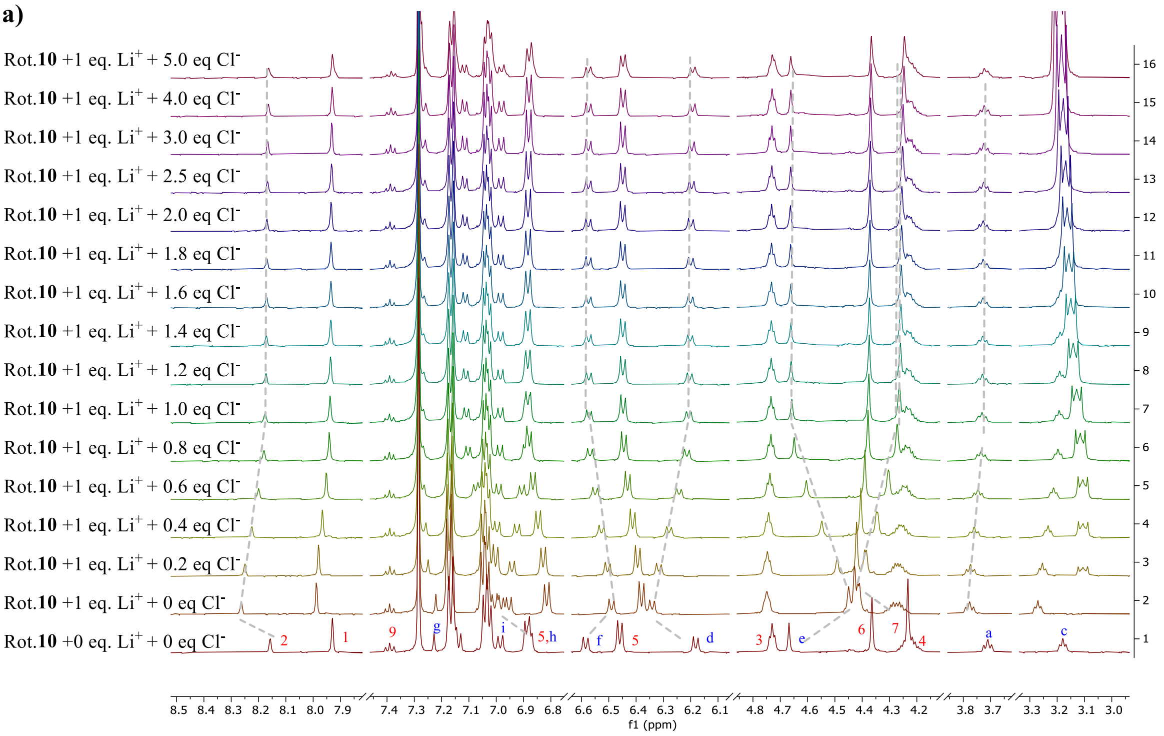

b)

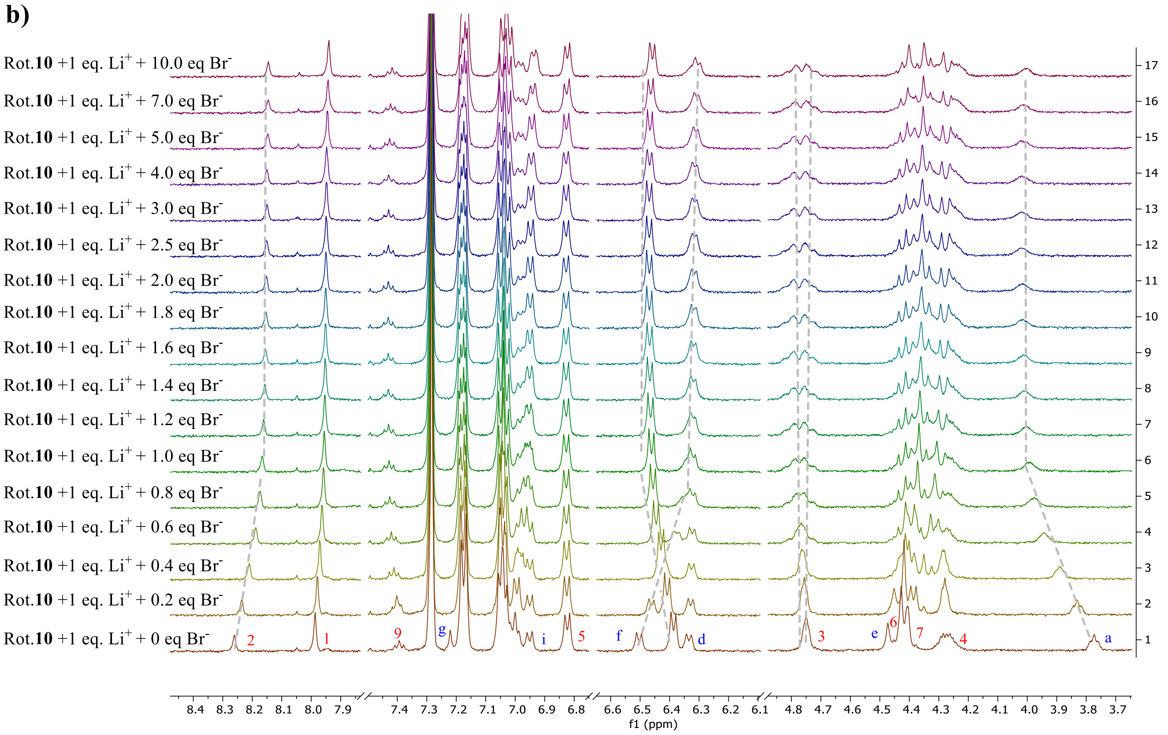

c)

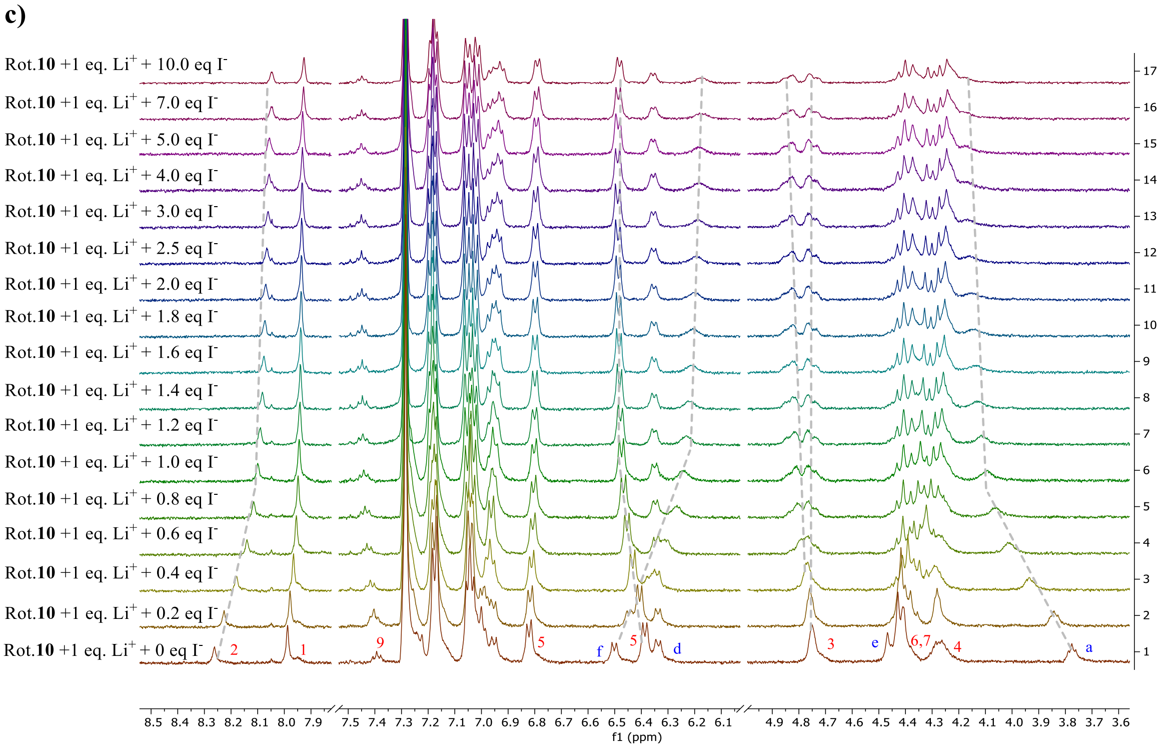

*Figure S3.2: Truncated ^{1}H NMR titration spectra of lithium precomplexed rotaxane **10** upon addition of (a)TBACl, (b) TBABr and (c) TBAI (500 MHz, 298 K, 9:1 $CDCl_3$:CD_3CN, [Rot.**10**] = 1.0 mM). (Connecting lines are to guide the eye only)*

a)

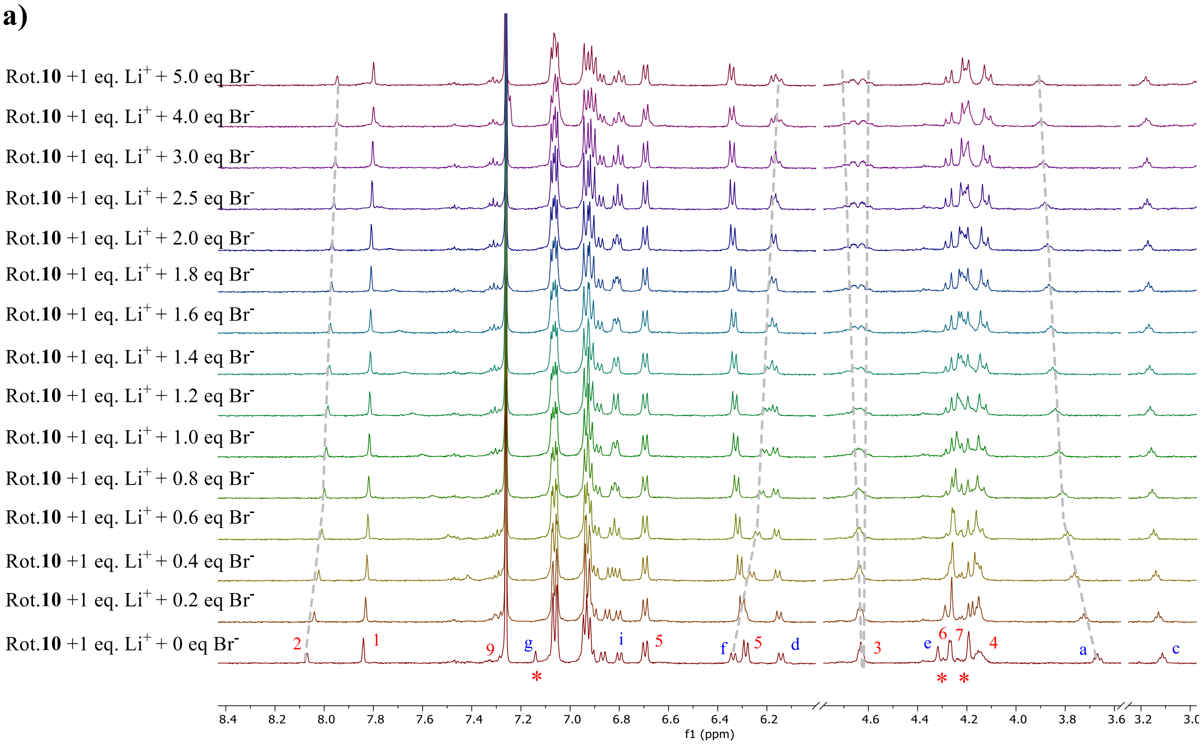

Rot.10 +1 eq. Li+ + 5.0 eq Br-
Rot.10 +1 eq. Li+ + 4.0 eq Br-
Rot.10 +1 eq. Li+ + 3.0 eq Br-
Rot.10 +1 eq. Li+ + 2.5 eq Br-
Rot.10 +1 eq. Li+ + 2.0 eq Br-
Rot.10 +1 eq. Li+ + 1.8 eq Br-
Rot.10 +1 eq. Li+ + 1.6 eq Br-
Rot.10 +1 eq. Li+ + 1.4 eq Br-
Rot.10 +1 eq. Li+ + 1.2 eq Br-
Rot.10 +1 eq. Li+ + 1.0 eq Br-
Rot.10 +1 eq. Li+ + 0.8 eq Br-
Rot.10 +1 eq. Li+ + 0.6 eq Br-
Rot.10 +1 eq. Li+ + 0.4 eq Br-
Rot.10 +1 eq. Li+ + 0.2 eq Br-
Rot.10 +1 eq. Li+ + 0 eq Br-
2 1 9 g i 5 f 5 d 3 e 6 7 4 a c
* * *
8.4 8.2 8.0 7.8 7.6 7.4 7.2 7.0 6.8 6.6 6.4 6.2 4.6 4.4 4.2 4.0 3.8 3.6 3.2 3.0
f1 (ppm)

b)

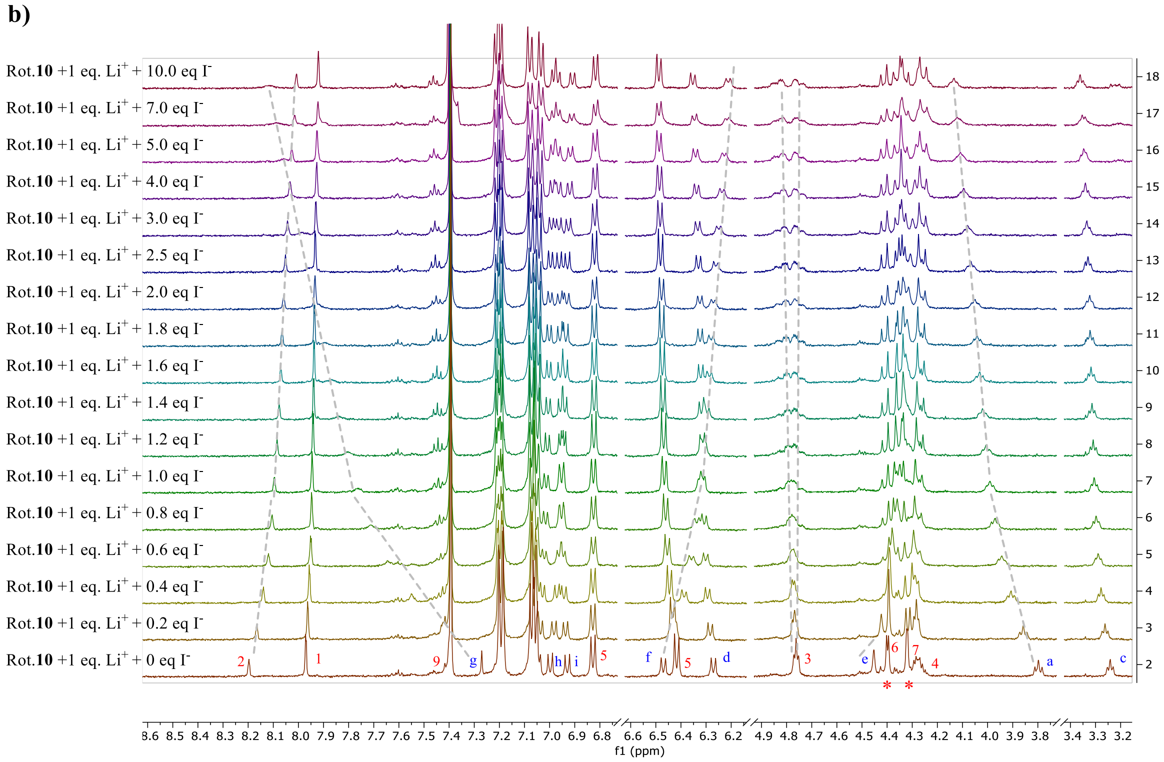

Rot.10 +1 eq. Li+ + 10.0 eq I-
Rot.10 +1 eq. Li+ + 7.0 eq I-
Rot.10 +1 eq. Li+ + 5.0 eq I-
Rot.10 +1 eq. Li+ + 4.0 eq I-
Rot.10 +1 eq. Li+ + 3.0 eq I-
Rot.10 +1 eq. Li+ + 2.5 eq I-
Rot.10 +1 eq. Li+ + 2.0 eq I-
Rot.10 +1 eq. Li+ + 1.8 eq I-
Rot.10 +1 eq. Li+ + 1.6 eq I-
Rot.10 +1 eq. Li+ + 1.4 eq I-
Rot.10 +1 eq. Li+ + 1.2 eq I-
Rot.10 +1 eq. Li+ + 1.0 eq I-
Rot.10 +1 eq. Li+ + 0.8 eq I-
Rot.10 +1 eq. Li+ + 0.6 eq I-
Rot.10 +1 eq. Li+ + 0.4 eq I-
Rot.10 +1 eq. Li+ + 0.2 eq I-
Rot.10 +1 eq. Li+ + 0 eq I-
2 1 9 g h i 5 f 5 d 3 e 6 7 4 a c
* *
18 17 16 15 14 13 12 11 10 9 8 7 6 5 4 3 2
8.6 8.5 8.4 8.3 8.2 8.1 8.0 7.9 7.8 7.7 7.6 7.5 7.4 7.3 7.2 7.1 7.0 6.9 6.8 6.6 6.5 6.4 6.3 6.2 4.9 4.8 4.7 4.6 4.5 4.4 4.3 4.2 4.1 4.0 3.9 3.8 3.4 3.3 3.2
f1 (ppm)

c)

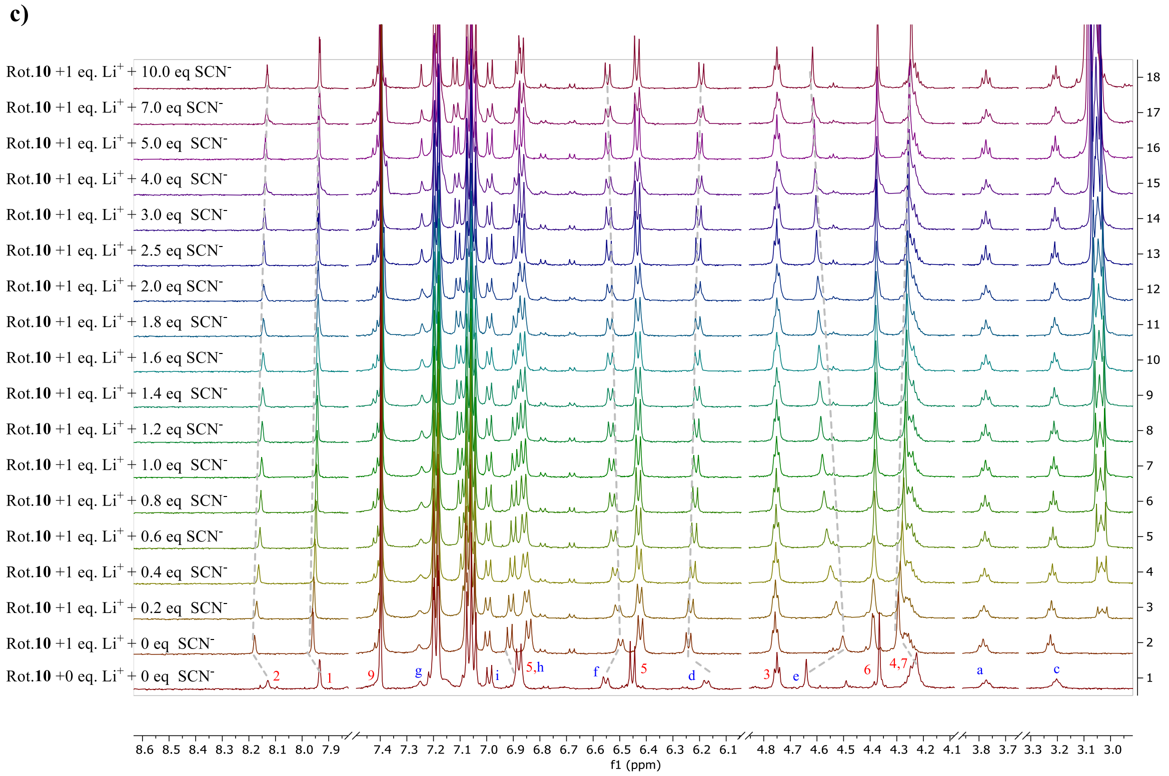
Rot.10 +1 eq. Li+ + 10.0 eq SCN-
Rot.10 +1 eq. Li+ + 7.0 eq SCN-
Rot.10 +1 eq. Li+ + 5.0 eq SCN-
Rot.10 +1 eq. Li+ + 4.0 eq SCN-
Rot.10 +1 eq. Li+ + 3.0 eq SCN-
Rot.10 +1 eq. Li+ + 2.5 eq SCN-
Rot.10 +1 eq. Li+ + 2.0 eq SCN-
Rot.10 +1 eq. Li+ + 1.8 eq SCN-
Rot.10 +1 eq. Li+ + 1.6 eq SCN-
Rot.10 +1 eq. Li+ + 1.4 eq SCN-
Rot.10 +1 eq. Li+ + 1.2 eq SCN-
Rot.10 +1 eq. Li+ + 1.0 eq SCN-
Rot.10 +1 eq. Li+ + 0.8 eq SCN-
Rot.10 +1 eq. Li+ + 0.6 eq SCN-
Rot.10 +1 eq. Li+ + 0.4 eq SCN-
Rot.10 +1 eq. Li+ + 0.2 eq SCN-
Rot.10 +1 eq. Li+ + 0 eq SCN-
Rot.10 +0 eq. Li+ + 0 eq SCN-
2 1 9 g i 5,h f 5 d 3 e 6 4,7 a c
f1 (ppm)

d)

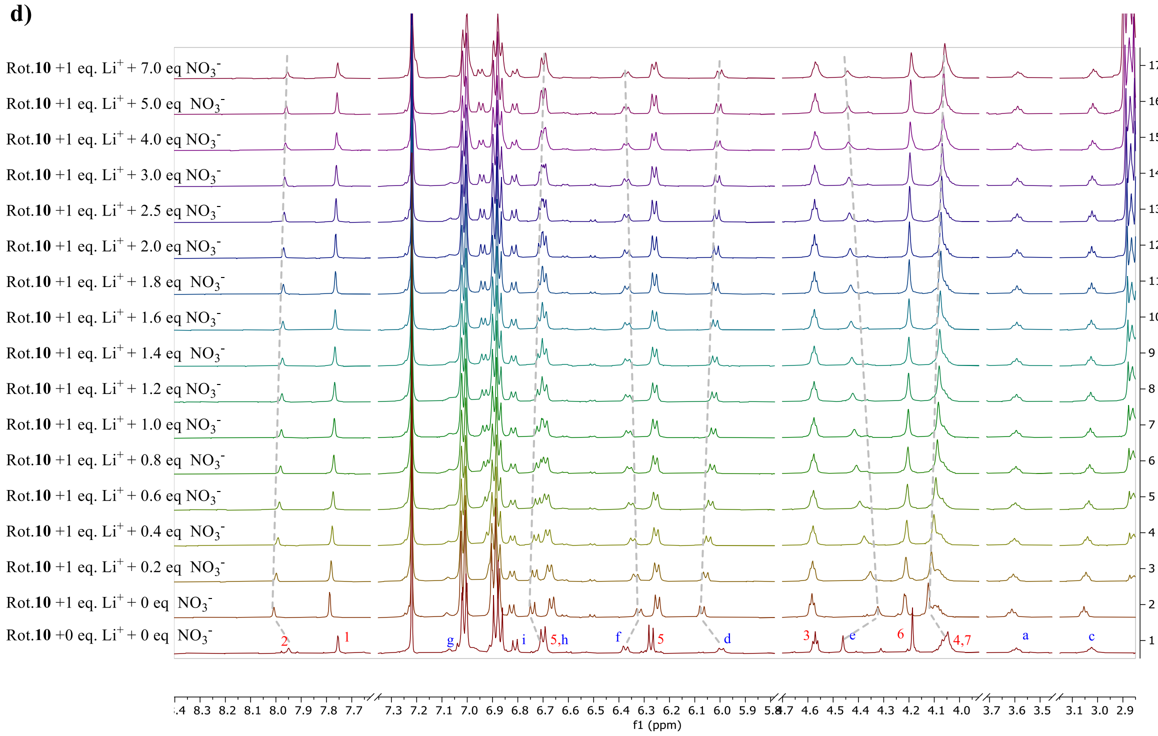
Rot.10 +1 eq. Li+ + 7.0 eq NO3-
Rot.10 +1 eq. Li+ + 5.0 eq NO3-
Rot.10 +1 eq. Li+ + 4.0 eq NO3-
Rot.10 +1 eq. Li+ + 3.0 eq NO3-
Rot.10 +1 eq. Li+ + 2.5 eq NO3-
Rot.10 +1 eq. Li+ + 2.0 eq NO3-
Rot.10 +1 eq. Li+ + 1.8 eq NO3-
Rot.10 +1 eq. Li+ + 1.6 eq NO3-
Rot.10 +1 eq. Li+ + 1.4 eq NO3-
Rot.10 +1 eq. Li+ + 1.2 eq NO3-
Rot.10 +1 eq. Li+ + 1.0 eq NO3-
Rot.10 +1 eq. Li+ + 0.8 eq NO3-
Rot.10 +1 eq. Li+ + 0.6 eq NO3-
Rot.10 +1 eq. Li+ + 0.4 eq NO3-
Rot.10 +1 eq. Li+ + 0.2 eq NO3-
Rot.10 +1 eq. Li+ + 0 eq NO3-
Rot.10 +0 eq. Li+ + 0 eq NO3-
2 1 g i 5,h f 5 d 3 e 6 4,7 a c
f1 (ppm)

e)

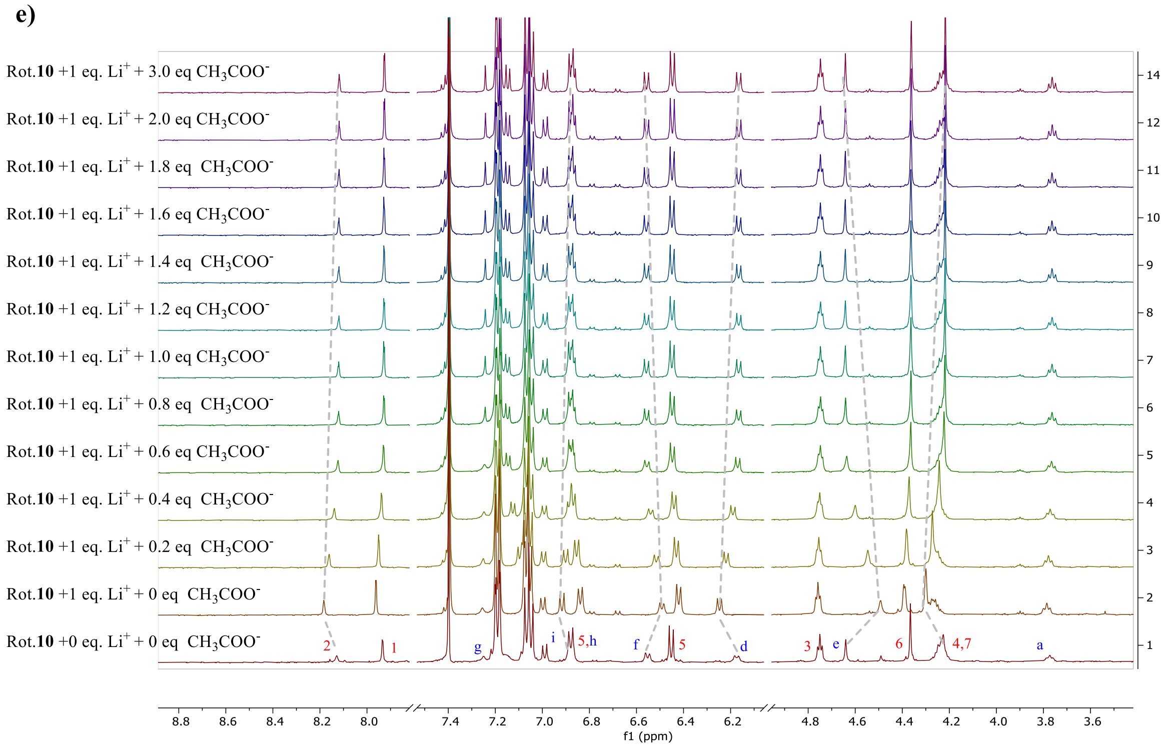

*Figure S3.3: Truncated ^{1}H NMR titration spectra of lithium precomplexed rotaxane **10** upon addition of (a)TBABr$^{\delta}$, (b) TBAI (c)TBASCN*, (d)TBANO$_3$*, (e)TBACH$_3$COO* (500 MHz, 298 K, 7:3 CDCl$_3$:CD$_3$CN, [Rot.**10**] = 1.0 mM). $^{\delta}$ precipitated after the addition of 7 eq. *H NMR peak shifts caused due to complexation of Li$^+$, reverted towards their original positions indicating metal decomplexation.*

^{1}H NMR titrations of rotaxane **11**

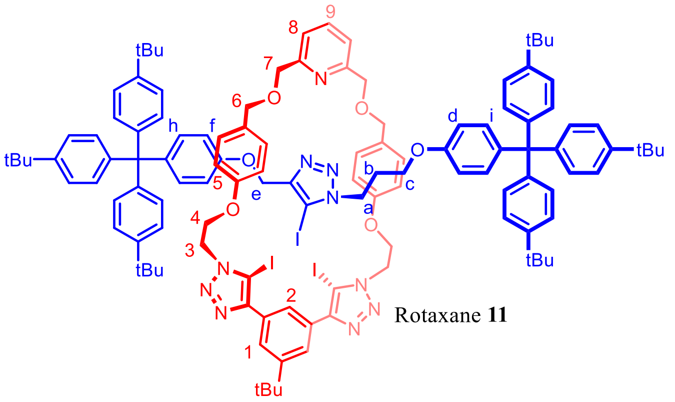

Rotaxane **11**

a)

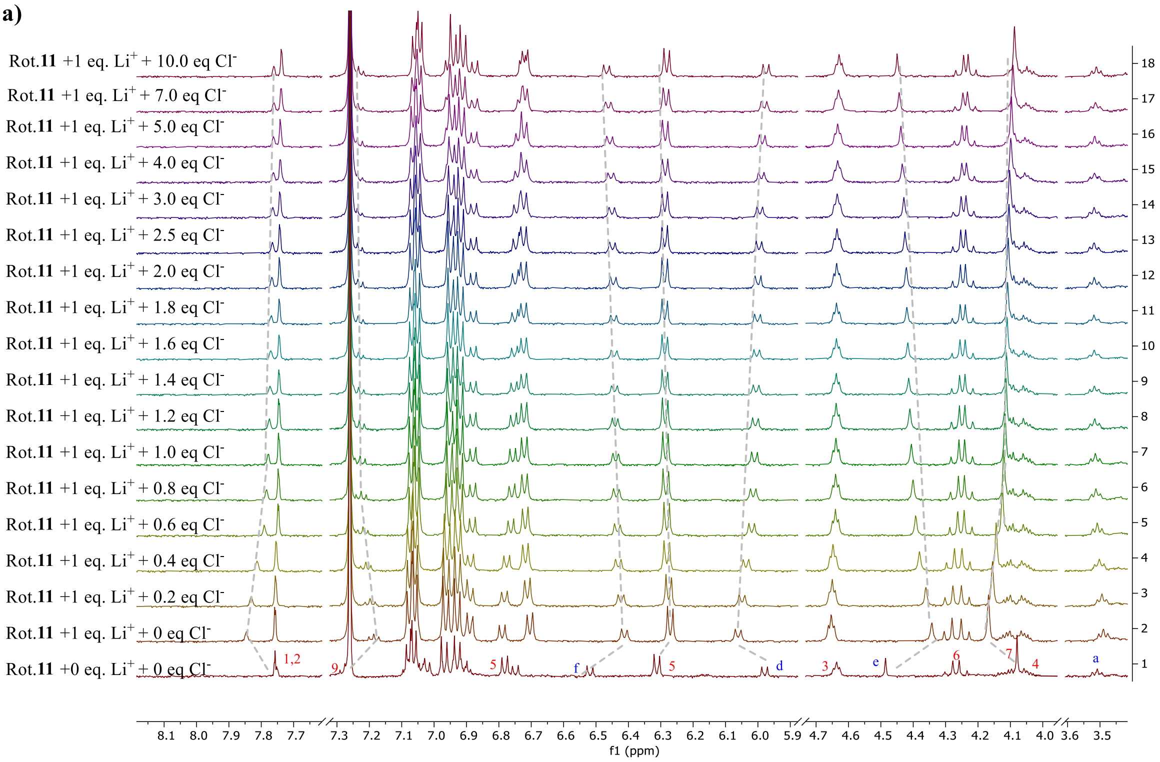

b)

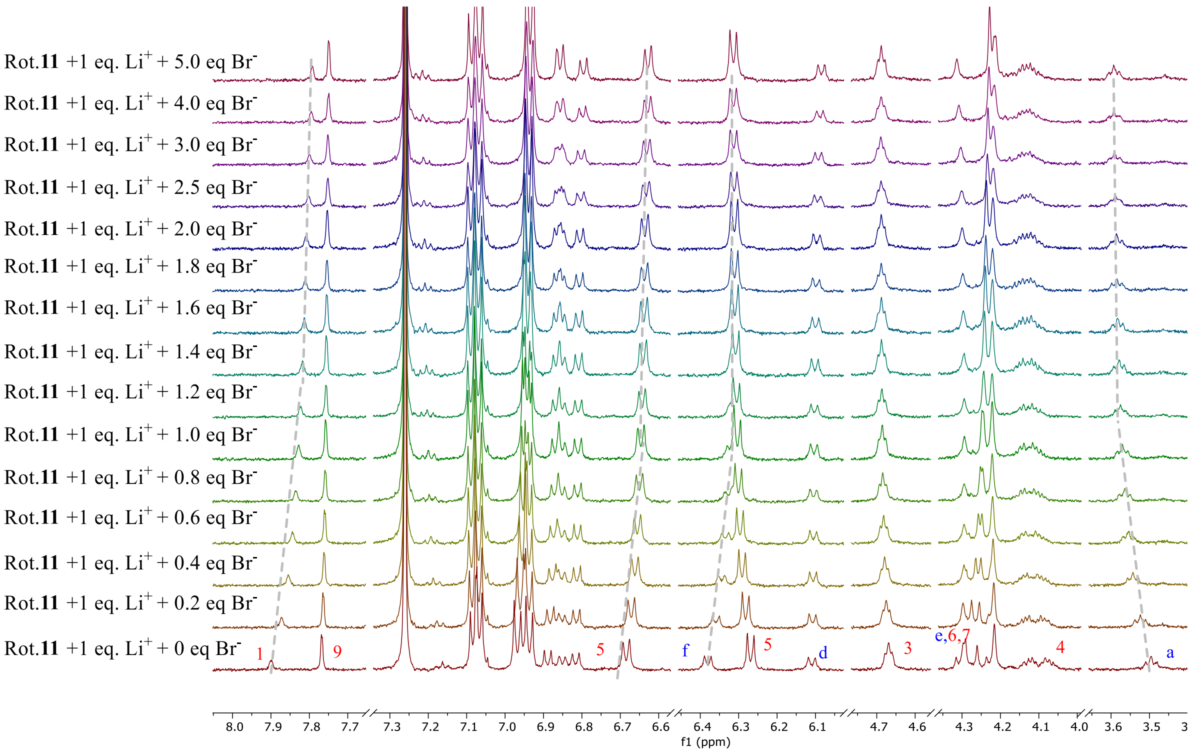

c)

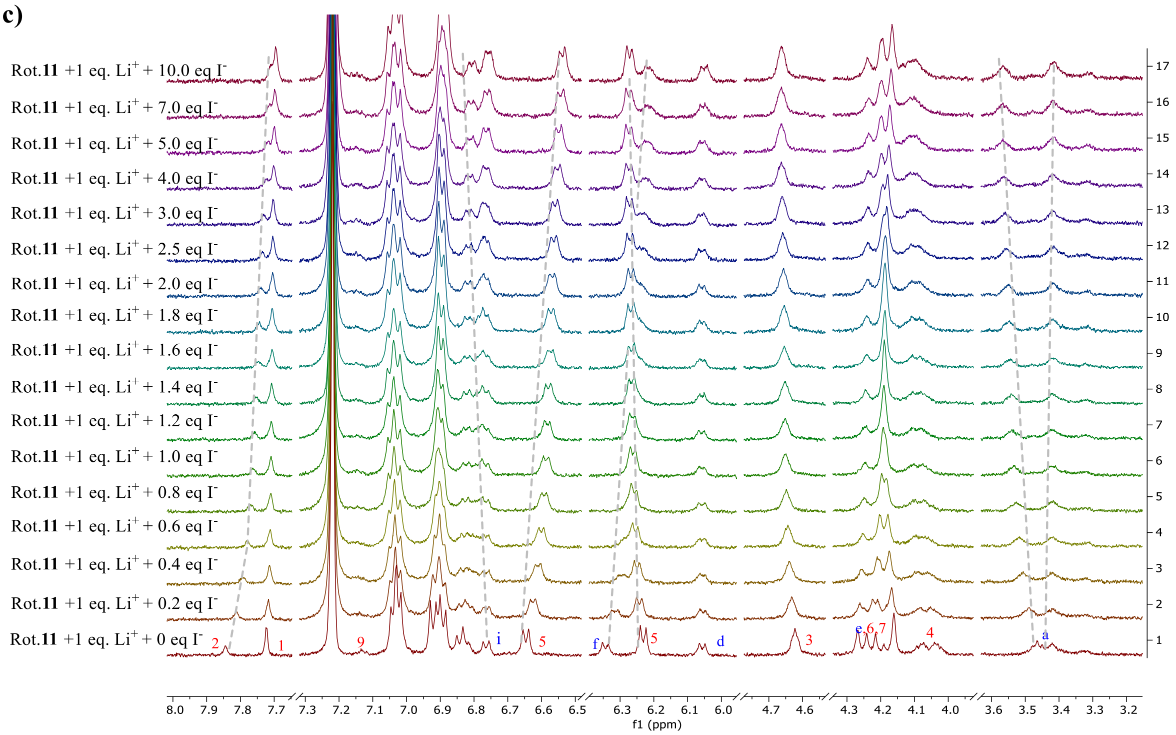

*Figure S3.4: Truncated ^{1}H NMR titration spectra of lithium precomplexed rotaxane **11** upon addition of (a)TBACl, (b)TBABr* and (c) TBAI (500 MHz, 298 K, 7:3 $CDCl_3$:CD_3CN, [Rot.**11**] = 1.0 mM). *precipitated after the addition of 7 eq. (Connecting lines are to guide the eye only)*

^{1}H NMR titrations of macrocycle **6**

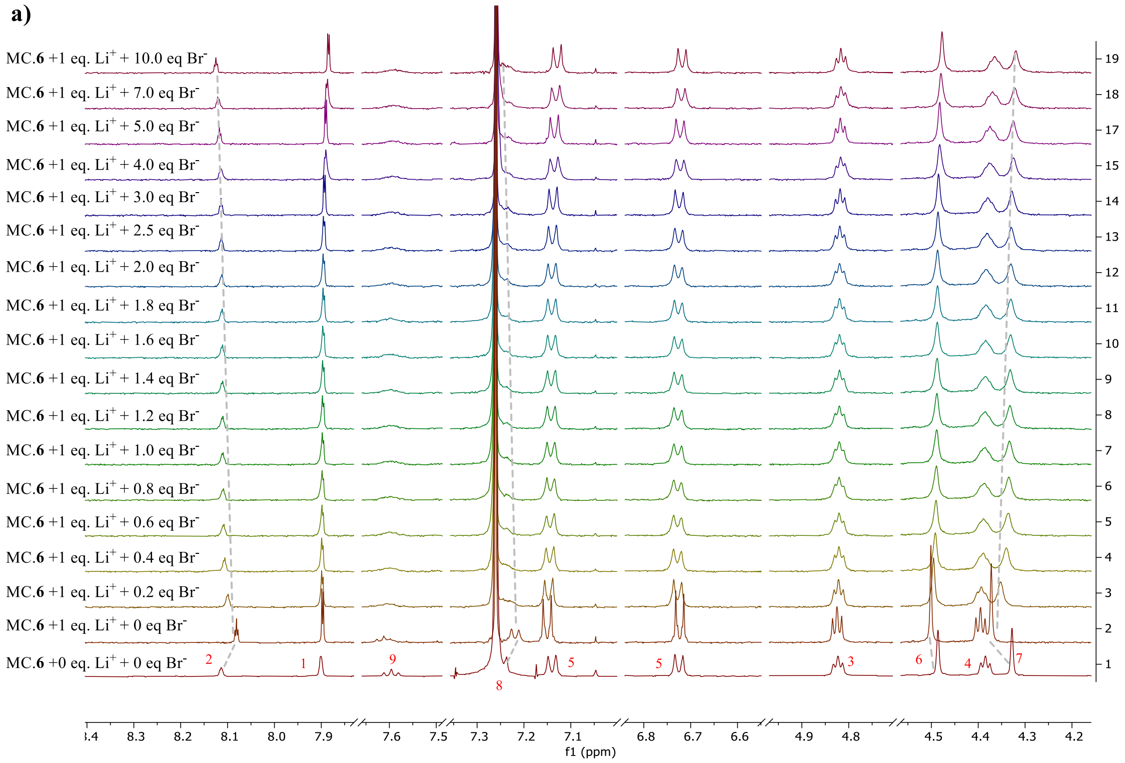

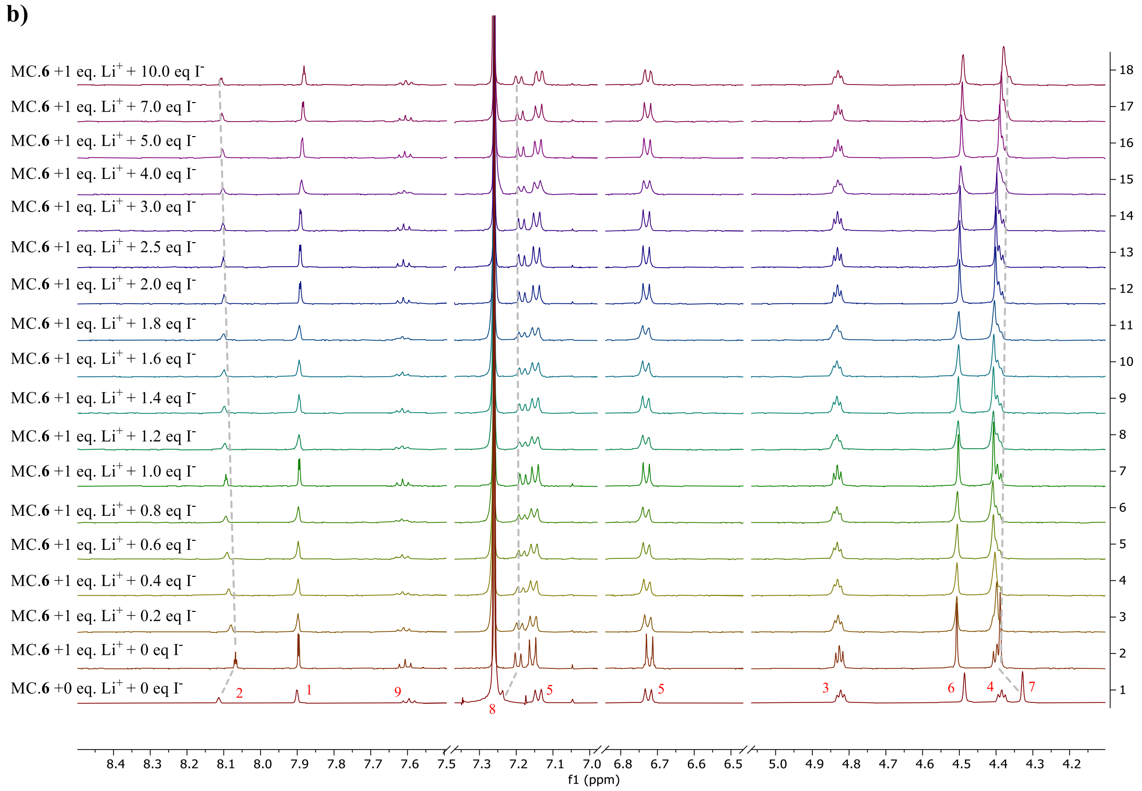

*Figure S3.5: Truncated ^{1}H NMR titration spectra of lithium precomplexed macrocycle **6** upon addition of (a)TBABr*, (b) TBAI* (500 MHz, 298 K, 9:1 $CDCl_3$:CD_3CN, [MC.**6**] = 1.0 mM). *^{1}H NMR peak shifts caused due to complexation of Li^{+}, reverted towards their original positions indicating metal decomplexation.*

^{1}H NMR titrations of macrocycle **5**

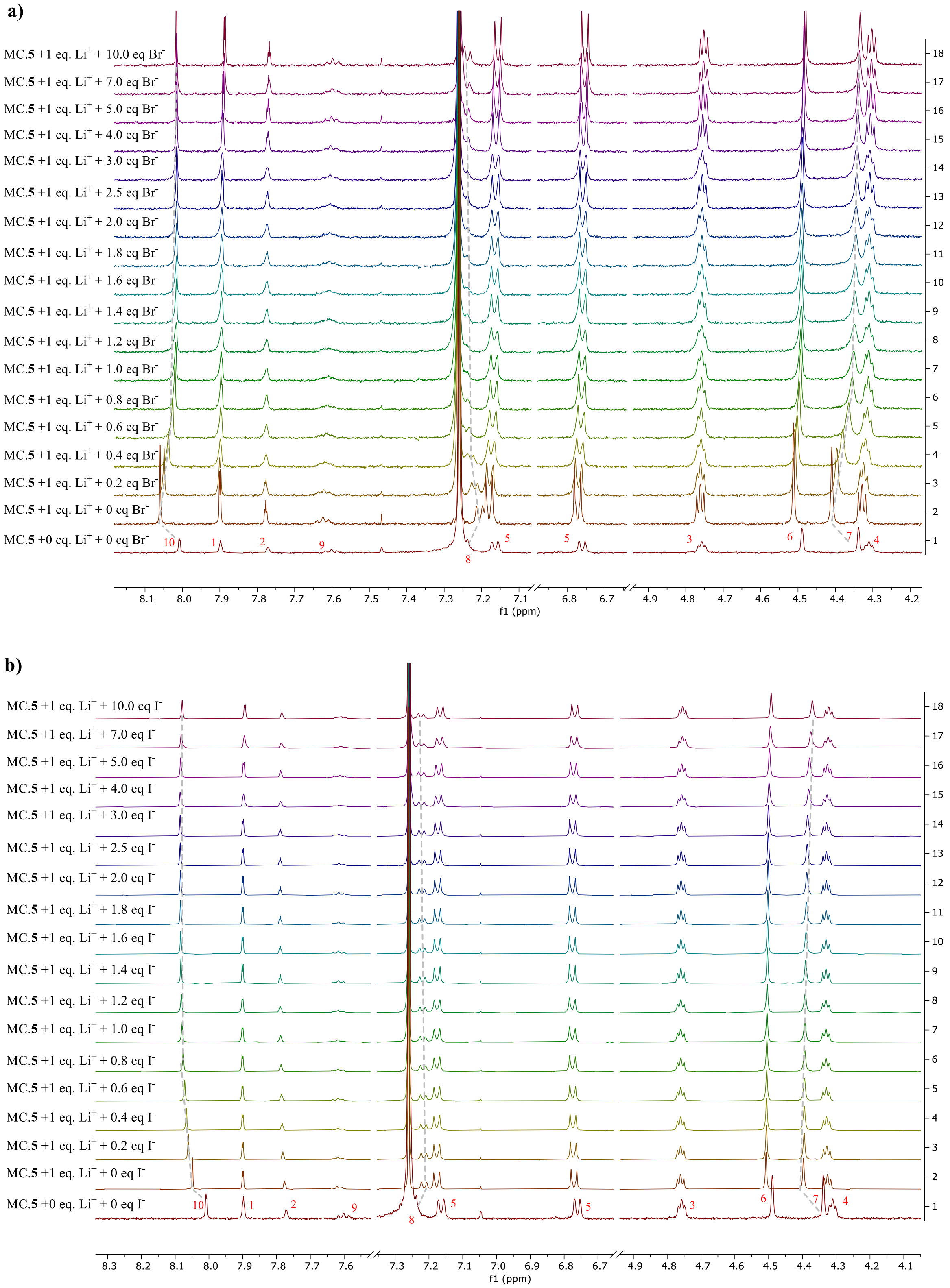

*Figure S3.6: Truncated ^{1}H NMR titration spectra of lithium precomplexed macrocycle **5** upon addition of (a)TBABr*, (b) TBAI* (500 MHz, 298 K, 9:1 $CDCl_3$:CD_3CN, [MC.**5**] = 1.0 mM). *^{1}H NMR peak shifts caused due to complexation of Li^+, reverted towards their original positions indicating metal decomplexation.*

S3.3. Cation binding ^{1}H NMR titration experiments

Sodium and lithium cation binding properties were investigated in the HB and XB rotaxane **10.** All rotaxanes have a similar cation binding site and was presumed to follow a similar cation binding trend. ^{1}H NMR cation binding titration experiments were conducted following a similar procedure stated in Section S3.1. Rotaxane **10** was dissolved in a 7:3, $CDCl_3$:CD_3CN solution to make a 0.5 mL of 1 mM rotaxane **10** solution. $LiClO_4$ was added as the Li^+ source and NaBArF as the Na^+ source. Due to low solubility of $LiClO_4$, a more diluted 0.025 M solution of $LiClO_4$ was prepared using the same $CDCl_3$:CD_3CN solvent system. Prepared cation solution was sequentially added to the rotaxane solution while recording the ^{1}H NMR spectra with each addition. Titration spectra was only recorded till the addition of 2.0 eq of $LiClO_4$ due to precipitation of Rotaxane-Li complex.

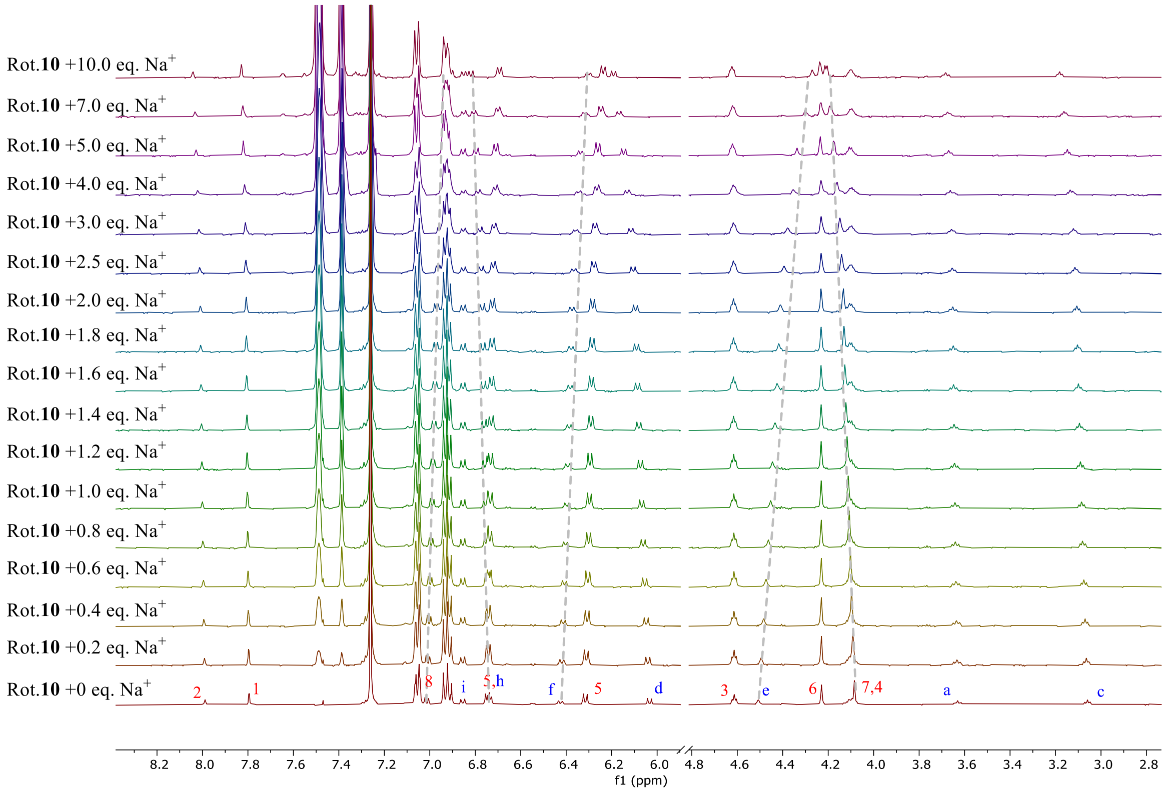

*Figure S3.7: Truncated ^{1}H NMR titration spectra of rotaxane **10** upon addition of increasing equivalents of NaBArF (500 MHz, 298 K, 7:3 $CDCl_3$:CD_3CN, [Rot.**10**] = 1.0 mM). (Connecting lines are to guide the eye only)*

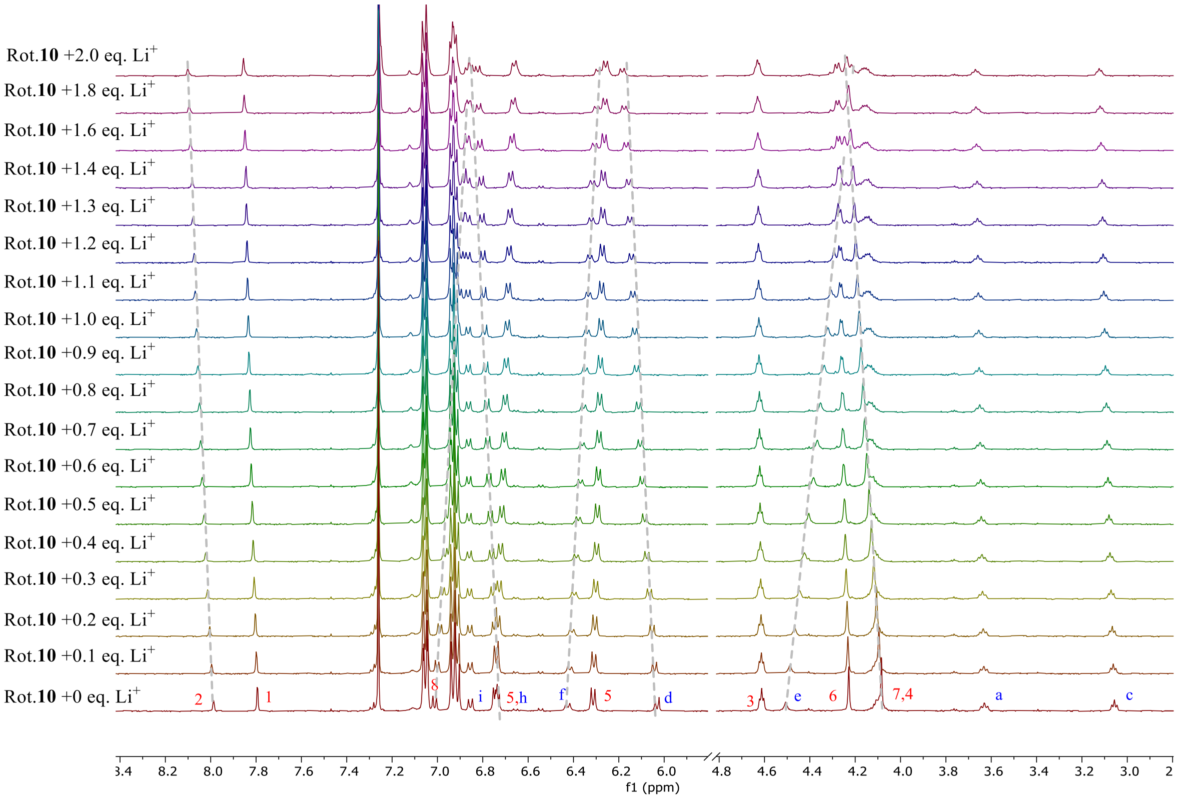

*Figure S3.8: Truncated ^{1}H NMR titration spectra of rotaxane **10** upon addition of $LiClO_4$* (500 MHz, 298 K, 7:3 $CDCl_3$:CD_3CN, [Rot.**10**] = 1.0 mM). (Connecting lines are to guide the eye only) *Titration spectra was only recorded till the addition of 2.0 eq. of $LiClO_4$ due to precipitation of Rotaxane-Li complex.*

According to Figure S3.7 and Figure S3.8 addition of cation caused significant perturbations in protons H_7, H_8, H_e, H_f in the cation binding site while protons in the anion binding site, H_a, H_c, H_3 remained unperturbed. This observation suggests that while Li^+ and Na^+ binds to the predicted cation binding cavity, $BArF^-$ and ClO_4^- acts as non-coordinating counter anions.

In typical lithium ion-pair binding titration experiments, rotaxanes are initially precomplexed with Li^+ by adding 1 equivalent of $LiClO_4$ to the rotaxane. Hence, attention was given to quantify the amount of Li^+ bound to the rotaxane with the addition of 1 equivalent of $LiClO_4$. To this end, a global BindFit analysis was conducted by monitoring non-overlapping peak shifts of H_2, H_f , H_d (Figure S3.8) to calculate association constant of Li^+ with rotaxane **10**.

$K_a(Li^+) = 691\ M^{-1}$

Upon addition of 1.0 equivalent of Li^+, suppose lithium complexed rotaxane moles = x

solvent volume in the NMR tube = v = 0.0005 L

R.**10** moles in NMR tube: 5×10^{-7} mol

$$R.10. + Li^+ \leftrightharpoons R.10\text{-}Li\ complex$$

	**Moles (mol) in NMR tube**		
	free **R.10**	free Li^+	**R.10**-Li complex
**Before adding 1 eq Li^+**	5×10^{-7}	-	-
**After adding 1 eq Li^+**	(5×10^{-7}) - x	(5×10^{-7}) - x	x

$$Ka = \frac{[\boldsymbol{R}.\mathbf{10} - Li]}{[\boldsymbol{R}.\mathbf{10}][Free\ Li^{+}]}$$

$$Ka = \frac{x/_{v}}{\left((5 * 10^{-7} - x)/_{v}\right)^{2}}$$

Solutions of the above quadratic equation, x: x'= 1.6×10^{-7} mol and x''=1.5×10^{-6} mol
As x''>5×10^{-7} mol, x=x'= 1.6×10^{-7} mol

$$lithium\ complexed\ rotaxane\ \% = \left(\frac{1.6*10^{-7}}{5*10^{-7}}\right) \times 100\% = 32\%$$

Upon the addition of 1.0 equivalent of $LiClO_4$, 32% of rotaxane complexes with Li^+, while the rest remains at equilibrium between both the free rotaxane and the cation. Anion binding constants were calculated assuming quantitative binding of Li^+ with the addition of 1.0 equivalent of $LiClO_4$. As evident by experimental anion and ion-pair binding titrations and DFT calculations, complexation of Li^+ increases anion binding constants through both electrostatic effects and by preorganising the anion binding site.

S4. DFT calculations

DFT calculations were carried out in the gas phase using Gaussian 16[10] at the B3LYP level of theory[11] using aug-cc-pVTZ basis set for chlorine atoms, aug-cc-pVTZ-PP basis set for larger halide atoms, and 6-31g* basis for the other atoms.[12–14] Counterpoise correction was invoked to correct for basis set superposition error.[15,16] Crystal structure of macrocycle **6** and axle[17] were used as starting coordinates. Terminal benzene moieties of the axles were replaced by methyl groups for the ease of simulation.

S4.1. DFT optimised structures

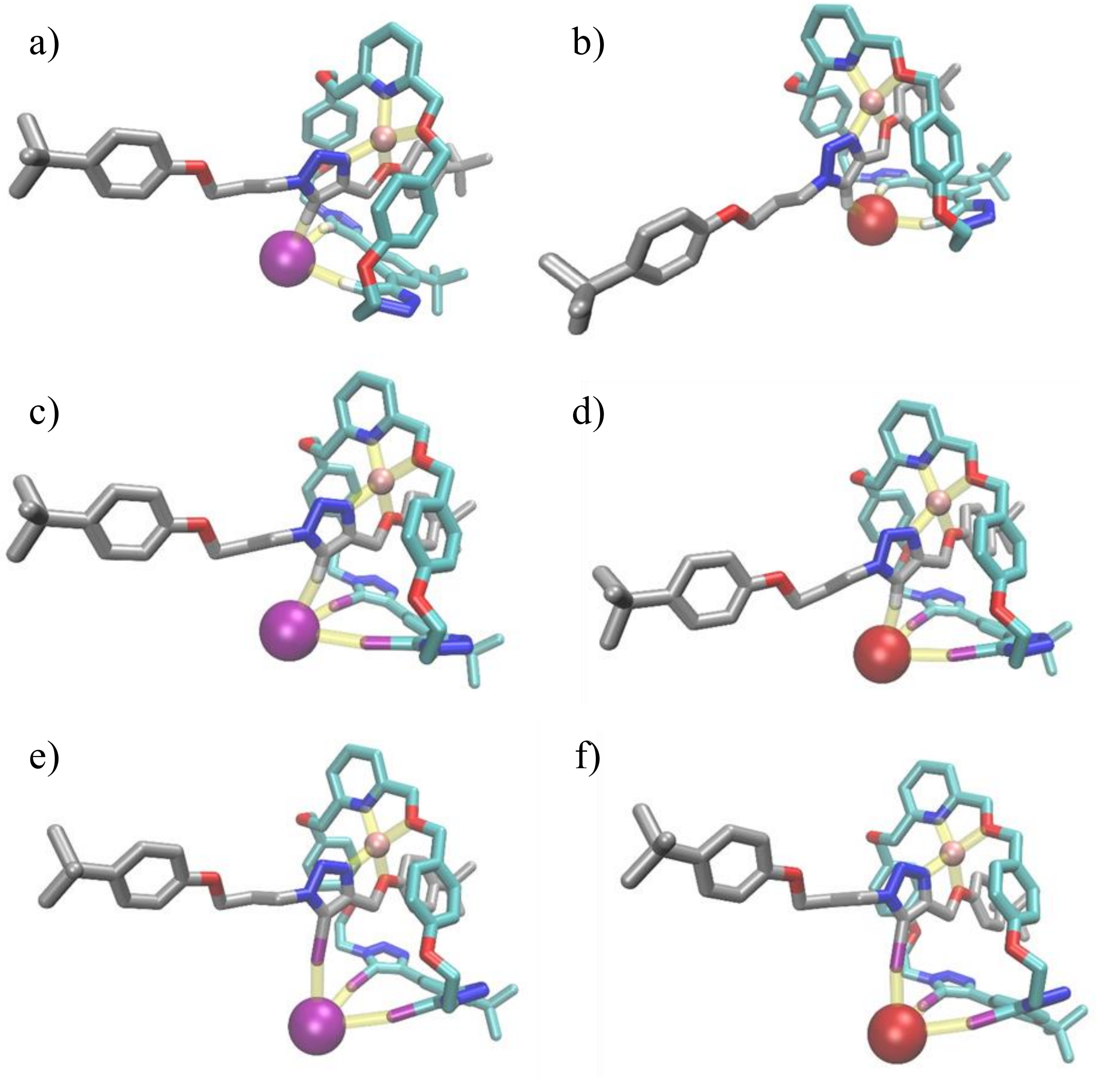

*Figure S4.1: DFT optimised ion-pair bound rotaxane structures; a) LiI bound rotaxane **9**, b) LiBr bound rotaxane **9**, c) LiI bound rotaxane **10**, d) LiBr bound rotaxane **10**, e) LiI bound rotaxane **11**, f) LiBr bound rotaxane **11**. All noninteracting H atoms are omitted for clarity. Colour code of atoms: O-red, N-blue, I-purple, Li-pink, Br- brown, Cl-green, $C_{(axle)}$ -gray $C_{(MC)}$-teal. Non-covalent host-guest interactions are shown by yellow*

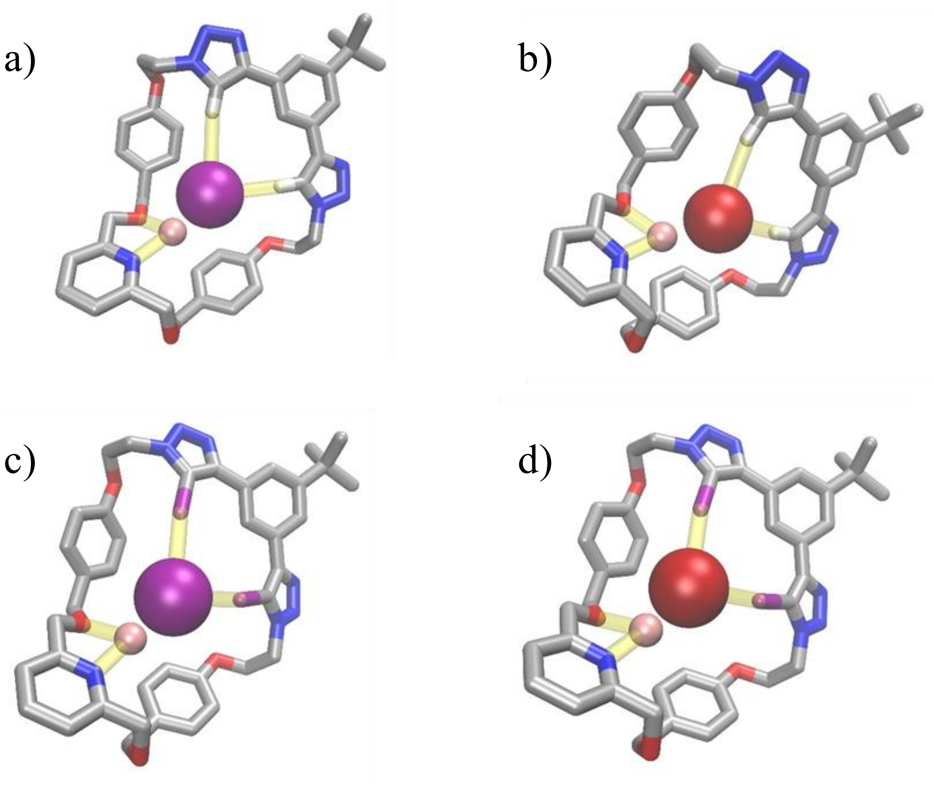

*Figure S4.2: DFT optimised ion-pair bound macrocycle structures: a) LiI bound macrocycle **5**, b) LiBr bound macrocycle **5**, c) LiI bound macrocycle **6**, d) LiBr bound macrocycle **6.** All noninteracting H atoms are omitted for clarity. Colour code of atoms: O-red, N-blue, I-purple, Li-pink, Br- brown, $C_{(MC)}$-teal. Non-covalent host-guest interactions are shown by yellow*

S4.2. Comparison of optimised structures with ^{1}H NMR data

Optimised host-guest binding modes agrees with the corresponding ^{1}H NMR titration data and are discussed below.

Rotaxane 9

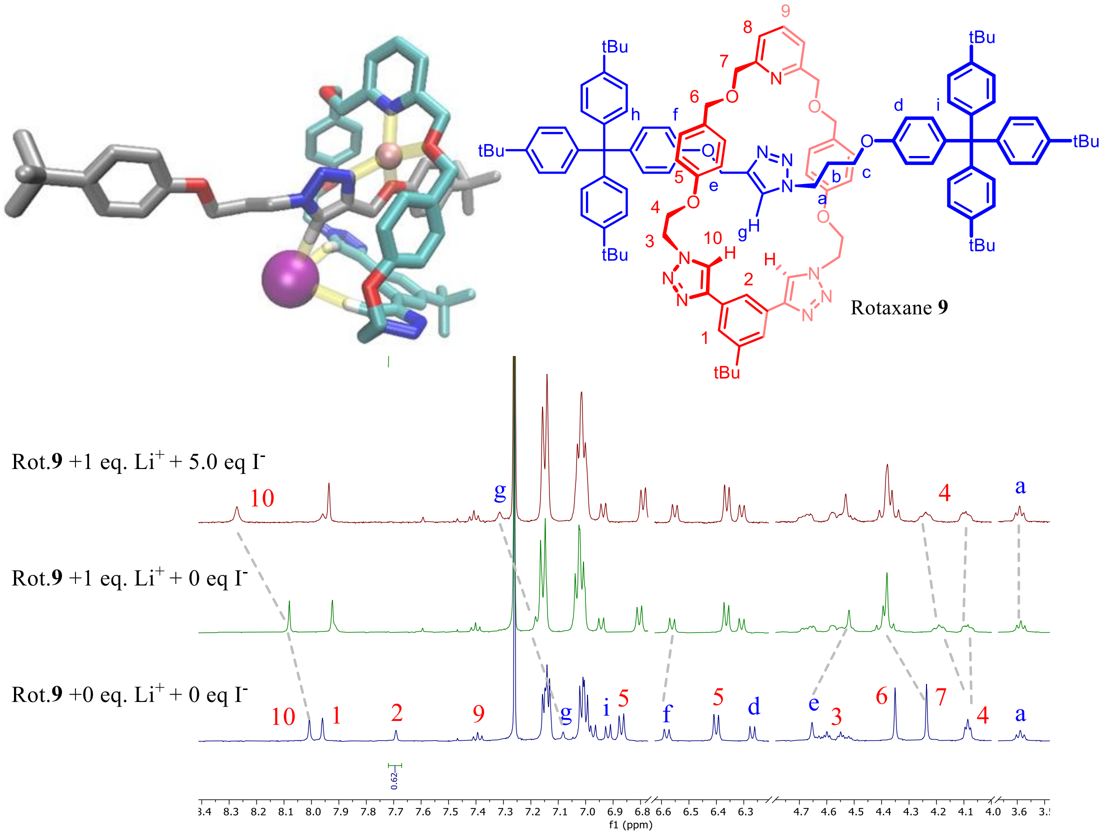

*Figure S4.3:Comparison of optimised ion-pair bound rotaxane **9** with corresponding ^{1}H NMR data (500 MHz, 298 K, 9:1 $CDCl_3$:CD_3CN, [Rot.**9**] = 1.0 mM)*

According to the optimised structure, Li^+ interacts with pyridine nitrogen and the adjacent oxygen of the macrocycle and with the triazole nitrogen and neighbouring oxygen in the axle. ^{1}H NMR data depicted above are consistent with the optimised structure. Axle proton H_e which is in between the coordinating triazole nitrogen and ether oxygen and proton H_f which is next to axle oxygen, shows a significant up field shift upon Li^+ complexation. Macrocyclic proton H_7, proximal to coordinating pyridine nitrogen, shifts downfield.

Anion in the optimised structure, forms HB interactions with all three proto triazoles in both the axle and the macrocycle. In accordance with the optimised data, all three proto triazole signals, H_{10} and H_g shows significant downfield shifts with the addition of an anion.

Rotaxane 10

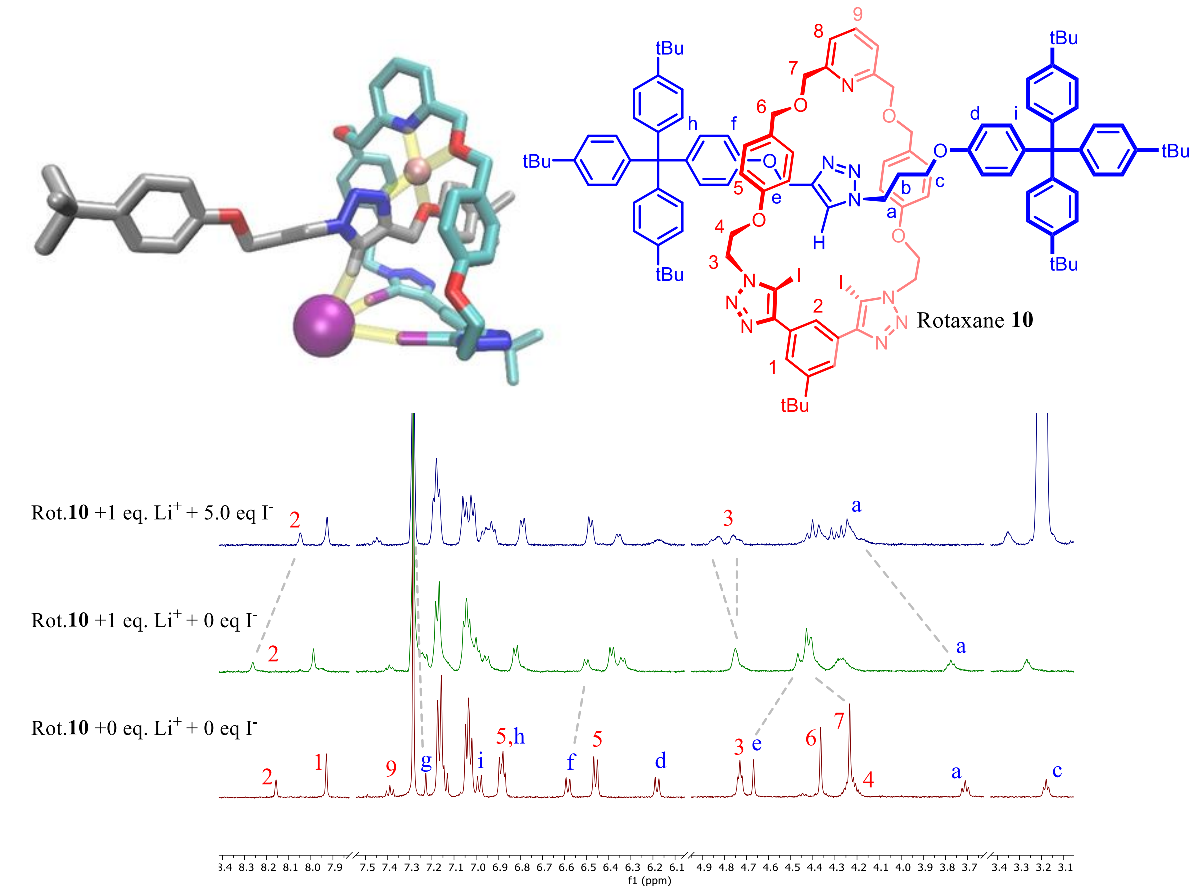

*Figure S4.4: Comparison of optimised ion-pair bound rotaxane **10** with corresponding ^{1}H NMR data (500 MHz, 298 K, 9:1 $CDCl_3$:CD_3CN, [Rot.**10**] = 1.0 mM)*

Similar to the ^{1}H NMR perturbations observed in rotaxane **9**, protons near the cation binding site H_e, H_f and H_7 show significant ^{1}H NMR perturbations with the addition of $LiClO_4$. Addition of the anion causes shifts in proton H_g, H_3 and H_a which are near to the optimised anion binding site. Importantly, H_3 and H_a peaks which are close to the anion binding site only perturb with the addition of TBAI and not with the addition of $LiClO_4$, indicating that ClO_4^- acts as a noncoordinating counter anion.

Rotaxane 11

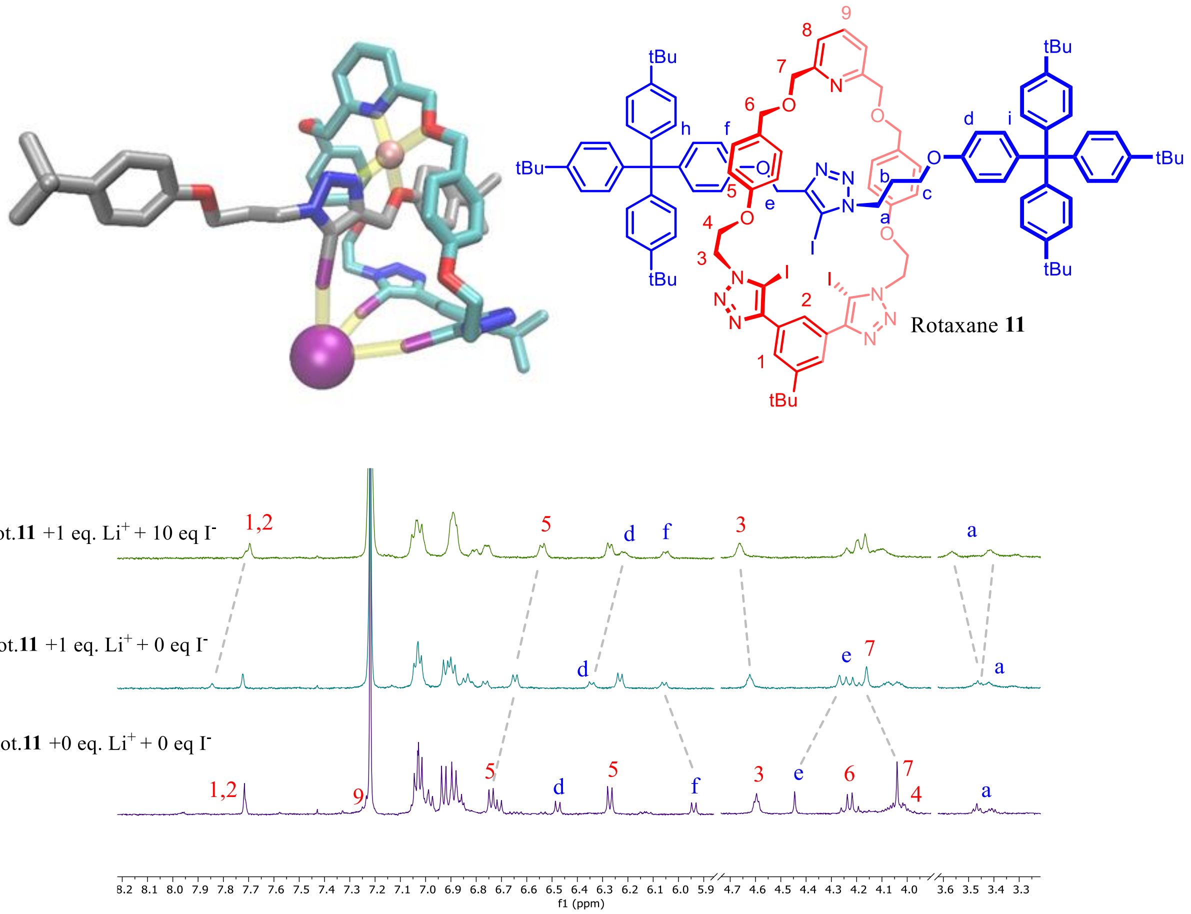

*Figure S4.5: Comparison of optimised ion-pair bound rotaxane **11** with corresponding ^{1}H NMR spectra (500 MHz, 298 K, 7:3 $CDCl_3$:CD_3CN, [Rot.**11**] = 1.0 mM)*

Upon addition of $LiClO_4$, protons near the optimised cation binding site, H_e, H_f and H_7 show significant ^{1}H NMR shifts. Addition of an anion results in significant peak shifts of protons, H_3, H_5, H_a, H_d, near the optimised anion binding site. Importantly, H_3 and H_a peaks which are close to the anion binding site only perturb with the addition of TBAI and not with the addition of $LiClO_4$, indicating that ClO_4^- acts as a noncoordinating counter anion.

Macrocycle 6

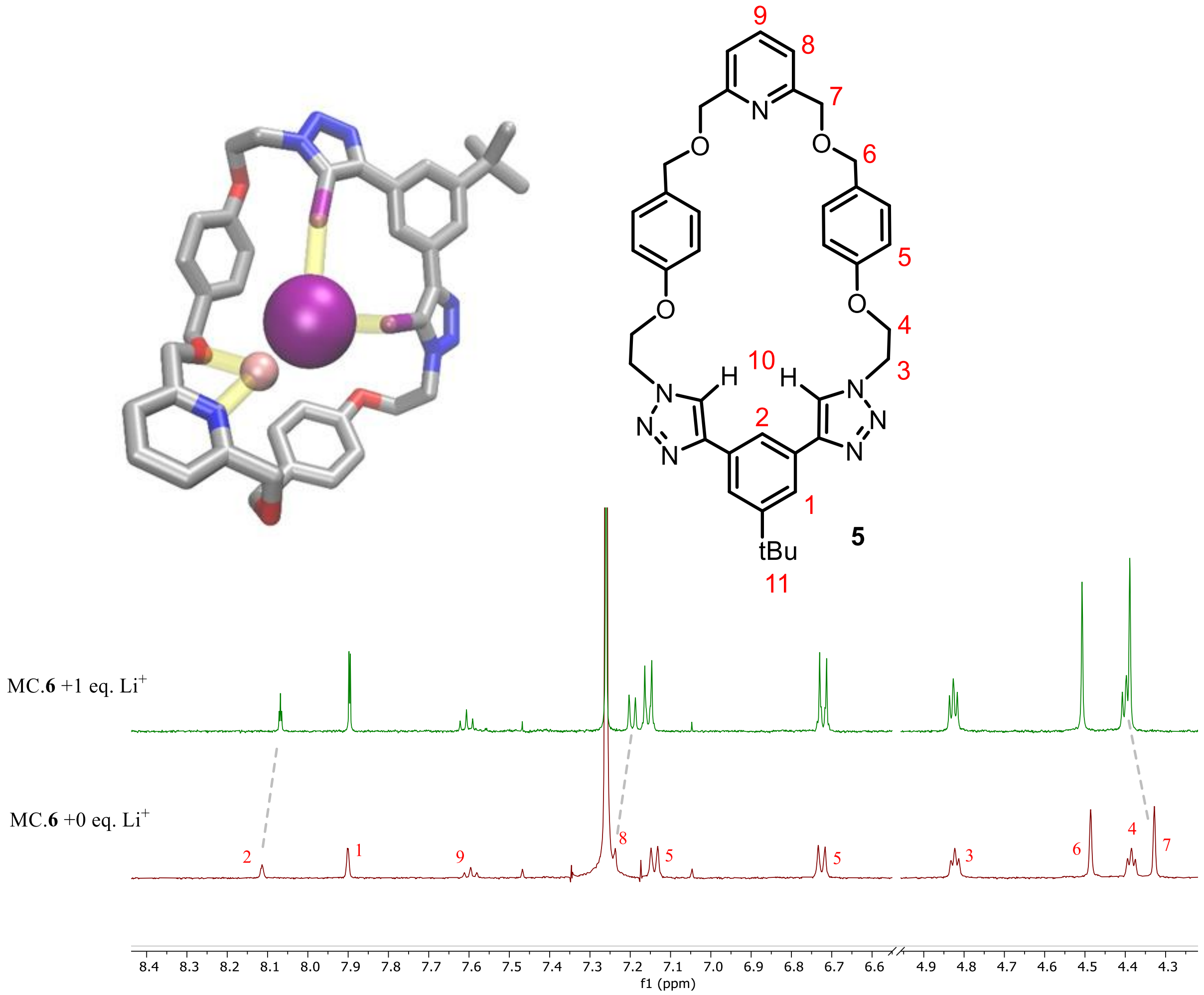

*Figure S4.6: Comparison of optimised lithium complexed macrocycle **6** with corresponding ^{1}H NMR data (500 MHz, 298 K, 9:1 $CDCl_3$:CD_3CN, [MC.**6**] = 1.0 mM)*

According to the optimised structure, Li^+ interacts with the pyridine nitrogen and adjacent oxygen of the macrocycle. Corresponding to the optimised structure, protons near the cation binding site, H_8 and H_7 showed significant perturbations upon addition of one equivalent of $LiClO_4$. Addition of TBABr and TBAI caused ^{1}H NMR peak shifts to revert towards its original position of the uncomplexed rotaxane indicating external lithium iodide ion-pair complexation (Figure S3.5).

Macrocycle 5

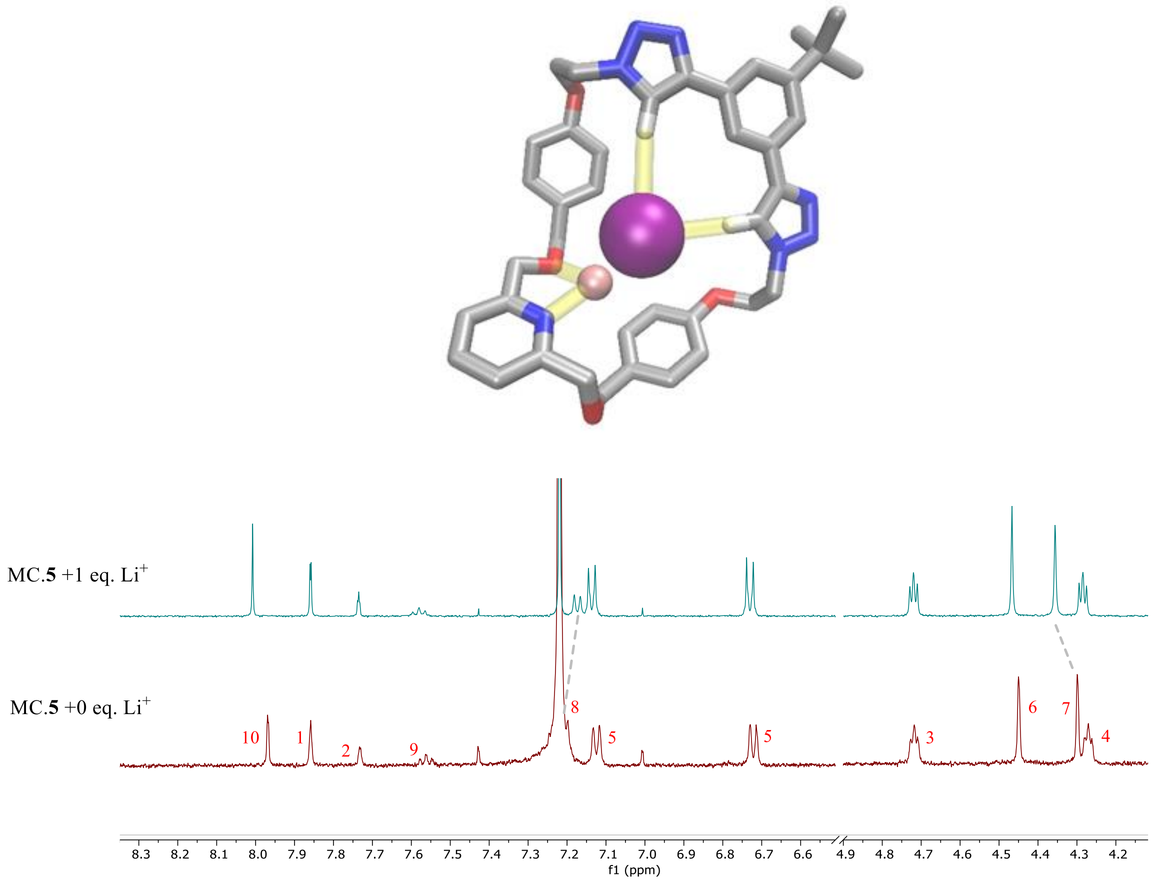

Figure S4.7: Comparison of optimised lithium complexed macrocycle ***5*** *with corresponding ^{1}H NMR data (500 MHz, 298 K, 9:1 $CDCl_3$:CD_3CN, [MC.**5**] = 1.0 mM)*

Similar to macrocycle **6,** protons near the optimised cation binding site, H_8 and H_7 showed significant perturbations upon addition of one equivalent of the cation. Addition of TBAI/TBABr caused ^{1}H NMR peak shifts to revert towards its original position of the uncomplexed rotaxane indicating external lithium iodide ion-pair complexation.

S4.3. Calculated association constants

Table S4.1: Calculated Gas-Phase Binding Energy for ***9***, ***10***, ***11*** *rotaxanes and* ***5***, ***6*** *macrocycles in kcal/mole.*

Anion	Cation	R.9	R.10	R.11	M.5	M.6
Br^-	Li^+	-213.96	-218.99	-216.29	-188.75	-189.8
I^-	Li^+	-208.77	-212.96	-210.72	-180.86	181.66

S4.4. Electrostatic potential map

It was postulated that the Li^+ cation complexes with the triazole nitrogen atom in the axle, polarising the connecting HB/XB donor atoms. Hence, rotaxane **10** and **11** which have HB and XB triazoles donors respectively in axle were chosen to map the ESP surface to investigate the effect of Li^+ complexation towards the electrostatic potential of the HB/XB donors atoms.

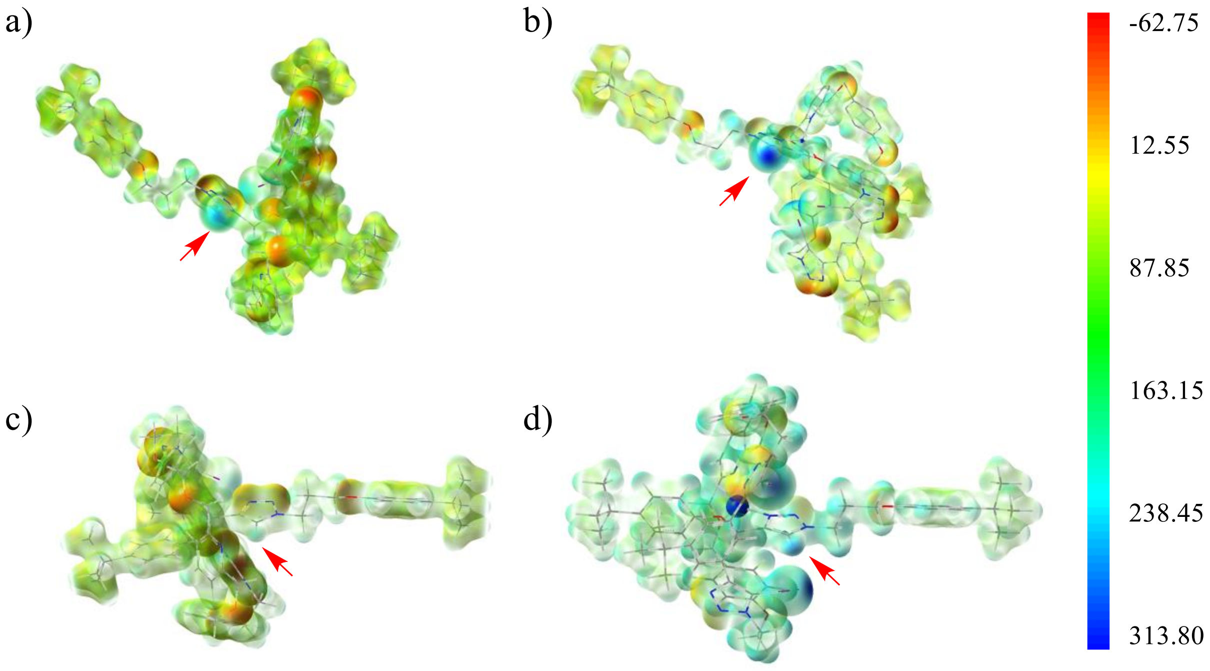

*Figure S4.8: Electrostatic potential maps of a) rotaxane **11**, b) Li^+ complexed rotaxane **11**, c) rotaxane **10**, d) Li^+ complexed rotaxane **10**. Arrow indicates the triazole XB donor atom and HB donor atoms of rotaxane **11** and **10** respectively. Colour coded isosurface values are given in kcal/mol.*

S5. References

[1] Y. Tian, J. Jin, C. Wang, W. Lv, X. Li, X. Che, Y. Gong, Y. Li, Q. Li, J. Hou, P. G. Wang, J. Shen, *Bioorg. Med. Chem. Lett.* **2016**, *26*, 2434–2437.

[2] Y. C. Tse, A. Docker, Z. Zhang, P. D. Beer, *Chem. Commun.* **2021**, *57*, 4950–4953.

[3] G. Ye, J. Roques, P.-L. Solari, C. Den Auwer, A. Jeanson, J. Brandel, L. J. Charbonnière, W. Wu, É. Simoni, *Inorg. Chem.* **2021**, *60*, 2149–2159.

[4] V. Aucagne, K. D. Hänni, D. A. Leigh, P. J. Lusby, D. B. Walker, *J. Am. Chem. Soc.* **2006**, *128*, 2186–2187.

[5] N. L. Kilah, M. D. Wise, C. J. Serpell, A. L. Thompson, N. G. White, K. E. Christensen, P. D. Beer, *J. Am. Chem. Soc.* **2010**, *132*, 11893–11895.

[6] CrysAlisPRO, *Oxford Diffr. /Agilent Technol. UK Ltd, Yarnton, Engl.*

[7] A. Thorn, G. M. Sheldrick, *Acta Cryst* **2008**, *64*, C221--222.

[8] G. M. Sheldrick, *Acta Crystallogr. Sect. C Struct. Chem.* **2015**, *71*, 3–8.

[9] P. Thordarson, *Chem. Soc. Rev.* **2011**, *40*, 1305–1323.

[10] M. J. Frisc, G. W. Trucks, H. B. Schlegel, G. E. Scuseria, M. A. Robb, J. R. Cheeseman, G. Scalmani, V. Barone, B. Mennucci, G. A. Petersson, others, *Gaussian Inc, Wallingford* **2010**.

[11] A. D. Becke, *J. Chem. Phys.* **1993**, *98*, 5648–5652.
[12] D. E. Woon, T. H. Dunning, *J. Chem. Phys.* **1993**, *98*, 1358–1371.
[13] K. A. Peterson, B. C. Shepler, D. Figgen, H. Stoll, *J. Phys. Chem. A* **2006**, *110*, 13877–13883.
[14] K. A. Peterson, D. Figgen, E. Goll, H. Stoll, M. Dolg, *J. Chem. Phys.* **2003**, *119*, 11113–11123.
[15] S. F. Boys, F. Bernardi, *Mol. Phys.* **1970**, *19*, 553–566.
[16] F. B. van Duijneveldt, J. G. C. M. van Duijneveldt-van de Rijdt, J. H. van Lenthe, *Chem. Rev.* **1994**, *94*, 1873–1885.
[17] Y. Miyazaki, C. Kahlfuss, A. Ogawa, T. Matsumoto, J. A. Wytko, K. Oohora, T. Hayashi, J. Weiss, *Chem. – A Eur. J.* **2017**, *23*, 13579–13582.
